# Post-main-sequence planetary system evolution

**DOI:** 10.1098/rsos.150571

**Published:** 2016-02-17

**Authors:** Dimitri Veras

**Affiliations:** Department of Physics, University of Warwick, Coventry CV4 7AL, UK

**Keywords:** dynamics, white dwarfs, giant branch stars, pulsars, asteroids, formation

## Abstract

The fates of planetary systems provide unassailable insights into their formation and represent rich cross-disciplinary dynamical laboratories. Mounting observations of post-main-sequence planetary systems necessitate a complementary level of theoretical scrutiny. Here, I review the diverse dynamical processes which affect planets, asteroids, comets and pebbles as their parent stars evolve into giant branch, white dwarf and neutron stars. This reference provides a foundation for the interpretation and modelling of currently known systems and upcoming discoveries.

## 1. Introduction

Decades of unsuccessful attempts to find planets around other Sun-like stars preceded the unexpected 1992 discovery of planetary bodies orbiting a pulsar [1,2]. The three planets around the millisecond pulsar PSR B1257+12 were the first confidently reported extrasolar planets to withstand enduring scrutiny due to their well-constrained masses and orbits. However, a retrospective historical analysis reveals even more surprises. We now know that the eponymous celestial body that Adriaan van Maanen observed in the late 1910s [3,4] is an isolated white dwarf (WD) with a metal-enriched atmosphere: direct evidence for the accretion of planetary remnants.

These pioneering discoveries of planetary material around or in post-main-sequence (post-MS) stars, although exciting, represented a poor harbinger for how the field of exoplanetary science has since matured. The first viable hints of exoplanets found around MS stars (*γ* Cephei Ab and HD 114762 b) [5,6], in 1987–1989, were not promulgated as such due to uncertainties in the interpretation of the observations and the inability to place upper bounds on the companion masses. A confident detection of an MS exoplanet emerged with the 1995 discovery of 51 Pegasi b [7], followed quickly by 70 Virginis b [8] and 47 Ursae Majoris b [9], although in all cases the mass degeneracy remained. These planets ushered in a burgeoning and flourishing era of astrophysics. Now, two decades later, our planet inventory numbers in the thousands; over 90% of all known exoplanets orbit MS stars that will eventually become WDs, and WDs will eventually become the most common stars in the Milky Way.

Nevertheless, major uncertainties linger. MS exoplanet detection techniques currently provide minimal inferences about the bulk chemical composition of exoplanetary material. How planets form and dynamically settle into their observed states remains unanswered and represents a vigorous area of active research. Calls for a better understanding of post-MS evolution arise from MS discoveries of planets near the end of their lives [10] and a desire to inform planet formation models [11]. Direct observation of MS smaller bodies, such as exo-asteroids, exo-comets or exo-moons, remains tantalizingly out of reach, except in a handful of cases [12–16].

Post-MS planetary system investigations help alleviate these uncertainties, particularly with escalating observations of exoplanetary remnants in WD systems. Unlike for pulsar systems, planetary signatures are common in and around WD stars. The exquisite chemical constraints on rocky planetesimals that are gleaned from WD atmospheric abundance studies is covered in detail by the review of Jura & Young [17], and is not a focus of this article. Similarly, I do not focus on the revealing observational aspects of the nearly forty debris discs orbiting WDs, a topic recently reviewed by Farihi [18].

Instead, I place into context and describe the complex and varied dynamical processes that influence planetary bodies after the star has turned off of the MS. I attempt to touch upon all theoretical aspects of post-MS planetary science, although my focus is on the giant branch (GB) and WD phases of stellar evolution. The vital inclusion of bodies smaller than planets—e.g. exo-asteroids and exo-comets—in this review highlights both the necessity of incorporating Solar system constraints and models and the interdisciplinary nature of post-MS planetary science.

### 1.1 Article layout

**Figure 1.**
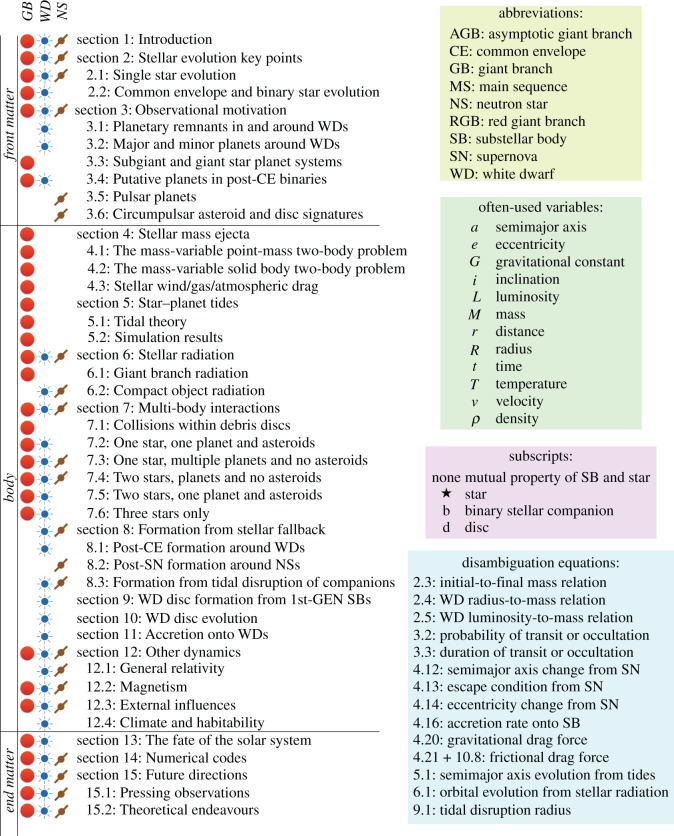
Paper outline and nomenclature. Some section titles are abbreviated to save space. Variables not listed here are described in situ, and usually contain descriptive subscripts and/or superscripts. The important abbreviation ‘substellar body’ (SB) can refer to, for example, a brown dwarf, planet, moon, asteroid, comet or pebble. ‘Disambiguation equations’ refer to relations that have appeared in multiple different forms in the literature. In this paper, these other forms are referenced in the text that surrounds these equations, so that readers can decide which form is best to use (or newly derive) for their purposes. Overdots always refer to time derivatives. The expression 〈 〉 refers to averaged quantities.

**Figure 2.**
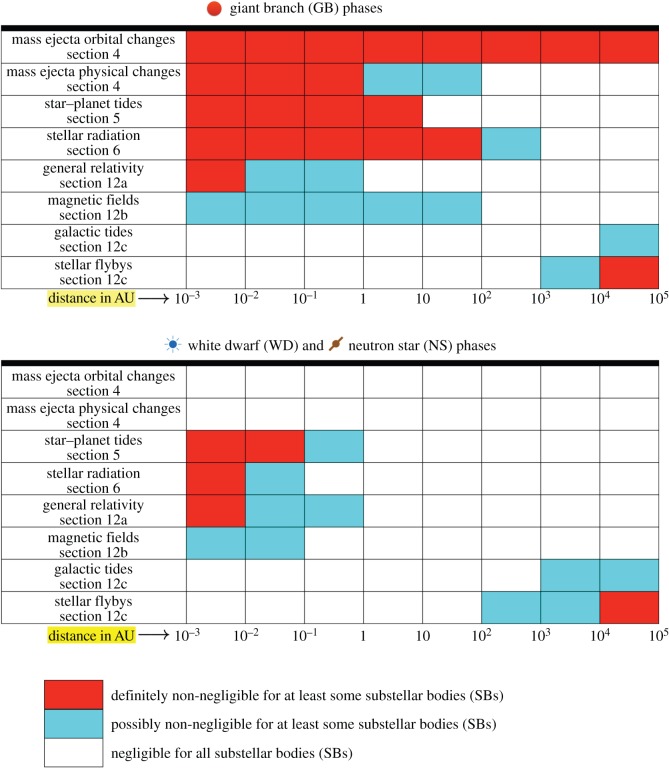
Important forces in post-MS systems. These charts represent just a first point of reference. Every system should be treated on a case-by-case basis. Magnetic fields include those of both the star and the SB, and external effects are less penetrative in the GB phases because they are relatively short.

I begin by providing a visual table of contents in figure 1, which includes handy references for the abbreviations and commonly used variables in this article. I use the abbreviation ‘SB’ (‘substellar body’ or ‘smaller body’) extensively in the text and equations; constraining relations to just one of planets, asteroids, comets or pebbles is too restrictive for the strikingly diverse field of post-MS planetary science. The term also includes brown dwarfs, for which many physical relations presented here also apply. ‘Planetary systems’ is defined as systems which include SBs. The ‘disambiguation’ equations identified in figure 1 refer to relations that have appeared in multiple different forms in the previous post-MS planetary literature; I attempt to consolidate these references. In figure 2, I characterize distances from the star in which various forces are important, or might be important. This figure may be used as a guide when modelling a particular system or set of systems. Table 1 lists some notable post-MS planetary systems, along with brief descriptions and pointers to where they are mentioned in the text.

**Table 1.** Some notable post-MS planetary systems.

name	type	sections
BD+48 740^a^	GB star with possible pollution	3.3.1
G 29-38^b^	WD with disc and pollution	3.1.2
GD 362^c^	WD with disc and pollution	3.1.2
GJ 86^d^	binary WD–MS with planet	3.2.2
NN Ser^e^	binary WD–MS with planets	3.4, 7.4.1, 7.4.2, 8.1, 15.1.1
PSR B1257+12^f^	pulsar with planets	1, 3.5, 7.3.3, 8.2, 8.3
PSR B1620-26^g^	binary pulsar-WD with planet	3.2.1, 7.4.1
SDSS J1228+1040^h^	WD with disc and pollution	3.1.2, 10, 15.1.2
WD 0806-661^i^	WD with planet	3.2.1
WD 1145+017^j^	WD with asteroids, disc and pollution	3.2.1., 15.1.1
WD J0959-0200^k^	WD with disc and pollution	3.1.2., 10, 15.1.1
vMa2^l^	WD with pollution	1, 3.1.1

^a^Potentially polluted with lithium.

^b^First WD debris disc.

^c^Polluted with 17 different metals.

^d^Planet orbits the MS star.

^e^Multiple circumbinary planets.

^f^First confirmed exoplanetary system.

^g^First confirmed circumbinary planet.

^h^Disc probably eccentric and axisymmetric.

^i^Planet at several thousand astronomical units.

^j^Only WD with transiting SBs, a disc and pollution.

^k^Highly variable WD disc.

^l^First polluted WD.

My deliberately basic treatment of introductory material (stellar evolution and observations from §§2 to 3) is intended to provide the necessary background for subsequent sections, and not meant to emulate an in-depth synopsis. The body of the article (§§4–12) provides more detail on the dynamical aspects of post-MS planetary science. This review concludes with brief comments on the fate of the Solar system (§13), a hopefully helpful summary of the numerical codes that have or may be used in theoretical investigations (§14) and a promising outlook on the future of this science (§15), with guidance for how upcoming observations can maximize scientific return.

## 2. Stellar evolution key points

The infrangible link between SBs and their host star is highlighted during post-MS evolution, and requires one to understand the star’s temporal evolution. My treatment below is purposefully simplified to provide the necessary information for post-MS planetary system studies; for more detail, see e.g. [19,20].

### 2.1 Single star evolution

#### 2.1.1 Main sequence

The MS evolution is important because it provides the historical context and initial conditions for dedicated post-MS studies. MS stars quiescently burn hydrogen to produce helium in their cores, and do lose mass through winds according to eqn 4 of [21] and eqn 9 of [22]. The Sun currently loses mass at a rate of about 2.4×10^−14^ *M*_⊙_ yr^−1^ (p. 15 of [23]). The MS lifetime is sensitively dependent on the initial value of $M_{⋆}^{\left(\mathrm{MS}\right)}$ and less so on the star’s metallicity *Z*_★_. This lifetime decreases drastically (by two orders of magnitude, from about 10 to 0.1 Gyr) as the initial mass increases from 1 to 6 *M*_⊙_ (see fig. 5 of [24]).

#### 2.1.2 Giant branches

All stars experience the ‘red giant branch’ (RGB) phase, when hydrogen in the core is exhausted and the remaining hydrogen burns in a contracting shell as the envelope expands. The extent of convection in the star increases, potentially ‘dredging-up’ already-burnt matter. Eventually, core temperatures become high enough to burn helium. For stars with $M_{⋆}^{\left(\mathrm{MS}\right)}<2.0\;M_{⊙}$, helium ignition sets off so-called ‘helium flashes’. This value of 2.0 *M*_⊙_ represents a key transition mass; the duration and character of the mass loss changes markedly when crossing this threshold. After the core helium is exhausted, a helium-burning shell is formed. At this point, the star is said to have begun evolving on the ‘asymptotic giant branch’ (AGB). Another expansion of convection may then cause a ‘second dredge-up’. When, during the AGB, the helium-burning shell reaches the hydrogen outer envelope, a different type of helium flash occurs (denoted a ‘thermal pulse’), one which emits a sudden burst of luminosity and mass. This event, which can occur many times, also causes a sudden increase and then drop in stellar radius (see fig. 3 of [25]). Therefore, AGB thermal pulses literally cause the star to pulsate. Changes in the star’s convective properties during this violent time may also allow for a ‘third dredge-up’ to then occur.

**Figure 3.**
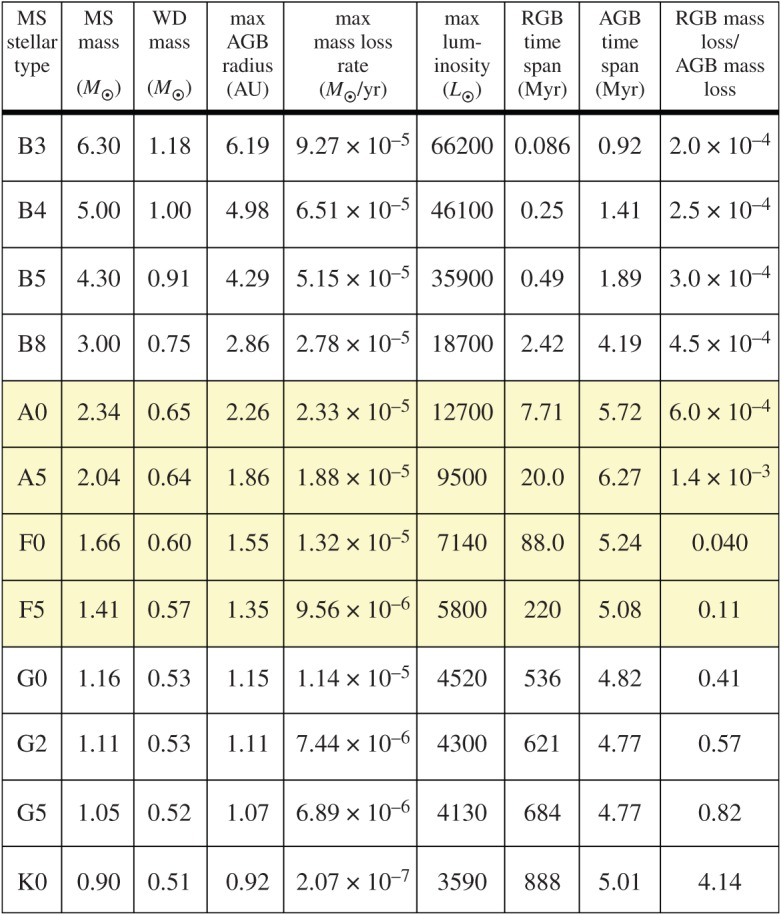
Useful values for 12 different stellar evolution tracks. I mapped the first column to the second by using appendix B of [26], and then created the remaining columns by using the sse code [27] by assuming its default values (which includes Solar metallicity). The four highlighted rows roughly represent the range of the most common progenitor stars for the present-day WD population in the Milky Way.

During both the RGB and AGB phases, the star undergoes significant mass loss (up to 80%), radius variability (up to about 10 AU, from an initial value of 10^−3^−10^−2^ AU), and luminosity variability (up to many tens of thousand times the MS value) regardless of the extent of the pulses. Figure 3 provides representative values; the highlighted rows indicate the most typical progenitors for the currently observed Milky Way WD population. These changes along the GB phases may completely transform a planetary system; indeed linking WD and MS planetary systems is a goal and a challenge, and may also help constrain stellar evolution. Unfortunately, identifying the dominant mechanisms responsible for mass loss—both isotropic and anisotropic—on the RGB and AGB continues to prove difficult.

*Red giant branch mass loss.* On the RGB, mass-loss is traditionally parametrized by the Reimers formula, a series of proportionalities that was later calibrated [28] and recently improved upon [29] to finally give

2.1 $$\begin{array}{rl} \frac{dM_{⋆}^{\left(\mathrm{RGB}\right)}}{dt} & =8\times 10^{-14}M_{⊙}\;{\mathrm{yr}}^{-1}\left(\frac{L_{⋆}^{\left(\mathrm{RGB}\right)}}{L_{⊙}}\right)\left(\frac{R_{⋆}^{\left(\mathrm{RGB}\right)}}{R_{⊙}}\right){\left(\frac{M_{⋆}^{\left(\mathrm{RGB}\right)}}{M_{⊙}}\right)}^{-1}\\ & \;\times {\left(\frac{T_{⋆}^{\left(\mathrm{RGB}\right)}}{4000\;K}\right)}^{7/2}\left[1+2.3\times 10^{-4}{\left(\frac{g_{⋆}^{\left(\mathrm{RGB}\right)}}{g_{⊙}}\right)}^{-1}\right], \end{array}$$

where *g* refers to surface gravity. Traditional formulations of equation (2.1), which are still widely used, do not include the final two terms, and have a leading coefficient of 2×10^−13^ *M*_⊙_ yr^−1^.

*Asymptotic giant branch mass loss.* Applying the Reimers formula on the AGB can produce significantly erroneous results (fig. 13 of [30]). Instead, during this phase a different prescription is often applied, whose formulation [31] has stood the test of ongoing observations and can be found, for example, in eqns 2–3 of [32]. Accompanying each AGB pulse is a variation in mass loss of potentially a few orders of magnitude, a phenomenon now claimed to have been observed [33]. At the final stage of the AGB—‘the tip’ of the AGB—the wind is particularly powerful and is known as the ‘superwind’ (e.g. [34]). A star’s peak mass loss rate typically occurs during the superwind unless the AGB phase is non-existent or negligible.

*Giant branch mass ejecta speed.* The speed at which mass is ejected is generally a function of the internal properties of the star and the location of ejection. One simplified estimate that may be useful for post-MS studies is [35]

2.2 $$v_{\mathrm{wind}}=\sqrt{\left(\frac{2GM_{⋆}}{R_{⋆}}\right)\left(1-\frac{{\left[v_{⋆}^{\left(\mathrm{rot}\right)}\right]}^{2}R_{⋆}}{GM_{⋆}}\sin^{2}\theta \right)},$$

where *θ* is the stellar co-latitude and $v_{⋆}^{\left(\mathrm{rot}\right)}$ is the stellar rotational speed at the equator.

*Giant branch changes from substellar body ingestion.* If a large SB such as a brown dwarf of planet is ingested during the GB phases, two significant events might result: an enhancement of lithium in the photosphere and spin-up of the star. The former was predicted in 1967 by Alexander [36]. Adamów *et al.* [37] claimed that SB accretion onto stars can increase their Li surface abundance for a few Myr. However, an enhanced abundance of Li in GB stars could also indicate dredge-up by the Cameron–Fowler mechanism, mixing through tides, or thermohaline or magneto-thermohaline processes. Therefore, a planetary origin interpretation for Li overabundance remains degenerate.

Several investigations have considered how a GB star spins up due to SB accretion. In his eqn 1, Massarotti [38] computed the change in the star’s rotational speed. He suggested that a population of GB fast-rotators due to planet ingestion would be detectable if the speed increased by at least 2 km s^−1^. Carlberg *et al.* [39] found that a few percent of the known population of exoplanets (at the time) could create rapid rotators, where rapid is defined as having a rotational speed larger than about 10 km s^−1^.

Substellar body ingestion may cause other changes, such as enhanced mass loss [40,41] and displacement on the Hertzsprung–Russel diagram [42]. The presence or ingestion of an SB could be a reason [43] why some GB stars prematurely lose their entire envelope before core fusion of helium begins. These stars are known as ‘extreme horizontal branch stars’, which are also known as ‘hot subdwarfs’ or ‘sdB’ stars (see [44] for a review specifically of these types of stars). This SB ingestion explanation is particularly relevant for hot subdwarfs with no known stellar binary companions. When modelling AGB envelopes for this or other purposes, one may use a power-law density profile (see, e.g. Sec. 2 [45]); Willes & Wu [46] instead gave a more complex form in their eqn 5.

#### 2.1.3 White dwarfs

For stars with $M_{⋆}^{\left(\mathrm{MS}\right)}≲8\;M_{⊙}$, after the GB envelope is completely blown away the remaining core becomes a WD [27,47,48]. In the Milky Way, about 95% of all stars will become WDs [47]. The term ‘white’ in WD originates from the notion that the majority of known WDs are hotter than the Sun [47]. The expelled material photoionizes and the resulting observed structure, which might not have any relation to planets whatsoever, is confusingly termed a ‘planetary nebula’. Although the expelled material will encounter remnant planets and asteroids, few investigations so far have tried to link these nebulae with planets. Even the link between nebula morphology and stellar configurations remains uncertain [49] although SBs that are at least as massive as planets, as well as stellar-mass companions, are thought to play a significant role in shaping and driving the nebulae (e.g. [50,51]).

The time elapsed since the moment an AGB star becomes a WD is denoted the ‘cooling age’ because the WD is in a state of monotonic cooling (as nuclear burning has now stopped). The term cooling age allows one to distinguish from the total age of the star, which includes its previous evolutionary phases. Although some investigations refer to planetary nebula or ‘post-AGB’ as the name of a separate stellar evolutionary phase [52,53], I do not, and assume that the transition from AGB to WD contains no other evolutionary phase.

*White dwarf designations.* WDs have and continue to be characterized observationally by the dominant spectral absorption lines in their atmospheres. These designations [54] include ‘D’, which stands for degenerate, ‘A’, for hydrogen rich, ‘B’ for helium rich, ‘Z’ for metal-rich (metals are elements heavier than helium) and ‘H’ for magnetic. About 80–85% of the WD population are DA WDs [47,48]. Non-DA WDs probably lost their hydrogen in a relatively late-occurring shell flash.

*White dwarf composition.* The composition of the WD core is some combination of carbon (from the burning of helium), oxygen (from the burning of carbon) and rarely neon (from the burning of oxygen). The vast majority of WD cores contain carbon and oxygen because they are not hot enough to host copious quantities of oxygen and neon. Only trace amounts of other metals should exist.

*White dwarf mass.* The initial mass function combined with the current age of the Galaxy has conspired to yield a present-day distribution of WD masses according to fig. 2 of [47] and figs 8, 10 and 11 of [55]. These figures indicate a unimodal distribution that is peaked at about 0.6 *M*_⊙_ and contains a long tail at masses higher than 0.8 *M*_⊙_. This distribution also conforms with a previous large (348 objects) survey [56], where 0.4 *M*_⊙_ and 0.8 *M*_⊙_ values are considered to be ‘low-mass’ and ‘high-mass’ [57]. In principle, WD masses can range up to about 1.4 *M*_⊙_. Only single stars with $M_{⋆}^{\left(\mathrm{MS}\right)}≳0.8\;M_{⊙}$ could have already become WDs, and hence single WDs must have masses that satisfy $M_{⋆}^{\left(\mathrm{WD}\right)}≳0.4\;M_{⊙}$. For comparable or lower mass single WDs, perhaps substellar companions could have stripped away some of this mass during the CE phase [58].

How the mass of a WD is related to its progenitor MS mass represents an extensive field of study characterized by the ‘initial-to-final-mass relation’. Observationally, this relation is often determined with WDs that are members of stellar clusters whose ages are well constrained. However, the relation is dependent on stellar metallicity, and in particular the chemistry of individual stars. Ignoring those dependencies, some relations used in the post-MS planetary literature include eqn 6 of [59] (originally from [60]), eqn 9 of [61] (originally from [21]) and eqn 6 of [62] (originally from [63]).

One study which did evaluate how the initial–final mass relationship is a function of metallicity is Meng *et al.* [64]. They found that metallicity can change the final mass by 0.4 *M*_⊙_, a potentially alarming variation given the difference between a ‘low-mass’ WD (0.4 *M*_⊙_) and a ‘high-mass’ WD (0.8 *M*_⊙_). Meng *et al.* [64] also provided in their appendix potentially useful WD–MS mass relations as a function of metallicity, for *Z*_★_=[0.0001,0.0003,0.001,0.004,0.01,0.02,0.03,0.04,0.05,0.06,0.08,0.1]. For Solar metallicity (*Z*_★_=*Z*_⊙_=0.02) and any star that will become a WD for $0.8\;M_{⊙}<M_{⋆}^{\left(\mathrm{MS}\right)}<6.0\;M_{⊙}$, they found

2.3 $$\begin{array}{rl} \frac{M_{⋆}^{\left(\mathrm{WD}\right)}}{M_{⊙}} & =\min\left[0.572-0.046\frac{M_{⋆}^{\left(\mathrm{MS}\right)}}{M_{⊙}}+0.0288{\left(\frac{M_{⋆}^{\left(\mathrm{MS}\right)}}{M_{⊙}}\right)}^{2},1.153-0.242\frac{M_{⋆}^{\left(\mathrm{MS}\right)}}{M_{⊙}}+0.0409{\left(\frac{M_{⋆}^{\left(\mathrm{MS}\right)}}{M_{⊙}}\right)}^{2}\right]. \end{array}$$

*White dwarf radius.* Usefully for modellers, the radius of the WD can be estimated entirely in terms of $M_{⋆}^{\left(\mathrm{WD}\right)}$ with explicit formulae. A particularly compact but broad approximation is $R_{⋆}^{\left(\mathrm{WD}\right)}/R_{⊙}∼10^{-2}{\left(M_{⋆}^{\left(\mathrm{WD}\right)}/M_{⊙}\right)}^{-1/3}$. Alternatively, more accurate formulae—which are derived assuming that the WD temperature is zero—that are within a few percent of one another are from eqn 15 of [65], and, as shown below, eqns 27–28 of [66]:

2.4 $$\frac{R_{⋆}^{\left(\mathrm{WD}\right)}}{R_{⊙}}\approx 0.0127{\left(\frac{M_{⋆}^{\left(\mathrm{WD}\right)}}{M_{⊙}}\right)}^{-1/3}\sqrt{1-0.607{\left(\frac{M_{⋆}^{\left(\mathrm{WD}\right)}}{M_{⊙}}\right)}^{4/3}}.$$

From equation (2.4), I obtain a canonical WD radius of 8750 km=0.0126*R*_⊙_, assuming the fiducial value of $M_{⋆}^{(\mathrm{WD})}=0.6\;M_{⊙}$.

*White dwarf luminosity.* The luminosity of WDs can be estimated in multiple ways. A rough approximation that does not include dependencies on stellar mass or metallicity is from eqn 8 of [67], which is originally from Althaus *et al.* [68]: *L*_★_=*L*(*t*_cool_=0)×[*t*_cool_/10^5^ years]^−1.25^, where *t*_cool_ is the WD cooling age. I include these dependencies by combining the prescription originally from Mestel [69] with expressions used in post-MS planetary contexts from eqn 6 of [32] and eqn 5 of [70] to obtain

2.5 $$L_{⋆}^{\left(\mathrm{WD}\right)}=3.26L_{⊙}\left(\frac{M_{⋆}^{\left(\mathrm{WD}\right)}}{0.6\;M_{⊙}}\right){\left(\frac{Z_{⋆}}{0.02}\right)}^{0.4}{\left(0.1+\frac{t_{\mathrm{cool}}}{\mathrm{Myr}}\right)}^{-1.18},$$

where *Z*_★_ is the assumed-to-be-fixed stellar metallicity. Depending on the WD cooling age, the star’s luminosity can range from about 10^3^*L*_⊙_ to 10^−5^*L*_⊙_. This formula also applies only until ‘crystallization’ sets in, which occurs for $T_{⋆}^{\left(\mathrm{WD}\right)}≲6000$–8000 K [71].

#### 2.1.4 Neutron stars

For stars with $M_{⋆}^{(\mathrm{MS})}≳8\;M_{⊙}$, the end of the AGB phase results in an explosion: a core collapse plus an outwardly expanding shockwave that nearly instantaneously (with velocities of approx. 10^3^−10^4^ km s^−1^) expels the envelope and causes the star to lose at least half of its mass. This event is a supernova (SN). Any asymmetry in the SN will cause a velocity ‘kick’. The remaining stellar core becomes either a neutron star (NS) or a black hole. Of most relevance to post-MS planetary science are pulsars, which are an august class of NSs that represent precise, stable and reliable clocks.

Although NSs and WDs are together grouped as ‘compact stars’, NSs are much more compact, with radii on the order of 10 km. NS masses are greater than those of WD stars. Typically $M_{⋆}^{\left(\mathrm{NS}\right)}\geq 1.4\;M_{⊙}$. NSs cool much faster than WDs, with a decreasing luminosity which can be modelled by (p. 30 of [72])

2.6 $$L_{⋆}^{\left(\mathrm{NS}\right)}=0.02L_{⊙}{\left(\frac{M_{⋆}^{\left(\mathrm{NS}\right)}}{M_{⊙}}\right)}^{2/3}\left[\frac{\max(t_{\mathrm{cool}},0.1\;\mathrm{Myr})}{\mathrm{Myr}}\right],$$

where *t*_cool_ represents the NS cooling time in this context.

Millisecond pulsars have rotational periods on the order of milliseconds. They are thought to have been spun up by accretion, and are hence said to be ‘recycled’. Miller & Hamilton [73] argued that the presence of planets around millisecond pulsars can constrain the evolutionary history of the star. In particular, they posed that the moon-sized SB around the millisecond pulsar PSR B1257+12 demonstrates how that particular star is not recycled by (i) favouring a second-generation formation scenario for the SB (see §8) and (ii) suggesting that the formation cannot have occurred during an accretional event nor in a post-spin-up disc. They claimed that the moon-sized SB must have formed, post-SN, around the star as is with its current rotational frequency and magnetic moment.

### 2.2 Common envelope and binary star evolution

Stellar binary systems are important because they represent several tens of percent of all Milky Way stellar systems. The presence of a stellar binary companion can significantly complicate the evolution if the mutual separation is within a few tens of AU. Both star–star tides and the formation of a ‘common envelope’ (CE) can alter the fate otherwise predicted from single-star evolution. Ivanova *et al.* [74] reviewed the theoretical work performed on and the physical understanding of CEs; see their fig. 1 for some illustrative evolutionary track examples. Taam & Ricker [75] provided a shorter, simulation-based review of the topic.

A CE is a collection of mass that envelopes either (i) two stars or (ii) one star and one large SB like a giant planet. In both cases, as the smaller binary component spirals into the larger one, the former transfers energy to the envelope. The transfer efficiency is a major unknown in the theory of stellar evolution. The smaller companion may blow off the CE by depositing a sufficient amount of energy in the envelope during inspiral. Relevant equations describing this process include eqn 17 of [76], eqns 2–5 of [77] and eqn 8 of [78]. A more complete treatment that takes into account shock propagation and rotation may be found in eqns 6–25 of [77]; also see the earlier work by [79]. The more massive the companion, and the more extended the envelope, the more likely ejection will occur. The speed of infall within the CE may be expressed generally as a quartic equation in terms of the radial velocity (eqn 9 of [51]), but only if the SB’s tangential velocity is known, as well as the accretion rate onto the SB. Eqn 1 of [58] approximates the final post-CE separation after inspiral.

Even without a CE, the interaction between both stars might dynamically excite any SBs in that system, particularly when one or both stars leave the MS. Both stars might be similar enough in age (and hence MS mass) to undergo coupled GB mass loss. Section 5.2 of [80] quantified this possibility, and finds that the MS masses of both components must roughly lie within 10% of one another in order for both to simultaneously lose mass during their AGB phases.

## 3. Observational motivation

Post-MS planetary systems provide multiple insights that are not available from MS planetary systems, including: (i) substantive access to surface and interior SB chemistry, (ii) a way to link SB fate and formation, (iii) different constraints on tidal, mass-losing and radiative processes and (iv) the environments to allow for detections of extreme SBs. The agents for all this insight come from GB, WD and NS planetary systems. Overall, the total number of WD remnant planetary systems is of the same order (approx. 1000) as MS planetary systems, and about one order of magnitude more than GB planetary systems. The number of remnant planetary systems around NSs is a few.

### 3.1 Planetary remnants in and around white dwarfs

Fragments and constituents of disrupted SBs that were planets, asteroids, moons, comets, boulders and pebbles observationally manifest themselves in the atmospheres of WDs and the debris discs which surround WDs. The mounting evidence for and growing importance of both topics is, respectively, highlighted in recent reviews [17,18]. Here, I devote just one subsection to the observational aspects of each topic.

#### 3.1.1 White dwarf atmospheric pollution

Because WDs are roughly the size of the Earth but contain approximately the mass of the Sun, WDs are about 10^5^ as dense as the Sun. Consequently, due to gravitational settling, WD atmospheres quickly separate light elements from heavy elements [81], causing the latter to sink as oil would in water. This stratification of WD atmospheres by atomic weight provides a tabula rasa upon which any ingested contaminants conspicuously stand out—as long as we detect them before they sink.

*Composition of intrinsic white dwarf photosphere.* The chemical composition of the atmosphere is dependent on (i) how the WD evolved from the GB phase and (ii) the WD’s cooling age. At the end of the AGB phase, the star’s photosphere becomes either hydrogen-rich (DA), helium-rich (DB) or a mixed hydrogen–helium composition (DAB, DBA). The link between spectral type and composition is sometimes not so clear, as more literally a DA WD refers to a WD whose strongest absorption features arise from H, and similarly a DB WD has the strongest absorption features arising from He. If the cooling age is within a few tens of Myr, then the WD is hot enough to still contain heavy elements in the photosphere. These elements are said to be ‘radiatively levitated’. For cooling ages between tens of Myr and about 500 Myr, the atmosphere consists of hydrogen and/or helium only. For cooling ages greater than 500 Myr, some carbon—but usually only carbon—from the core may be dredged up onto the atmosphere. Effectively then, WDs that are older than a few tens of Myr and do not accrete anything have atmospheres which are composed of some combination of hydrogen, helium and carbon only.

*Composition of polluted white dwarf photosphere.* Yet, we have now detected a total of 18 metals heavier than carbon in WDs with $30\;\mathrm{Myr}≲t_{\mathrm{cool}}≲500\;\mathrm{Myr}$. These metals, which are said to ‘pollute’ the WD (thereby adding a ‘Z’ designation to its spectral class), are, with atomic number, O(8), Na(11), Mg(12), Al(13), Si(14), P(15), S(16), Ca(20), Sc(21), Ti(22), V(23), Cr(24), Mn(25), Fe(26), Co(27), Ni(28), Cu(29) and Sr(38). Although N(7) has not been directly detected, there are published upper limits for that chemical element. These metals include rock-forming elements (Si, Fe, Mg, O), refractory lithophiles (Ca, Al, Ti), volatile elements (C, N, P, S) and siderophiles (Cr, Mn, S, Ni). The first polluted WD (Van Maanen 2, or vMa 2), discovered in the late 1910s, contains observable Ca, which happens to be the strongest signature in WD spectra (Mg is next) [3,4]. Only about 90 years later, with the availability of high-resolution spectroscopy from the Hubble Space Telescope, plus ground-based observations with Keck, VLT, HST and SDSS, did the floodgates open with the detection of 17 of the above metals all within the same WD (GD 362) [82]. A steady stream of highly metal-polluted WDs has now revealed unique, detailed and exquisite chemical signatures (e.g. [83–89]). Two notable cases [90,91] include a high-enough level of oxygen to indicate that the origin of the pollution consisted of water, a possibility envisaged by e.g. [92].

**Figure 4.**
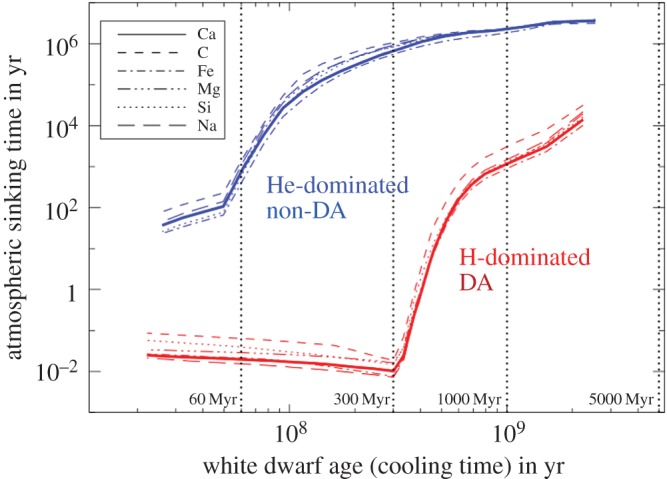
Cosmetically enhanced version of fig. 1 of [98]. Shown are the sinking times of six metals in WD atmospheres. These times are orders of magnitude less than the WD cooling ages. The sinking timescales of DA WDs younger than about 300 Myr are days to weeks.

*Planetary origin of pollution.* A common explanation for the presence of all these metals is accretion of remnant planetary material. The now-overwhelming evidence includes: (i) the presence of accompanying debris discs (see §3.1.2), (ii) SBs caught in the act of disintegrating around a polluted WD (see §3.2.1), (iii) chemical abundances that resemble the bulk Earth to zeroth order (see e.g. [17]), (iv) a variety of chemical signatures that are comparable to the diversity seen across Solar system meteorite families (see e.g. [17]), (v) the debunking of the possibility of accretion from the interstellar medium (see, e.g. eqns 2–6 and table 3 of [93]) and (vi) the fraction of polluted WD systems, which is 25–50% [94–96] and hence roughly commensurate with estimates of Milky Way MS planet-hosting systems [97]. This last point is particularly remarkable because metals heavier than carbon will sink (or ‘diffuse’, ‘settle’ or ‘sediment’) through the convection or ‘mixing’ zone quickly (figure 4): in days or weeks for DA WDs younger than about 300 Myr and within Myr for DB WDs. In all cases, the sinking times are orders of magnitude shorter than the WD cooling age. Therefore, we should always expect to detect heavy metal pollution at a level well-under 0.1%.

**Figure 5.**
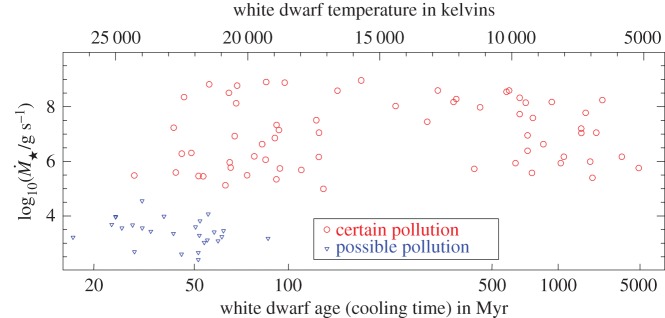
Cosmetically reconstructed version of the top panel of fig. 8 of [96]. The blue downward triangles refer to upper limits. The plot illustrates that accretion rate appears to be a flat function of WD cooling age: pollution occurs at similar rates for young and old WDs.

Because the percentage is actually 25–50%, then for most DA WDs (which represent about 80% of all WDs [55]) the accretion is ongoing right now. The accretion occurs at similar levels along all detectable WD cooling ages (up to approx. 5 Gyr; figure 5), highlighting an important challenge for theorists: what planetary architectures can generate comparably high levels of accretion at such late ages? (The first polluted WD, vMa 2, is relatively ‘old’, with *t*_cool_=3 Gyr.)

*Implications for planetary chemistry and surfaces/interiors.* For the foreseeable future, the only reliable way to study the chemistry of SBs will be through spectroscopic observations of their tidally disrupted remnants in WD atmospheres. Samples from Solar system meteorites, comets and planets (including the Earth) allow us to make direct connections to chemical element distributions in WD atmospheres. For example, we know that an overabundance of S, Cr, Fe and Ni indicates melting and perhaps differentiation [83]. Signatures of core and crust formation are imbued in the ratio of iron to siderophiles or refractory lithophiles. Also, in particular, Fe-rich cores, Fe-poor mantles or Al-rich crusts may all be distinguished [99]. A carbon-to-oxygen ratio ≳0.8 would result in drastically different physical setup than the Solar system’s [100]. For more details, see [17].

*Implications for planetary statistics.* Because polluted WDs signify planetary systems, these stars can be used to probe characteristics of the Galactic exoplanet population. Zuckerman [101] considered the population of polluted WDs which are in wide binary systems, and concluded that a comparable fraction of both single-star and wide binary-star systems with $r_{b}≳1000\;\mathrm{AU}$ host planets. For $r_{b}≲1000\;\mathrm{AU}$, however, the binary planet-hosting fraction is less, implying perhaps that in these cases the binary companion suppresses planet formation or more easily creates dynamical instability.

**Figure 6.**
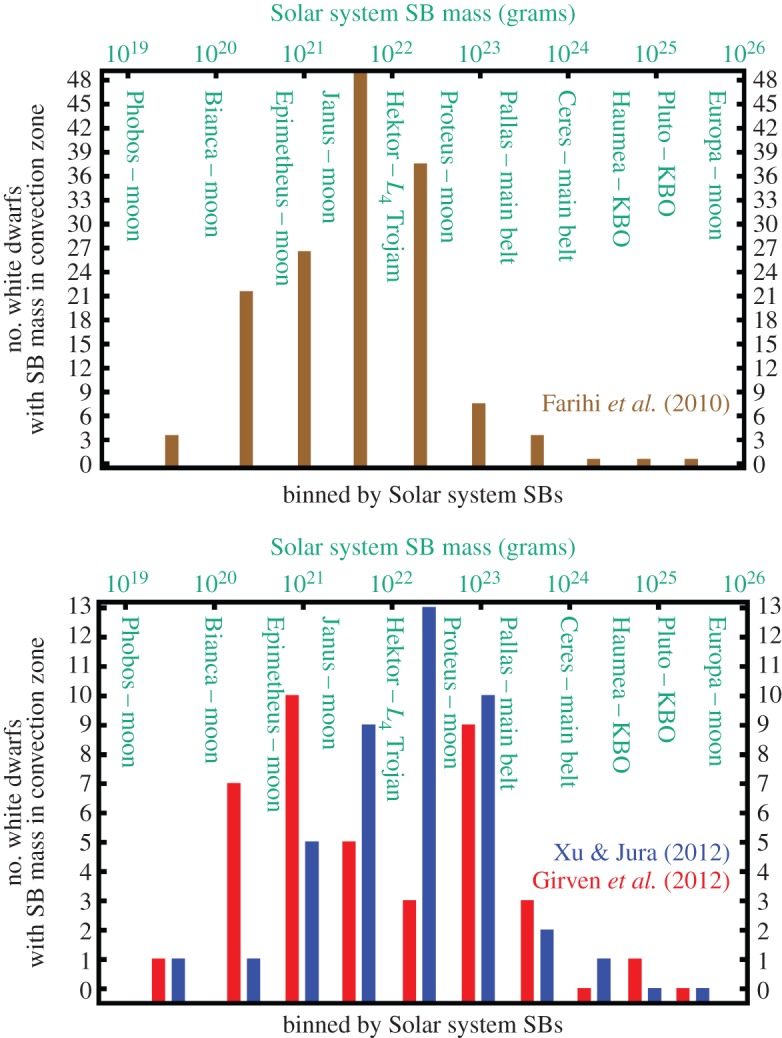
Histograms of the accumulated mass of rocky substellar bodies that were accreted onto white dwarfs during the last Myr or so, including both detections and limiting values. Differently coloured bars refer to three different WD samples (brown: data from [93] assuming that Ca represents 1.6% of the mass of the accreted bodies, similar to the corresponding mass fraction of the bulk Earth—see table 3 of [102]; blue: data displayed in fig. 12 of [103]; red: data displayed in fig. 9 of [104]). The panels are separated according to sample size (see *y*-axis). For observational subtleties associated with the data, see the corresponding papers. The bin sizes are according to the Solar system objects displayed in green, with masses given on the top axis. This plot demonstrates that pollution may arise from a wide variety of objects.

*Accumulated metal mass in the convection zone.* For DB WDs, the situation is different. Their convection zones are deep enough to hold a record of all remnant planetary accretion over the last Myr or so. This feature allows us to estimate lower bounds for the total amount accreted over this timescale. Figure 6 illustrates the amount of mass in metals, in terms of Solar system asteroids, moons and one L_4_ Jupiter Trojan, that has been accreted in two different WD samples.

*Other constraints.* Some metal-polluted WDs are magnetic (denoted with an ‘H’ in the spectral classification). At least 10 DZH WDs harbour magnetic fields of 0.5–10 MG [105], although this preliminary work indicates this number is likely to be double or triple. The theoretical implications of magnetic fields have previously only briefly been touched upon (§12.2).

Further, hydrogen abundance in WD atmospheres, although not considered a ‘pollutant’, nevertheless might provide an important constraint on pollution. Because hydrogen does not diffuse out of a WD atmosphere, this chemical element represents a permanent historical tracer of accretion throughout the WD lifetime (even if the WD’s spectral type changes as a function of time). Accretion analyses and interpretations, however, must assume that the WD begins life with a certain amount of primordial H. This accretion arises from a combination of the interstellar medium, asteroids, comets and any planets. Of these, comets—and in particular exo-Oort cloud comets—might provide the greatest amount of this hydrogen through ices. Consequently, linking WD hydrogen content with cooling age may help determine the accretion rate of exo-Oort cloud comets soon after the WD is formed [67] and over time [106]. Fig. 5 of [91] illustrates how WD hydrogen mass appears to be a steadily increasing function of cooling age, and increases at a rate far greater than realistic estimates of accretion from the interstellar medium.

#### 3.1.2 White dwarf debris discs

Debris discs have been detected orbiting nearly 40 polluted WDs. The first disc discovered orbits the WD Giclas 29–38 (commonly known as G 29–38) [107] in 1987. Nearly two decades passed before the second disc, orbiting GD 362 [108,109], prompted rapid progress. No confidently reported debris disc around a single unpolluted WD exists, suggesting the link between pollution and discs is strong. At least a few percent and up to 100% of all WDs host discs [110–112]. The lower limit for the Galactic population is based on actually observed discs, whereas the one-to-one potential correspondence between pollution and the presence of a disc is based on most discs likely being too faint to detect. Although observational sensitivities allow pollution to be discovered in WDs with *t*_cool_ as high as about 5 Gyr, discs are difficult to detect for *t*_cool_>0.5 Gyr [112]. Farihi [18] recently summarized observations of these discs. See also table 1 of [110], table 1 of [103] and table 2 of [113] for some details of dust-only discs found before 2012.

*Detection constraints.* All these discs are dusty, and dust comprises the major if not sole component. Consequently, the detection and characterization of the discs rely on modelling spectral energy distributions with a signature (‘excess’ with respect to the flux from the WD) in the infrared and a total flux, F, prescription that is given in eqn 3 of [114]:

3.1 $$F\approx 12\pi^{1/3}\cos\left(i_{\mathrm{LOS}}\right)\frac{R_{⋆}^{2}}{D^{2}}{\left(\frac{2k_{B}T_{⋆}}{3h\nu }\right)}^{8/3}\frac{h\nu^{3}}{c^{2}}\int_{x^{\left(\mathrm{in}\right)}}^{x^{\left(\mathrm{out}\right)}}\frac{x^{5/3}}{\exp(x)-1}\;dx.$$

In equation (3.1), *ν* is the frequency, *D* is the distance between the star and the Earth, *i*_LOS_ is the line-of-sight inclination with respect to the Earth, *k*_B_ is the Boltzmann constant, *h* is the Planck constant and *x*(*r*)≡*hν*/*k*_B_*T*_d_(*r*). The discs are assumed to be passive, opaque (optically thick) and geometrically flat.

The equation is degenerate with respect to three parameters: *i*_LOS_, and the disc temperatures at the inner and outer edges. Fig. 5 of [104] and fig. 3 of [115] illustrate how the degeneracy from these three parameters manifests itself in the modelling of debris discs. For an explicit example of how 10 different viewing angles can change the flux signature, see fig. 1 of Livio *et al.* [57], who simulate the spectral energy distribution for a (so-far unrealized) AU-scale WD debris disc.

*Disc characteristics.* One such property is disc geometry. The spectral energy distributions generally do not indicate that WD discs are flared. However, a couple of possible exceptions include GD 362 [116] and GD 56 [117]. The size distribution of the dust/particles/solids in the discs is unknown, except for the presence of micrometre-sized grains [118–120]. One notable disc, which orbits WD J0959-0200, is highly variable: Xu & Jura [115] reported a still-unexplained flux decrease of about 35% in under 300 days.

Application of equation (3.1), with assumptions about *i*_LOS_, yields a striking result for WDs with *t*_cool_ greater than a few Myr: the entire disc lies within the Roche radius of the WD. Fits from this equation suggest that the discs reside in the region *r*_d_≈0.6−1.2 *R*_⊙_. Confirmation of this approximate range arose with the discovery of both dusty and gaseous components in seven of these discs. The gaseous components constrain the disc geometry. This distance range clearly demonstrates that (i) the discs do not extend all the way to the WD surface (photosphere) and that (ii) the discs could not have formed during the MS or GB phases. Regarding this first point, some spectral features do suggest the presence of gas within 0.6*R*_⊙_ (e.g. see the bottom-left panel of fig. 3 in [83]), but not yet in a disc form.

**Figure 7.**
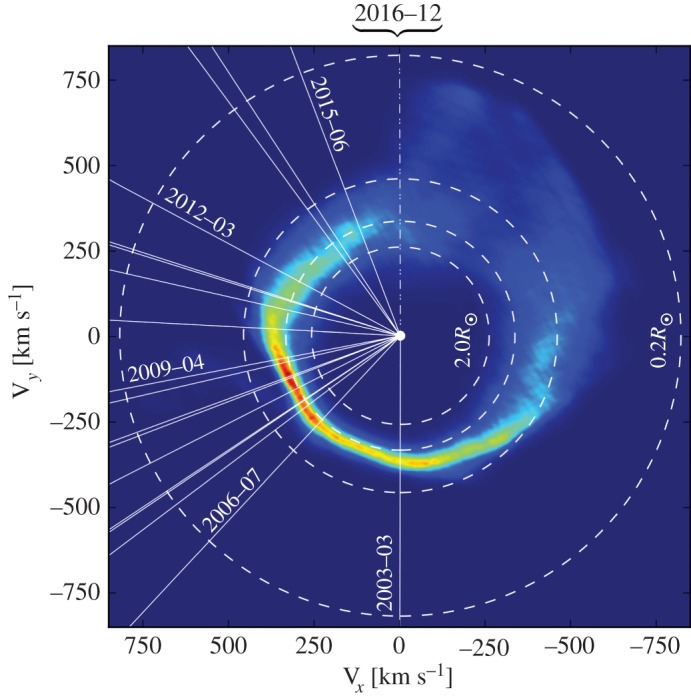
Exact reproduction of fig. 5 of [122]. This image is a velocity space map of the gaseous component of the debris disc orbiting the WD SDSS J1228+1040. The subscripts *x* and *y* refer to their usual Cartesian meanings, and the WD is located at the origin. Observations at particular dates are indicated by solid white lines. The image suggests that the disc is highly non-axisymmetric and precessing on decadal timescales.

The first gaseous disc component found (around SDSS J122859.93+104032.9, also known as SDSS J1228+1040) [121], also exhibits striking morphological changes, which occur secularly and smoothly over decades (whereas the disc orbital period is just a few hours) [122] (figure 7). The figure is a velocity space intensity distribution where the radial white lines indicate different times from the years 2003–2016. Four other discs with time-resolved observations of gaseous components are SDSS J0845+2258, SDSS J1043+0855, SDSS J1617+1620 and SDSS J0738+1835. The first three of these—which change shape or flux over yearly and decadal timescales—represent exciting dynamical objects, while the last, which exists in an apparently steady state (given just a handful of epochs so far), might provide an important and intriguing contrast.

One notable exception to all of the above WD discs is a very wide (35–150 AU) dusty structure inferred orbiting the extremely young (*t*_cool_≪1 Myr) WD 2226-210 [123]. The interpretation of this dusty annulus representing a remnant exo-Kuiper belt is degenerate and is not favoured compared to a stellar origin [124]: i.e. this annulus might represent a planetary nebula.

### 3.2 Major and minor planets around white dwarfs

#### 3.2.1 Orbiting white dwarfs

A few WDs host orbiting SBs, and they are all exoplanetary record-breakers (as of time of writing) in at least one way.

*The fastest, closest and smallest SBs.* Transit photometry of WD 1145+017 revealed signatures of one to several SBs (with *R*_SB_<10^3^ km) which are currently disintegrating within the WD disruption radius with orbital periods of 4.5–5.0 h [125,126]. Gänsicke [127] have since constrained the orbital periods of at least six SBs to within 15 s of 4.4930 h each, indicating almost exactly coplanar and equal orbits. Further, within the same system, Xu *et al.* [128] have detected circumstellar absorption lines from likely gas streams, as well as 11 different metals in the WD atmosphere.

Because this WD is both polluted and hosts a dusty debris disc, these minor planet(s) further confirm the interpretation that accretion onto WDs and the presence of circumstellar discs is linked to first-generation SB disruption (see §9). This type of discovery was foreshadowed by previous prognostications. (i) Soker [129] found that for stars transitioning from the AGB to WD phase, their shocked winds can create mass ablation from surviving planets into a detectable debris tail. (ii) Villaver & Livio [52] predicted that planets evaporating and emitting Parker winds could be detected with spectroscopic observations, but was thinking of atmospheric mass outflows at several AU around GB stars. (iii) Di Stefano *et al.* [130] demonstrated specifically that the *Kepler* space mission should be able to detect WD transits of minor planets. Ironically, although the paper was written with the primary *Kepler* mission in mind, only during the secondary mission were enough WDs observed to achieve this discovery. (iv) Alternatively, Spiegel & Madhusudhan [131] claimed that the process of a stellar wind accreting onto an SB might produce a detectable coronal envelope around the SB.

*The furthest and slowest exoplanet.* WD 0806-661 b is a planetary mass (7*M*_Jup_) SB orbiting the WD at an approximate distance of 2500 AU [132]. Although some in the literature refer to the object as a brown dwarf, the mass is well constrained to be in the planet regime (see fig. 4 of [132]). The difference of opinions is perhaps partly informed by contrasting assumptions about the SB’s dynamical origin rather than its physical properties. The planet was discovered using direct imaging, and holds the current record for the bound exoplanet with the widest orbit known.

*The first circumbinary exoplanet.* The first successfully predicted [133,134] and confirmed [135] circumbinary exoplanet, PSR B1620-26AB b, orbits both a WD (with mass ≈0.34 *M*_⊙_ and cooling age of approx. 480 Myr) and a millisecond pulsar (with mass ≈1.35 *M*_⊙_ and rotation period of 11 ms). The WD cooling age and pulsar rotation period importantly help constrain the dynamical history of the system. The planet’s physical and orbital parameters are *M*_SB_∼2.5*M*_Jup_, *a*∼23 AU and *i*∼40°, whereas the binary orbital parameters are *a*_b_≈0.8 AU and *e*_b_≈0.025.

PSR B1620-26AB b is the only known planet in a system with two post-MS stars, and one of the few exoplanets ever observed in a metal-poor environment and cluster environment (the M4 globular cluster). The planet name contains ‘PSR’ because the pulsar was the first object in the system discovered and is the most massive object (the primary). However, the planet was originally thought to orbit (and form around) the progenitor of the WD, and hence is more appropriately linked to that star. Further, I do not classify this system as a post-CE binary (see §3.4) because both the system does not fit the definition of containing a WD and a lower-mass MS companion, and the system is typically not included in the post-CE binary literature [136]. This combination of pulsar, WD and planet suggests a particularly fascinating dynamical history (see §7.3.2).

*Hints of detections.* In addition to the above observations, there are several hints of SBs orbiting WDs. The magnetic WD GD 365 exhibits emission lines which could indicate the presence of a rocky planet with a conducting composition [137]. Later data has been able to rule out an SB with *M*_SB_≥12*M*_Jup_ [25], which is consistent with the rocky planet hypothesis. Also, the spectral energy distribution of PG 0010+280 may be fit with an SB with *r*≈60 AU [138]. In order for this SB to be hot enough for detection, it may have been re-heated; see their Sec. ii.

Further, a few tenths of percent of Milky Way WDs host brown dwarf-mass SBs [139]. These companions were found to orbit at distances beyond the tidal engulfment radius of the AGB progenitor of the WD until the notable discovery of WD 0137-349 B [140]. This brown dwarf has *M*_SB_=0.053 *M*_⊙_. For the primary WD star, *M*_★_=0.39 *M*_⊙_. The orbit is close enough—($a\sin i_{\mathrm{LOS}}=0.375R_{⊙}=0.0017\;\mathrm{AU}$)—that WD 0137-349 B must have survived engulfment in the GB envelope of the progenitor. The low mass of the WD is characteristic of premature CE ejection by a companion.

*Search methods.* For a recent short summary of the different techniques employed to search for planets around WDs, see Section 1 of [141]. The discovery of SBs orbiting WD 1145+017 based on transit photometry [125] highlights interest in this technique. Faedi *et al.* [142] placed limits (less than 10%) on the frequency of gas giants or brown dwarfs on circular orbits with orbital periods of several hours, and mentioned that exo-moons orbiting WDs can generate 3% transit depths. An important caveat to this transit method is that it requires follow-up with other methods, at least according to [143], who wrote that for WDs, ‘planet detection based on photometry alone would not be credible’.

Existing relevant formulae suppose that the SB is smaller than the star (which is not true for giant planets or Earth-sized SBs orbiting WDs), and some formulae make other approximations (like circularity of orbits) which would be ill-suited for the type of transits suggested by e.g. [144]. Useful formulae from post-MS studies are provided by eqns 1–2 of [142], eqn 1 of [145] and eqns 18–19 of [76]. Faedi *et al.* [142] estimate the depth of the transit to be equal to unity if *R*_SB_≥*R*_★_ and, instead, to approximately equal $R_{\mathrm{SB}}^{2}/R_{⋆}^{2}$ if *R*_SB_<*R*_★_. The probability of transit and the duration of transit represent other quantities of interest. I display these by repackaging the fairly general expressions from eqns 9–10 of [146]:

3.2 $$\mathrm{probability}=\left(\frac{R_{⋆}+R_{\mathrm{SB}}}{a}\right)\left(\frac{1\pm e\sin\omega }{1-e^{2}}\right),$$

where *ω* is the argument of pericentre of the orbit, the upper sign is for transits (SB passing in front of the WD) and the lower sign is for occultations (or ‘secondary eclipses’, when the SB passes in back of the WD). Both formulae assume grazing eclipses, and that eclipses are centred around conjunctions. Maintaining this sign convention, I then combine eqns 7, 8, 14, 15 and 16 of [146] to obtain the transit/occultation duration:

3.3 $$\begin{array}{rl} \mathrm{duration} & =2\left(\frac{\sqrt{1-e^{2}}}{1+e\sin\omega }\right)\sqrt{\frac{a^{3}}{G(M_{⋆}+M_{\mathrm{SB}})}}\\ & \;\times \sin^{-1}\left[\frac{R_{⋆}}{a\sin i_{\mathrm{LOS}}}{\left\{{\left(1\pm \frac{R_{\mathrm{SB}}}{R_{⋆}}\right)}^{2}-\frac{a^{2}\cos^{2}i_{\mathrm{LOS}}{\left(1-e^{2}\right)}^{2}}{R_{⋆}^{2}{\left(1+e\sin\omega \right)}^{2}}\right\}}^{1/2}\right], \end{array}$$

where *i*_LOS_ is the inclination of the orbit with respect to the line of sight of an observer on the Earth. By convention, an edge-on orientation corresponds to *i*_LOS_=90°.

#### 3.2.2 Orbiting the companions of white dwarfs

Three stellar systems are known to harbour a planet-hosting star and a WD: GJ 86, *ϵ* Reticulum (or *ϵ* Ret), and HD 147513. In no case is the WD (yet) known to be polluted nor host debris discs, planets or asteroids.

The star GJ 86B is an unpolluted WD [147] whose binary companion GJ 86A is an MS star that hosts a $M_{\mathrm{SB}}≳4.5M_{\mathrm{Jup}}$ planet in an *a*=0.11 AU orbit [148,149]. With Hubble Space Telescope data, Farihi *et al.* [147] helped constrain the physical and orbital parameters of the system (see their table 4), which features a current binary separation of many tens of AU. The planet GJ 86Ab survived the GB evolution of GJ 86B, when *a*_b_ expanded by a factor of a few, but *e*_b_ remained fixed (see §4).

A similar scenario holds for the HD 27442 system. The star *ϵ* Ret, or HD 27442A, hosts a $M_{\mathrm{SB}}≳1.6M_{\mathrm{Jup}}$ planet in a *a*=1.27 orbit [150]. The projected separation between *ϵ* Ret and its WD binary companion, HD 27442B, is approximately a couple hundred AU [151]. The HD 147513 system is not as well constrained [152]. However, the separation between the binary stars in that system is thought to be several thousand AU, placing it in the interesting ‘non-adiabatic’ mass loss evolutionary regime (equation (4.10)).

#### 3.2.3 White dwarf–comet collisions

In the 1980s, the potential for collisions between exo-comets and their parent stars, or other stars, to produce observable signatures was realized [153–155]. Alcock *et al.* [156] more specifically suggested that comet accretion onto WDs can constrain number of exo-Oort cloud comets in other systems. Pineault & Poisson [155] and Tremaine [157] included detailed analytics that may still be applicable today. Some of this analysis was extended to binary star systems in Pineault & Duquet [158], with specific application to compact objects in their Sec. 4.3. Perhaps, these speculations have been realized with the mysterious X-ray signature from IGR J17361-4441 reported in Del Santo *et al.* [159], although that potential disruption event may also have just been caused by planets or asteroids rather than comets.

### 3.3 Subgiant and giant star planetary systems

#### 3.3.1 Giant branch planets

*Gross characteristics.* As of 30 Nov 2015, 79 SBs were recorded in the planets-around-GB-stars database^1^ , although this number may be closer to 100 [160]. About 85% of these SBs are giant planets with *M*_SB_∼1−−13*M*_Jup_, proving that planets can survive over the entire MS lifetime of their parent stars. The host stars for these SBs have not undergone enough GB evolution to incite mass loss or radius variations which are markedly different from their progenitor stars. These barely evolved host stars are observed in their early RGB phase, sometimes known as the ‘subgiant’ phase. Because RGB tracks on the Hertzprung–Russell diagram are so close to one another, RGB masses are hard to isolate; there is an ongoing debate over the subgiant SB-host masses [161–166].

Regardless, the population of these SBs shows a distinct characteristic: a paucity of planets (less than 10%) within half of an AU of their parent star. In contrast, *a*<0.5 AU holds true for about three-quarters of all known exoplanets. This difference highlights the need to better understand the long-term evolution of planetary systems. Further, a handful of GB stars have been observed to host multiple planets. These systems may reveal important constraints on dynamical history. For example, the two planets orbiting *η* Ceti may be trapped in a 2:1 mean motion resonance (see the fourth and fifth rows of plots in fig. 6 of [167]). A resonant configuration during the GB phase would help confirm the stabilizing nature of (at least some types of) resonances throughout all of MS evolution.

Regarding the planet–giant star metallicity correlation, Maldonado *et al.* [168] and Reffert *et al.* [169] arrived at somewhat different conclusions. The former concluded that planet-hosting giant stars are preferentially not metal rich compared to giant stars that do not host planets. The latter, using a different sample, showed that there was strong evidence for a planet—giant star metallicity correlation.

*Lithium pollution?* One case of particular interest is the BD+48 740 system [37], which contains both (i) a candidate planet with an unusually high planet eccentricity (*e*=0.67) compared with other evolved star planets and (ii) a host star that is overabundant in lithium. Taken together, these features are suggestive of recent planet engulfment: Lillo-Box *et al.* [10] wrote: ‘The first clear evidence of planet engulfment’. They also helped confirm existence of the MS planet Kepler-91b, a tidally-inspiraling extremely close-in planet with *r*≈1.32*R*_★_, and estimate that its fate might soon (within 55 Myr) mirror that of the engulfed planet in BD+48 740.

*Transit detection prospects.* In principle, SBs may be detected by transits around GB stars. The application of equations (3.2 and 3.3) to this scenario is interesting because, as observed by Assef *et al.* [170], despite the orders of magnitude increase in stellar radius from the MS to GB phases, there is a corresponding increase in values of *a* for surviving SBs. Therefore, the transit probability should not be markedly different. The equations neglect however, just how small the transit depth becomes: $R_{\mathrm{SB}}^{2}/R_{⋆}^{2}∼10^{-5}$.

#### 3.3.2 Giant branch debris discs

Importantly, planets are not the only SBs that survive MS evolution. Bonsor *et al.* [171] revealed the first resolved images of a debris disc orbiting a 2.5 Gyr-old subgiant ‘retired’ A star (*κ* Coronae Borealis or *κ* CrB), although they could not distinguish between one belt from 20 to 220 AU from two rings or narrow belts at about 40 and 165 AU. This finding demonstrates that either the structure survived for the entire main sequence lifetime, or underwent second-generation formation. This discovery was followed up with a survey of 35 other subgiant stars, three of which (HR 8461, HD 208585 and HD 131496) exhibit infrared excess thought to be debris discs [172]. Taken together, these four disc-bearing GB stars suggest that large quantities of dust could survive MS evolution.

### 3.4 Putative planets in post-common-envelope binaries

Some binary stars which have already experienced a CE phase are currently composed of (i) either a WD or hot subdwarf star, plus (ii) a lower-mass companion. These binaries are classified as either ‘detached’ or ‘semi-detached cataclysmic variables’ depending on the value of *r*_b_. Over 55 of these binaries have just the right orientation to our line of sight to eclipse one another. These systems are known as post-CE binaries. The eclipse times in these binaries should be predictable if there are no other bodies in the system and if the stars are physically static objects.

In several cases, this idealized scenario has not been realized, allowing for the exciting possibility of exoplanet detections. Zorotovic & Schreiber [136] reviewed the potential origin of eclipse timing variations for all known post-CE binaries (see their table 1 and appendix A), and emphasized that ‘extreme caution’ must be exercised when evaluating a first-generation origin for these putative planets. The reason is that binary stars are complex structures and can mimic planetary signals. Of the 12 systems highlighted in that table for potentially hosting planets or brown dwarfs, the existence of these SBs remain in doubt due to stability analyses (see §7.4). For SBs which are thought to exist, their dynamical origin remains in question. Section 5 of [136] discusses the possibility of second-generation formation; see also §8 of this paper.

The most robust detections are the putative planets around the post-CE binary NN Serpentis, or NN Ser. Beuermann *et al.* [173,174] found excellent agreement with a two-planet fit, and found resonant solutions with true librating angles [174]. A recent analysis of 25 years worth of eclipse timing data for this system (see fig. 2 of [175]) strengthens the planetary hypothesis, particularly because timings between the years of 2010–2015 matched the predicted curve. They do not, however, claim that these planets are confirmed, because there still exists a degeneracy in the orbital solutions. In fact, Mustill *et al.* [176] provided evidence against the first-generation nature of these planets by effectively backwards integrating in time to determine if the planets could have survived on the MS. The largest uncertainty in their study is not with the orbital fitting but rather how the CE evolved and blew off in that system.

If confirmed, other reported systems which may be dynamically stable would prove exciting. However, pulsation signals, which are intrinsic to the parent star, can mask timing variations that would otherwise indicate the presence of SBs (e.g. [177]). Charpinet *et al.* [178] detected signals around the hot subdwarf star KIC 05807616 which could correspond to SBs with distances of 0.0060 and 0.0076 AU. Silvotti *et al.* [179] instead detected timing variations around the subdwarf pulsator KIC 10001893 with periods of 5.3, 7.8 and 19.5 h.

### 3.5 Pulsar planets

Pulsar arrival timings in systems with a single pulsar are generally better constrained than those in post-CE binaries because pulsars are more reliable clocks than WD or MS stars, and radiation from a single source simplifies the interpretation. Identifying the origin of residual signals for millisecond pulsars is even easier.

These highly precise cosmic clocks, combined with a fortuitous spell of necessary maintenance work on the Arecibo radio telescope, provided Alexander Wolszczan with the opportunity to find PSR B1257+12 c and d [1] (sometimes known as PSR B1257+12 B and C), and eventually later PSR B1257+12 b (sometimes known as PSR B1257+12 A) [2]. Wolszczan [180] detailed the history of these discoveries. Not included in that history is how the identities of the first two planets were almost prematurely leaked by the British newspaper *The Independent* in October 1991 [181]. The article referred to these planets as ‘only the second and third planets to be found outside our own solar system’ because of the ironic assumption that the first-ever exoplanet was the (later-retracted [182]) candidate PSR 1829-10 b.

The observed and derived parameters for the PSR B1257+12 system remain among the best constrained of all exoplanets (see table 1 of [180]), with eccentricity precision to the 10^−4^ level, a value for the mutual inclination of planets c and d (which is about 6 degrees), and a derived mass for planet b of 0.020 *M*_⊕_=1.6*M*_Moon_. However, these values are based on the assumption that *M*_★_=1.4 *M*_⊙_. PSR B1257+12 b has the smallest mass of any known extrasolar SB, given that we do not yet have well-constrained masses for the (disintegrating) SBs orbiting WD 1145+017. The other two planets are ‘super-Earths’, with masses of planets c and d of 4.3*M*_⊕_ and 3.9*M*_⊕_. All three planets lie within 0.46 AU of the pulsar and travel on nearly circular (*e*<0.026) orbits. The orbital period ratio of planets b and c is about 1.4759, which is close to the 3:2 mean motion commensurability.

I have already described the one other bona-fide planet discovered orbiting a millisecond pulsar, PSR B1620-26AB b, in §3.2.1, because that planet also orbits a WD. For an alternative and expanded accounting of this planet as well as the PSR B1257+12 system, see the 2008 review of neutron star research [183].

Pulsar planets are rare. A recent search of 151 young (<2 Myr old) pulsars (not ms pulsars) with the *Fermi* telescope yielded no planets for *M*_SB_>0.4*M*_⊕_ and with orbital periods of under 1 year [184]. The authors used this result as strong evidence against post-SN fallback second-generation discs (see §8.2), particularly because theoretical models constrain these discs to reside within about 2 AU.

### 3.6 Circumpulsar asteroid and disc signatures

Sometimes the deviations in pulse timing are not clean in the sense that they cannot be fit with one or a few planets or moon-sized SBs. Rather, the deviations may be consistent with other structures, such as discs, rings, arcs or clouds. Wang [185] recently reviewed observational results from debris disc searches around single pulsars.

The residuals for the millisecond pulsar B1937+21 are consistent with an asteroid disc of mass less than about 0.05 *M*_⊕_ [186], and with constrained properties given in their table 2. Asteroids can affect the timing precision of received signals from millisecond pulsars down to the ns level. Their fig. 5 nicely compares their sensitivity limits for asteroids in PSR B1937+21 with the PSR B1257+12 planets. Unfortunately, the asteroids interpretation is degenerate with other possibilities, and difficult to test (see their section 7).

**Table 2.** Some numerical codes used by cited investigations. ‘Type’ refers to either a stellar evolution code, or an *N*-body dynamics code, or both.

name	type	ref	used by
AMUSE	both	[318]	[276]
BSE	stellar	[221]	[176,278]
MESA	stellar	[319,320]	[,^131^,^226^,^241^]
SSE	stellar	[27]	[24,32,144,264,277]
STAREVOL	stellar	[321]	[228]
STARS	stellar	[322]	[25,230]
Hermite	*N*-body	[323]	[277]
Mercury Bulirsch-Stoer	*N*-body	[324]	[24,59,61,106,144,208,264,325]
Mercury Hybrid	*N*-body	[324]	[61,271,273,275,326]
Mercury Radau	*N*-body	[324]	[259,260]
PKDGRAV	*N*-body	[327]	[59,295]

Previously, Lazio *et al.* [187] and Löhmer [188] placed limits on the masses of dust discs in other millisecond pulsars. However, unlike for PSR B1937+21, these other pulsars did not have timing residuals that were fit to specific asteroid belt or disc architectures [186]. Bryden *et al.* [189] searched for a dust disc in PSR B1257+12, but were not able to exclude the presence of a Solar system-like asteroid belt with a mass as large as 0.01*M*_⊕_ and SBs with radii up to 100 km. The X-ray pulsar or young magnetar 4U 0142+61 might host a disc [190,191], although the infrared excess in that system instead could be explained by magnetospheric emission [192]. Also, anomalous timing and radio emission signatures of the pulsar PSR J0738-4042 [193] cannot be explained by known stellar evolutionary processes. Such abrupt changes can be caused by encounters with asteroids, as argued by the authors.

Cordes & Shannon [194] expect asteroids to enter pulsar magnetospheres and create the largest detectable signals for large spin periods, large spin-down ages, large magnetospheres, low surface temperatures and low non-thermal luminosities (see their fig. 1). They also posed that interstellar comets impacting with circumpulsar discs may produce observable episodic behaviour. They stated that for an AU-scale dense disc with a high optical thickness, events may occur at the rate of once per year. Mitrofanov [195] postulated that these events may produce gamma ray bursts and prompt a ‘re-ignition’ of the pulsar.

## 4. Stellar mass ejecta

Having motivated the study of post-MS planetary systems, I now turn to important forces in these systems. Stellar mass loss is arguably the most important driver of all post-MS forces because it non-negligibly affects the orbits of all SBs at all distances (figure 2). In this vein, the classical mechanics-based mass-variable two-body problem has gained renewed importance with the mounting discoveries of post-MS planetary systems. For decades, this same problem has been relevant and applicable to binary stars, which represented the physical picture envisaged by early (pre-exoplanet era) studies.

The mass variable two-body problem is dynamically rich and not energy conserving. The problem can be broken down into the following questions: (i) How many bodies are losing mass? (ii) Is the mass loss isotropic? (iii) Is the mass loss instantaneous? (iv) Does a natal kick accompany the mass loss? (v) Are the bodies assumed to be point masses? (vi) How are one or both bodies dragged through the ejecta? These questions are not mutually exclusive, complicating a straightforward presentation. I proceed by grouping the first four questions into §4.1, the fifth question into §4.2 and the last question into §4.3.

### 4.1 The mass-variable point-mass two-body problem

Published investigations of the problem itself date back to the late nineteenth century: Razbitnaya [196] provided a brief historical review. Table 1 of that paper exhaustively lays out the equations of motion in 22 different cases, depending on the reference frame, relative velocities, whether one or both of the masses is variable, and whether the mass loss is isotropic. In all cases, both bodies are assumed to be point masses. For most post-MS planetary science applications, when the SB mass is considered fixed, rows #6 (anisotropic mass loss) and #7 (isotropic mass loss) of that table provide the relevant equations. When the SB itself is shedding mass (perhaps due to atmospheric evaporation; see §6.1.2 of this paper) or when its accreting the mass ejected from the star, then the equations in rows #1 (anisotropic mass loss) and #2 (isotropic mass loss) should instead be used.

#### 4.1.1 Isotropic mass loss set-up

In the most general point-mass isotropic mass loss case where both bodies are losing mass (row #2), the equation appears to be no different from the fixed masses case:

4.1 $$\frac{d^{2}\text{r}}{dt^{2}}=-\frac{G(M_{⋆}+M_{\mathrm{SB}})}{r^{3}}\text{r}.$$

What is different is that both masses are functions of time. In order to help understand the implications and provide physical intuition for their meaning, John D. Hadjidemetriou and Tuken B. Omarov independently, and using different approaches in different languages, derived in 1962–1963 the corresponding equations of motion for isotropic mass loss in planetary orbital elements [197,198]. The lack of internet and political considerations in the early 1960s prevented the eventual good friends from knowing about each other’s work until long after.

Both Hadjidemetriou and Omarov realized that equation (4.1) may be expressed as

4.2 $$\frac{d^{2}\text{r}}{dt^{2}}=-\frac{G(M_{⋆}(t=0)+M_{\mathrm{SB}}(t=0))}{r^{3}}\text{r}-\frac{1}{2(M_{⋆}(t)+M_{\mathrm{SB}}(t))}\frac{d(M_{⋆}(t)+M_{\mathrm{SB}}(t))}{dt}\text{r},$$

such that the classic orbitally static two-body problem is perturbed by a single mass loss term. Isolating this term helps quantify the motion. Equation (4.2) is derived from equation (4.1) by determining the total derivative of velocity with respect to time. This derivative is the sum of the partial derivative with (*M*_★_+*M*_SB_) held constant, plus the partial derivative with *r* held constant. The results of their derivations of the equations of motion entirely in terms of planetary elliptical orbital elements are as follows. I write them in terms of both true anomaly *f* and eccentric anomaly *E*:

4.3 $$\begin{array}{rlr} \frac{da}{dt} & =-\frac{a(1+e^{2}+2e\cos f)}{1-e^{2}}\frac{{\dot{M}}_{⋆}+{\dot{M}}_{\mathrm{SB}}}{M_{⋆}+M_{\mathrm{SB}}} & =-a\left(\frac{1+e\cos E}{1-e\cos E}\right)\frac{{\dot{M}}_{⋆}+{\dot{M}}_{\mathrm{SB}}}{M_{⋆}+M_{\mathrm{SB}}}, \end{array}$$

4.4 $$\begin{array}{rlr} \frac{de}{dt} & =-(e+\cos f)\frac{{\dot{M}}_{⋆}+{\dot{M}}_{\mathrm{SB}}}{M_{⋆}+M_{\mathrm{SB}}} & =-\left[\frac{(1-e^{2})\cos E}{1-e\cos E}\right]\frac{{\dot{M}}_{⋆}+{\dot{M}}_{\mathrm{SB}}}{M_{⋆}+M_{\mathrm{SB}}}, \end{array}$$

4.5 $$\begin{array}{rl} \frac{di}{dt} & =0\;=0, \end{array}$$

4.6 $$\begin{array}{rl} \frac{d\Omega }{dt} & =0\;=0 \end{array}$$

4.7 $$\begin{array}{rl} \;\text{and}\;\frac{d\omega }{dt} & =-\left(\frac{\sin f}{e}\right)\frac{{\dot{M}}_{⋆}+{\dot{M}}_{\mathrm{SB}}}{M_{⋆}+M_{\mathrm{SB}}}=-\left[\frac{\sqrt{1-e^{2}}\sin E}{e(1-e\cos E)}\right]\frac{{\dot{M}}_{⋆}+{\dot{M}}_{\mathrm{SB}}}{M_{⋆}+M_{\mathrm{SB}}}. \end{array}$$

Here *Ω* and *ω* refer to the longitude of ascending node and argument of pericentre. In order to complete the sets, the evolution of the anomalies themselves are

4.8 $$\frac{df}{dt}=-\frac{d\omega }{dt}+\frac{n{\left(1+e\cos f\right)}^{2}}{{\left(1-e^{2}\right)}^{3/2}}$$

and

4.9 $$\;\frac{dE}{dt}=-\frac{1}{\sqrt{1-e^{2}}}\frac{d\omega }{dt}+\frac{n}{1-e\cos E}.$$

In these equations, *n*=*G*^1/2^(*M*_★_+*M*_SB_)^1/2^*a*^−3/2^ represents the mean motion. Between the mid-1960s and the 2010s, these relations have not appeared often in other literature. One notable example is Alexander *et al.* [199], whose equations (3.11)–(3.13) can be reduced to the above relations. Equation (4.3) illustrates that the semi-major axis always increases due to isotropic mass loss. Together equations (4.3) and (4.4) may be combined to show that the orbital pericentre also always increases. The argument of pericentre is not defined for circular orbits and changes rapidly for near-circular orbits.

As observed by Adams *et al.* [200], a danger of using osculating Keplerian orbital elements is the interpretation of their non-Keplerian time evolution: for example, apparent oscillations in *e* can mask the motion’s actual smooth spiral. Iorio [201] further emphasized the importance of considering the time evolution of all the orbital elements concurrently to obtain a true picture of the motion. An alternative way of describing the motion is through a radial equation of motion [202]. Adams *et al.* [200] recently developed a dimensionless form of this equation and derived resulting relations for time-dependent mass-loss prescriptions in this framework.

#### 4.1.2 Anisotropic mass loss set-up

Equations (4.3)–(4.9) assume that the mass is lost isotropically. If one removes this assumption, and models direction-dependent mass loss and differential mass ejecta speeds, then additional terms appear in the equations. These terms are given by eqns 34–38 of [203]^2^ and are expressed in terms of mass flux relations from their eqn 4. These modified equations demonstrate that (i) the inclination and longitude of ascending node do change with time, and that (ii) anisotropy becomes more important the further away an SB is, as the anisotropic terms contain an additional factor of $\sqrt{a}$. Veras *et al.* [203] proved that mass-loss variations must be asymmetric about the stellar equator in order to affect the motion of SBs. They showed that for most post-MS applications with SBs within a few hundred AU, isotropy is an excellent approximation.

Although motivated by atmospheric evaporation on the MS, the mass loss formalisms presented by [205,206] provide potentially useful alternative approaches. Iorio [205] expressed the mass loss term in radial and tangential components, and then applied the Gaussian equations in that coordinate system; a variation on Omarov’s approach [197]. Boué *et al.* [206] instead treated radial mass loss from a solid angle patch of the atmosphere of one body (different from the one-dimensional jets from [203]) so that the mass emanates in a conical shape.

Parriott & Alcock [207] modelled anisotropy of mass loss in a different manner: through a kick, or a velocity impulse, of the star. Their equations of motion (in their eqn 1, which must contain a typographic error) include a term which requires *a priori* knowledge of the star’s final (WD) mass given a velocity impulse prescription.

#### 4.1.3 Isotropic non-instantaneous mass loss solutions

Veras *et al.* [208] extensively analysed equations (4.3)–(4.9), and observed that they can be derived via either perturbation techniques or the vis viva equation, combined with conservation of angular momentum expressed in polar coordinates. They also found importantly that the osculating pericentre monotonically increases with mass loss (their eqn 21), regardless of the mass loss prescription chosen. Although early studies like Hadjidemetriou [209] used the equations to show that orbital eccentricity changes are triggered by high mass loss, even for an initially circular orbit, the mounting observations of post-MS systems have led to a renewed interest in this problem.

*Parametrizing solutions.* Equations (4.3)–(4.9) do not appear to have a complete analytical solution. They do, however, admit two well-defined solution regimes and a difficult-to-characterize transition regime. In order to identify these solution regimes, consider the (dimensionless) scaled ratio of the timescales of orbital period to mass loss

4.10 $$\Psi \equiv \frac{{\dot{M}}_{⋆}}{M_{⋆}n}=\frac{1}{2\pi }\left(\frac{{\dot{M}}_{⋆}}{M_{⊙}\;{\mathrm{yr}}^{-1}}\right){\left(\frac{M_{⋆}}{M_{⊙}}\right)}^{-3/2}{\left(\frac{a}{\mathrm{AU}}\right)}^{3/2}.$$

When *Ψ*≪1, the system is commonly said to be in the ‘adiabatic’ regime. When *Ψ*≫1, the system is in a regime without a widely used moniker. The transition regime lies between these two extremes, and *Ψ* itself changes with time (eqn 16 of [208]) so that if mass loss continued indefinitely, the system would enter the *Ψ*≫1 regime from the adiabatic regime. The term ‘adiabatic’ in this context does not refer to heat, but rather conservation of eccentricity. The reason is that when *Ψ*≪1, the right-hand-sides of equations (4.4) and (4.7) may be approximated as zero. Further, in this limit, equation (4.3) simplifies to: d*a*/d*t*=−(*M*_★_+*M*_SB_)^−1^(d*M*_★_/d*t*).

As indicated by the maximum mass loss rates in figure 3 and equation (4.10), *Ψ*≪1 for the vast majority of known exoplanets (because *a*<100 AU and $M_{⋆}^{\left(\mathrm{MS}\right)}\leq 3\;M_{⊙}$ in almost every case). Consequently, these exoplanets will expand, but not stretch, their osculating orbits during post-MS mass loss by a factor approximately equal to $\left(M_{⋆}^{\left(\mathrm{MS}\right)}/M_{⋆}^{\left(\mathrm{WD}\right)}\right)$. During this process, *Ψ* remains much less than unity.

*Critical angles.* However, exo-Oort clouds, exo-scattered discs and some exoplanets (like WD 0806-661 b from [132]) have a more complex post-MS fate. For these systems, either *Ψ*∼1 or *Ψ*≫1. In the latter case, special solutions of the equations of motion exist, as outlined in section 2.6.2 of [208]. Related fundamental properties of the two-body problem derived in section 2.5 of [208] are the two critical true anomalies *f*_crit_

4.11 $$f_{\mathrm{crit}}\equiv \pi \pm \frac{1}{2}\cos\left(\frac{7}{9}\right)\approx 160.5^{\circ }\;\mathrm{and}\;199.5^{\circ }$$

at which the orbital eccentricity incurs an initial decrease at the onset of mass loss. Consequently, these values mark the boundaries at which some eccentric SBs for *Ψ*≫1 are protected from escape (fig. 16 of [208] and figs 9–10 of [210]).

*Transition to non-adiabaticity.* Based on angular momentum conservation, non-adiabatic motion does not ensure that the expansion of the semi-major axis proceeds faster than the adiabatic rate unless the orbit is initially circular (see eqns 1–3 of [176]). The transition regime can be wide in extent, spanning an order of magnitude in *a*^(*MS*)^ (see e.g. the bottom two panels in fig. 10 of [210]). In the top panel, the mass loss is strong enough to ensure *Ψ*≫1. For the most non-adiabatic of all the plots in that top panel (bottom-rightmost plot), the analytic values of *f*_crit_ well predict which systems eventually feature escape.

Debes & Sigurdsson [211] mentioned an important caveat to the adiabatic transition: the mass loss rate (from the orbit, and not the star) is dictated by the asymptotic wind velocity and not the ejection velocity. If, at Oort cloud distances, this speed is several orders of magnitude less than the escape speed, then the lower wind crossing time effectively decreases the mass loss rate. Consequently, *Ψ* might decrease enough for the evolution to still be adiabatic.

*Mass loss prescriptions.* Up until now, I have not assumed a particular analytic form for the mass loss prescription. Real stars do not follow simple prescriptions, particularly if they lose significant mass on both the RGB and AGB and pulse violently. Regardless, separately each branch has been fit to linear or exponential forms. Very early work from the 1890s [212,213] assumed dependencies with time. In 2009, Rahoma *et al.* [214] summarized (in their section 3) different mass-loss prescriptions used throughout the literature.

More recently, fig. 4 of [215] provided a comparison of the eccentricity excitation due to both linear and exponential mass loss, supposing the same total amount of mass lost. Adams *et al.* [200] adopted a dimensionless mass loss prescription index dependence, and used it to derive an explicit criterion for SB escape (see their eqn 86).

*A Hamiltonian framework.* Rahoma *et al.* [214] also treated the point-mass two-body non-instantaneous mass loss problem in the Hamiltonian framework. In their section 2, they provided a brief historical review of the problem with many references not listed here. One such reference is the classically entertaining paper of Deprit [216], which discusses, among other topics, time-dependent Delaunay transformations.

#### 4.1.4 Isotropic instantaneous mass loss solutions

Now consider the limit Ψ→∞, which is relevant (although technically not ever realized) for SNs. An advantage of modelling ‘instantaneous’ or ‘impulsive’ mass loss is that no assumptions about mass loss rates nor wind speeds are necessary: Hadjidemetriou [209] showed in this case how the stability of these systems crucially depends on the true anomaly at mass loss, with periastron and apastron being the limiting cases. Nevertheless, a SN process often creates a velocity ‘kick’ from asymmetries within the exploding star. The link between the mass lost and the magnitude and direction of the kick is unclear. Therefore, one best treat the general case of impulsive mass lost plus an arbitrary kick velocity equal to ***v***^(NS)^−***v***^(MS)^, which reduces to the mass-loss only situation in the limit of a zero-kick velocity.

Early treatments of this problem [217,218] predate confirmed exoplanets and were motivated by a potential pulsar planet at the time. Both studies, in 1970, considered the effect of instantaneous mass loss on a planet’s orbit due to a SN. Only 13 years later, however, was a comprehensive and general treatment presented [219].

*Explicit expressions.* Hills [219] used angular momentum and energy conservation, and the assumptions of instantaneous mass loss from a point-mass MS star, to provide explicit expressions for *a*^(NS)^, *e*^(NS)^ and the critical value of $M_{⋆}^{\left(\mathrm{NS}\right)}/M_{⋆}^{\left(\mathrm{MS}\right)}$ for which the SB escapes, in terms of only MS orbital parameters (including the true anomaly *f*). The expressions for the semi-major axis and mass ratio include an arbitrary kick, but the eccentricity expression does not because an additional degree of freedom would have to be introduced (see his section IIIb of [219]). By repackaging his eqn 13, including an arbitrary kick, I obtain

4.12 $$\begin{array}{rl} a^{\left(\mathrm{NS}\right)} & =\left[a^{\left(\mathrm{MS}\right)}\frac{M_{⋆}^{\left(\mathrm{NS}\right)}+M_{\mathrm{SB}}}{M_{⋆}^{\left(\mathrm{MS}\right)}+M_{\mathrm{SB}}}\right]\\ & \;\times {\left[1-\frac{2\left(1+e^{\left(\mathrm{MS}\right)}\cos f^{\left(\mathrm{MS}\right)}\right)}{1-{e^{\left(\mathrm{MS}\right)}}^{2}}\left(\frac{M_{⋆}^{\left(\mathrm{MS}\right)}-M_{⋆}^{\left(\mathrm{NS}\right)}}{M_{⋆}^{\left(\mathrm{MS}\right)}+M_{\mathrm{SB}}}\right)-\frac{a^{\left(\mathrm{MS}\right)}({v^{\left(\mathrm{NS}\right)}}^{2}-{v^{\left(\mathrm{MS}\right)}}^{2})}{G(M_{⋆}^{\left(\mathrm{MS}\right)}+M_{\mathrm{SB}})}\right]}^{-1}, \end{array}$$

which I have written in a form that illustrates how the adiabatic approximation (first square brackets) is modified. The SB will escape the system if, including an arbitrary kick (eqn 15 of [219])

4.13 $$M_{⋆}^{\left(\mathrm{MS}\right)}-M_{⋆}^{\left(\mathrm{NS}\right)}>\left[\frac{M_{⋆}^{\left(\mathrm{MS}\right)}+M_{\mathrm{SB}}}{2}\right]\left(\frac{1-{e^{\left(\mathrm{MS}\right)}}^{2}}{1+e^{\left(\mathrm{MS}\right)}\cos f^{\left(\mathrm{MS}\right)}}\right)\left(1-\frac{a^{\left(\mathrm{MS}\right)}({v^{\left(\mathrm{NS}\right)}}^{2}-{v^{\left(\mathrm{MS}\right)}}^{2})}{G(M_{⋆}^{\left(\mathrm{MS}\right)}+M_{\mathrm{SB}})}\right).$$

I write equation (4.13) in a form which illustrates how the oft-used notion that half of the original mass (in square brackets) must be lost for escape to occur is modified by other parameters. For high values of *e*^(MS)^, this modification may be severe (see fig. 12 of [208]). In equations (4.12 and 4.13), the expression *v*^(NS)^^2^−*v*^(MS)^^2^ may be replaced by an expression with the kick velocity, kick angle and *v*^(MS)^. The final eccentricity, without an arbitrary kick, is the following repackaged form of their eqn 6:

4.14 $$\begin{array}{rl} {e^{\left(\mathrm{NS}\right)}}^{2} & =1-(1-{e^{\left(\mathrm{MS}\right)}}^{2})\\ & \;\times {\left[\frac{M_{⋆}^{\left(\mathrm{NS}\right)}+M_{\mathrm{SB}}}{M_{⋆}^{\left(\mathrm{MS}\right)}+M_{\mathrm{SB}}}\right]}^{-2}\left[1-2\left(\frac{M_{⋆}^{\left(\mathrm{MS}\right)}-M_{⋆}^{\left(\mathrm{NS}\right)}}{M_{⋆}^{\left(\mathrm{MS}\right)}+M_{\mathrm{SB}}}\right)\left(\frac{1+e^{\left(\mathrm{MS}\right)}\cos f^{\left(\mathrm{MS}\right)}}{1-{e^{\left(\mathrm{MS}\right)}}^{2}}\right)\right]. \end{array}$$

*Modern re-derivations.* Later studies have attempted to reproduce special cases of equations (4.12)–(4.14), without kicks, and typically under the (usually secure) assumption that *M*_SB_≪*M*_★_. Veras *et al.* [208] provided the mass ratio boundedness condition in this case in their eqn 48, except the sign of their inequality should be flipped. Even with the above assumption, the expressions for the mass ratio and final eccentricity in eqns 1–2 of [220] do not appear to exactly agree with a reduction of the equations from [219]. Other, alternative formulations of the orbital evolution resulting from SN kicks have been developed (see, e.g. appendix A of [221]).

Equations (4.12)–(4.14) are useful because they are explicit closed equations for which functional dependencies can quickly be read off. However, if one wished to apply impulsive mass loss together with a kick in an arbitrary Cartesian direction, and derive the post-kick orbital elements, one could instead use the relations in appendix B of [67]. In the most general case, where the system is in an arbitrary frame, the equations in appendix A of [222], along with those in their section 2, provide a starting point for deriving explicit expressions of post-kick elements.

### 4.2 The mass-variable solid body two-body problem

So far, the vast majority of post-MS planetary investigations have treated orbital changes due to stellar mass loss assuming that the SB is a point mass. However, as models become more detailed and take into account physical changes to SBs, one may wish to lift the point mass assumption.

#### 4.2.1 Orbital evolution

If the SB is treated as a solid body, then it will increase in mass through accretion by the stellar wind. Consequently, equation (4.1) is no longer strictly usable. Because mass is no longer ejected from the orbit in the direction of the SB, the mass loss becomes anisotropic, leading to more complex equations (§4.1.2). Hadjidemetriou [198] derived compact expressions in his eqn 44 for how the orbital motion would change in this case, but assumed that the ejecta was moving at the speed of light, and did not account for gravitational focusing. In any case, incorporating solid body accretion into the orbital equations of motion would need to account for both extra terms in the equations of motion and the time-dependent change in the SB mass.

#### 4.2.2 Physical evolution

The level of accretion onto the SB itself might be significant. Small enough SBs like pebbles might accrete an amount of mass so great that the pebble might be transformed into a boulder, or just be destroyed. Large SBs like planets with atmospheres could have the composition and nature of those atmospheres permanently altered.

What is the accretion rate onto the SB? The literature is replete with answers to this question. Many answers differ depending on whether the authors assumed, for example, that gravitational focusing is important, the SB is on a circular orbit, and the wind speed is much greater than *v*. Some expressions include an explicit dependence on ${\dot{M}}_{⋆}$, whereas others instead show a dependence on *ρ*_wind_. These two important quantities are often related through a constant-velocity spherically symmetric (isotropic) mass loss assumption, and when the ‘ejected mass is stratified in concentric spherical layers of constant density’ [209]. Consequently (his eqn 2)

4.15 $$\rho_{\mathrm{wind}}=\frac{{\dot{M}}_{⋆}}{4\pi r^{2}|v_{\mathrm{wind}}|}.$$

By using equation (4.15), one may compare different expressions for ${\dot{M}}_{\mathrm{SB}}$ which appear in the literature. These include eqn 2 of [223], eqns 1–2 of [224], eqn 1 of [225], eqn 11 of [226] and eqn 4 of [131]. The formulation from [221] is expressed in terms of *e*: repackaging their eqns 6–9 gives

4.16 $$\left\langle \frac{dM_{\mathrm{SB}}}{dt}\right\rangle =-\frac{dM_{⋆}}{dt}\frac{3}{4a^{2}\sqrt{1-e^{2}}}{\left(\frac{GM_{\mathrm{SB}}}{v_{\mathrm{wind}}^{2}}\right)}^{2}{\left[1+\frac{G^{2}{\left(M_{⋆}+M_{\mathrm{SB}}\right)}^{2}}{a^{2}v_{\mathrm{wind}}^{2}}\right]}^{-3/2}.$$

By using expressions for the accretion rate onto the SB some authors [131,223,224], then estimated the total mass accreted by the SB. However, doing so necessitates integrating with respect to time, and ${\dot{M}}_{⋆}$ is a non-monotonic function of time, initial mass and metallicity along the GB. Nevertheless, Spiegel & Madhusudhan [131] found that brown dwarfs and large planets could accrete an amount of mass that is a multiple of their MS atmospheric masses.

### 4.3 Stellar wind/gas/atmospheric drag

Up until now, I have neglected the physical interaction between the ejected stellar mass and the SB. This assumption might be good for large SBs like planets, and/or tenuous stellar winds. However, for typical GB stars, the interaction between the stellar wind and smaller SBs like pebbles or asteroids may be significant. I also include here the treatment of SBs within stellar atmospheres. Although strictly not under the umbrella of ‘stellar ejecta’, stellar atmospheres provide a similar medium within which drag takes place, just with a (typically) higher density than the stellar wind. Both the wind and atmosphere are considered to be gaseous media.

#### 4.3.1 Physical evolution

Sufficiently small SBs are in danger of catastrophically disrupting due to ram pressure. For spherical SBs which are hydrostatic, isothermal and self-gravitating, eqn 1 of [226] provides the following condition for stability against disruption:

4.17 $$g_{\mathrm{SB}}≲\frac{2\pi \rho_{\mathrm{SB}}\rho_{\mathrm{wind}}v^{2}}{R_{\mathrm{SB}}(\rho_{\mathrm{SB}}^{2}-\rho_{\mathrm{wind}}^{2})},$$

where *g* is surface gravity. They then specialize to the case *ρ*_SB_≫*ρ*_wind_ and $g\approx GM_{\mathrm{SB}}/R_{\mathrm{SB}}^{2}$. These simplifications yield the following range of masses for which disruption would occur (their eqn 2). I rewrite this equation first from the perspective on atmospheric infall and then for GB winds outside of the atmosphere:

4.18 $$\begin{array}{rl} M_{\mathrm{SB}} & ≲0.02M_{⊕}{\left(\frac{\rho_{\mathrm{wind}}}{10^{-4}\;g\;{\mathrm{cm}}^{-3}}\right)}^{3/2}{\left(\frac{\rho_{\mathrm{SB}}}{2\;g\;{\mathrm{cm}}^{-3}}\right)}^{-2}{\left(\frac{v}{100\;\mathrm{km}\;s^{-1}}\right)}^{3} \end{array}$$

4.19 $$\begin{array}{rl} & ∼100\;\mathrm{kg}{\left(\frac{\rho_{\mathrm{wind}}}{10^{-16}\;g\;{\mathrm{cm}}^{-3}}\right)}^{3/2}{\left(\frac{\rho_{\mathrm{SB}}}{2\;g\;{\mathrm{cm}}^{-3}}\right)}^{-2}{\left(\frac{v}{10\;\mathrm{km}\;s^{-1}}\right)}^{3}. \end{array}$$

Although approximate, equation (4.18) suggests that giant planets and brown dwarfs should survive catastrophic disruption while inside stellar atmospheres, and equation (4.19) suggests that asteroids, comets, planets and brown dwarfs will easily survive stellar winds. The situation for boulders and pebbles requires further analysis: Jura [224] assumes that an SB ‘is destroyed if it encounters its own mass in the wind’ due to hydrodynamic friction, whereas Dong *et al.* [227] claimed that when the drag force exceeds the parent star gravitational force, then the SB is ‘entrained’ by the wind.

#### 4.3.2 Orbital evolution

*Context for drag.* Surviving SBs will have their orbits altered by interactions with the surrounding medium. The existing post-MS planetary literature has used a variety of names and expressions for the resulting forces. I group the forces into two categories: one consisting of a purely gravitational force due to the wake generated, and one due to friction. I refer to the former as ‘gravitational drag’ and the latter as ‘frictional drag’, in line with Villaver and colleagues [225,228]. Willes & Wu [46] and Rybicki & Denis [229] referred to the frictional drag as ‘bow shock drag’. Schröder & Smith [230] referred to the combined effects of gravitational drag and frictional drag as ‘dynamical friction’. In combined expressions, drag terms containing *G* typically refer to gravitational drag and those without *G* refer to frictional drag. Schröder & Smith [230] suggested that within the atmosphere, if *v*≈(2−3)*v*_sound_, where *v*_sound_ is the sound speed, then both gravitational drag and frictional drag play nearly equal roles.

*Gravitational drag.* However, gravitational drag is negligible outside of a stellar atmosphere. Consequently, the seminal pre-exoplanet era gravitational drag study of Alexander *et al.* [199] defined a type of cutoff in their eqn 2.1. Goldstein [231] claimed that this value is ‘usually defined as the neutral point between planet and star’, whereas Livio [232] took this value as ‘the mean radius of the planet’s Roche lobe’ and went on to derive analytical relations with this assumption. Alexander *et al.* [199] then used their gravitational drag expression and parametrized *v*_wind_ into radial and tangential components in order to derive evolution equations in orbital elements (their eqns 3.11–3.13). These equations also contain isotropic mass loss terms and are sufficiently general to reduce to my equations (4.3)–(4.7) in the appropriate limits.

In a seminal later study, Ostriker [233] used time-dependent linear perturbation theory to derive an expression for the gravitational drag force, which she denotes the ‘dynamical drag force’. This expression has subsequently been used for post-MS planetary system applications by others [225,228,230]. For Mach numbers of interest within the atmospheres of GB stars both eqn 5 of [225] and eqn 4 of [228] give

4.20 $$F_{\mathrm{drag}}^{\left(\mathrm{grav}\right)}=\frac{2\pi G^{2}M_{\mathrm{SB}}^{2}\rho_{\mathrm{wind}}}{v_{\mathrm{sound}}^{2}},$$

where *F* denotes force and *ρ*_wind_ is the density of the atmosphere. Combining equation (4.20) with equation (4.15) would not be appropriate in this context because the star need not be losing mass. Schröder & Smith [230] state that a typical value of *v*_sound_ within the stellar chromosphere is about 8 km s^−1^. In their study on SB engulfment into GB stars, Villaver & Livio [225] found that *v*/*v*_sound_>3 always. They adopted *v*_wind_=5 km s^−1^, whereas Mustill *et al.* [176] adopted a wind speed of 10–15 km s^−1^. In general, ***v***_wind_∝*M*_★_ (footnote #1 in [230]).

*Frictional drag.* The frictional drag force has appeared in several different forms in the post-MS literature: (i) eqn 6 of [225] and eqn (4) of [228], (ii) eqn 3 of [227] and (iii) eqns 37–39 of [234]. The last of these references considers both the Epstein and Stokes regimes of motion, given respectively by the upper and lower relations in:

4.21 $$F_{\mathrm{drag}}^{\left(\mathrm{fric}\right)}=\left\{ \begin{array}{ll} \left(\frac{M_{\mathrm{SB}}\rho_{\mathrm{wind}}v_{\mathrm{sound}}}{\rho_{\mathrm{SB}}R_{\mathrm{SB}}}\right)({\text{v}}_{\mathrm{wind}}-\text{v}), & R_{\mathrm{SB}}\ll \zeta \\ \left(\frac{M_{\mathrm{SB}}\rho_{\mathrm{wind}}U}{\rho_{\mathrm{SB}}R_{\mathrm{SB}}}\right)({\text{v}}_{\mathrm{wind}}-\text{v})|{\text{v}}_{\mathrm{wind}}-\text{v}|, & R_{\mathrm{SB}}\gg \zeta , \end{array}\right.$$

where *ζ* is the mean free path length of the gas. Eqn 56 of [234] provides the following numerical constraint on the path length: *ρ*_wind_*ζ*∼10^−8^ kg m^−2^. The quantity *U* is given by

4.22 U={9[6RSBζvsound|vwind−v|]−1,Re≤19[6RSBζvsound|vwind−v|]−0.6,1≤Re≤8000.165,Re≥800,}

where Re represents the Reynolds number of the flow and is given by

4.23 $$\text{Re}=\frac{6R_{\mathrm{SB}}}{\zeta v_{\mathrm{sound}}}|{\text{v}}_{\mathrm{wind}}-\text{v}|.$$

## 5. Star–planet tides

The vast majority of known exoplanets will experience violent tidal interactions with their parent stars. Almost every planet discovered by transit photometry is close enough to its parent star for tides to eventually play a significant role in its evolution. Consequently, the last 15 years have seen a surge in tidal-based investigations around MS stars. However, the results of this effort, unlike for mass loss in the last section, have proven to be controversial. The physics involved in tidal dissipation is sufficiently complex that basic open questions remain unsolved: e.g. how close does a planet have to be to the stellar surface to be engulfed?

Because we have room to improve our understanding of tides on the MS, extrapolating to post-MS systems might seem difficult. Nevertheless, much recent progress has been made on GB star–planet tides.

### 5.1 Tidal theory

Important questions need to be answered before modelling a tidally-influenced system. (i) Do SBs induce tidal bulges in the star, does the star induce tidal bulges in the SB, or both? (ii) In each case, what is the dominant tidal mechanism acting? The answers depend on the properties of both the star and the SB; for a recent review of tides between stars and gas giant planets with zero solid matter, see [235]. Depending on the level of accuracy one seeks, Efroimsky & Makarov [236] proved that some classical tidal theories should be used with caution, or not at all. However, these theories provide appealing analytically tractable forms that have been implemented by much of the community.

*Only stellar tides matter.* For GB systems, large SBs like planets or brown dwarfs within a few AU will almost certainly induce a tidal bulge in GB stars because these stars will grow their convective envelopes to AU-scales (in fact, Mustill & Villaver [30] approximated the stellar radius to be equal to the radial extent of the envelope). Alternatively, GB stars are unlikely to induce tidal bulges in large SBs. Mustill & Villaver [30] and Villaver *et al.* [228] consider this possibility with a constant tidal lag model. By adopting a generous range (many orders of magnitude) of tidal quality factors, they find that planetary tides are negligible.

Stellar tides matter only if the SB is large enough: at least planet-sized. An asteroid or comet would likely induce too small a tidal bulge in the star to cause a significant orbital change (for quantification, see e.g. eqn 8 of [237], eqns 10–11 of [238] or eqns (5.1)–(5.2) below).

*Orbital retardation.* The dominant tidal mechanism for GB stellar tides is turbulent viscous dissipation in the envelope. Schröder & Smith [230] alternatively describe the process as ‘retardation of the equilibrium tide by interaction with convective motions’. This phenomenon has been described with standard mixing length theory in the pre-exoplanet era by Zahn [239,240], and has been recently employed by others [30,225,228,241,242]. Dissipation in the convective envelope causes the SB’s orbital semi-major axis and eccentricity to change. To leading order in eccentricity (an unfortunately restrictive but computationally feasible assumption) the orbital evolution is dictated through [30,228]

5.1 $$\begin{array}{r} \frac{da}{dt}=-\frac{a}{9t_{\mathrm{conv}}}\frac{M_{⋆}^{\left(\mathrm{env}\right)}}{M_{⋆}}\frac{M_{\mathrm{SB}}}{M_{⋆}}\left(1+\frac{M_{\mathrm{SB}}}{M_{⋆}}\right){\left(\frac{R_{⋆}}{a}\right)}^{8}\left[2p_{2}+e^{2}\left(\frac{7}{8}p_{1}-10p_{2}+\frac{441}{8}p_{3}\right)\right] \end{array}$$

and

5.2 $$\begin{array}{r} \frac{de}{dt}=-\frac{e}{36t_{\mathrm{conv}}}\frac{M_{⋆}^{\left(\mathrm{env}\right)}}{M_{⋆}}\frac{M_{\mathrm{SB}}}{M_{⋆}}\left(1+\frac{M_{\mathrm{SB}}}{M_{⋆}}\right){\left(\frac{R_{⋆}}{a}\right)}^{8}\left[\frac{5}{4}p_{1}-2p_{2}+\frac{147}{4}p_{3}\right], \end{array}$$

where $M_{⋆}^{\left(\mathrm{env}\right)}$ is the stellar envelope mass, *t*_conv_ is the eddy turnover timescale within the stellar envelope

5.3 $$t_{\mathrm{conv}}={\left[\frac{M_{⋆}^{\left(\mathrm{env}\right)}{\left(R_{⋆}-R_{⋆}^{\left(\mathrm{env}\right)}\right)}^{2}}{3L_{⋆}}\right]}^{1/3}$$

and *p*_1_, *p*_2_ and *p*_3_ are frequency components of the tidal force (below). In their study of planet–GB tides, Kunitomo *et al.* [241] provide the same expression for the eddy turnover timescale (their eqn 4) although the expression in eqn 31 of [221] is slightly different due to the convection zone reference point they use. Regarding equation (5.1) above, that equation contains individual frequency components, unlike eqn 1 of [241], eqn 8 of [243] and eqn 29 of [221].

The *p* components are functions of *a*, *M*_★_, *M*_SB_ and one’s choice about the nature of the dissipation through a functional dependence. This last choice, importantly, does not critically impact the tidal model for GB stars [228]. Consequently, by adopting the values from Laskar & Gastineau [225], the frequency components are well approximated (i.e. calibrated with observations of post-MS binaries by Verbunt & Phinney [244]) by

5.4 $$p_{l}\approx \frac{9}{2}\min\left[1,\left(\frac{4\pi^{2}a^{3}}{l^{2}G(M_{⋆}+M_{\mathrm{SB}}){\left[t_{\mathrm{conv}}\right]}^{2}}\right)\right]$$

where *l*={1,2,3}. See Section 4.4 of [30] for an analysis of the goodness of the approximation in equation (5.4).

By using an appropriate stellar evolution model (see §14) that computes the time evolution of the stellar envelope mass and radii, one can then integrate equations (5.1)–(5.4) to determine the SB’s orbital evolution. Note that these equations are secular, and hence cannot resolve changes on orbital timescales.

*Contribution of stellar spin.* Hidden in equations (5.1)–(5.4) is the omission of the GB star’s spin. A GB star’s spin is likely to be negligible due to conservation of spin angular momentum from the MS. Consequently, neglecting stellar spin in a model is likely a good approximation. The larger the SB, however, the more important a role any extant stellar spin might play. Nordhaus & Spiegel [242] used a different expression for the semi-major axis evolution, including stellar spin, that is similar to equation (5.1) but assumed circular orbits and a different parametrization for *p*. They also included a separate equation of motion for the stellar spin. In principle, one may derive equations for $\dot{a}$, $\dot{e}$ and ${\dot{s}}_{⋆}$, where *s*_★_ is the stellar spin, to arbitrary orders in eccentricity and spin, akin to the second-order treatment provided by Zahn [245] in his eqns 17–19.

*Tidal quality factor.* An alternative tidal approach is one that places the unknown physics of dissipation into a single parameter (in a similar way to the *α* viscosity parametrization in viscous discs): the tidal quality factor [246]. Equation (5.1) may be expressed in terms of the tidal quality factor (e.g. cf. eqns 1 and 8 of [241]). A tidal quality factor approach also allows one to generate evolution equations in terms of arbitrary eccentricities with no series expansion [247]. However, for GB stars, the formalism from Zahn [239] is better constrained than one with a tidal quality factor partly because (i) the time and frequency dependence of the tidal quality factor is unknown and (ii) the ‘constant time lag model’, which uses the quality factor, is inherently inconsistent when applied to SBs containing solid material [236].

*Analytical engulfment distances.* By employing a series of assumptions, a few investigations have derived explicit semi-analytical and analytical formulae for the minimum distance at which an SB would be engulfed during the GB phases. These derivations require assumptions about both tidal effects and prescriptions for the mass loss rates, meaning that during application one must keep in mind the accuracy sought. Informed by numerical simulations, eqn 7 of [241] provides scalings which indicate that the most important consideration for RGB engulfment is the maximum RGB stellar radius. The more general study of Adams & Bloch [243] assumed power-law dependencies for tidal dissipation (their eqn 6), and a power-law prescription for mass loss (their eqn 4), which includes a modification for AGB pulse mass loss (their eqn 27). Their final expression for the critical engulfment distance (their eqn 38) is a function of these power-law indices and other coefficients.

*Compact object tides.* For DA WDs and other compact objects where the convection zone is negligible, one must seek an alternative tidal approach than that from Zahn [239]. In compact objects, both radiative and convective regions produce tidal dissipation, and a coupled treatment (as in eqn 31 of [248]) might be desirable. Also, generally, if a WD and MS star have the same convective properties, then they will appear equivalent from the point of view of tidal dynamics.

### 5.2 Simulation results

The emphasis in GB–planet tidal investigations is on engulfment: how could planets survive and where and when do they die. The implications of the answers help determine what architectures can and should exist around WDs and help constrain GB planet discovery space. The differences in these answers among different studies [225,228,241] emphasize the sensitive dependence on the stellar models adopted.

**Figure 8.**
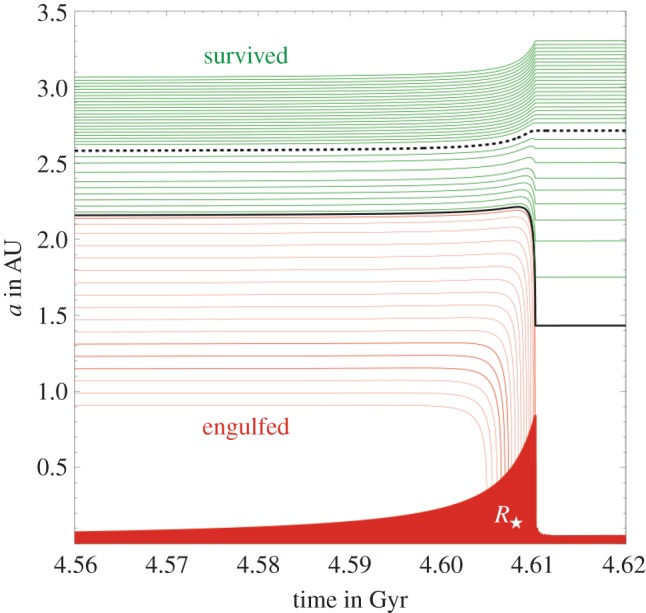
Cosmetically enhanced version of fig. 1 of [228]. The prospects for survival of Jupiter-mass planets orbiting a RGB star with $M_{⋆}^{\left(\mathrm{MS}\right)}=1.5\;M_{⊙}$ and evolving due to tides and mass loss. The stellar surface is given by the upper curve on the solid red shape. The red planetary tracks end in engulfment, whereas planets on green tracks remain safe. The solid black curve shows the closest planet that survives, and the dotted black curve illustrates the closest planet that is not visually affected by RGB tides on the scale of this plot.

The process of engulfment along the RGB is illustrated in figure 8, which displays the evolution (mass loss and tides) of a $M_{⋆}^{\left(\mathrm{MS}\right)}=1.5\;M_{⊙}$ star with Jupiter-mass planets that will be engulfed (red curves), that are affected by tides but not engulfed (green curves), and survive without being affected by tides (black curves). Note that surviving planets that are ‘stalled’ by GB tidal effects harbour WD–planet distances which are smaller than that predicted from adiabatic mass loss expansion alone.

**Figure 9.**
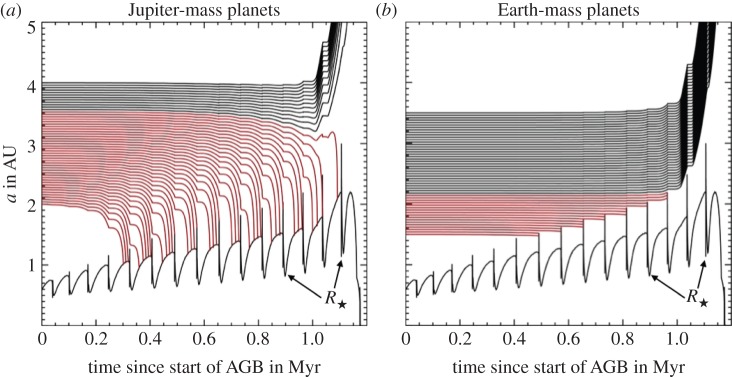
Cosmetically enhanced version of fig. 3 of [30]. The prospects for survival of Jupiter-mass planets (*a*) and Earth-mass planets (*b*) orbiting a AGB star with $M_{⋆}^{\left(\mathrm{MS}\right)}=2.0\;M_{⊙}$ and evolving due to tides and mass loss. Unlike in figure 8, here the stellar surface pulses. (*a*) Illustrates that surviving Jovian-mass planets must begin their orbits at least 20 per cent further away than the maximum stellar radius. (*b*) Earth-mass planets with starting orbits that are within the maximum stellar radius can survive.

If SBs survive the RGB phase, however, they might still be ingested during the AGB phase. Figure 9 illustrates the fate of Jovian-mass planets and Earth-mass planets around a thermally pulsing $M_{⋆}^{\left(\mathrm{MS}\right)}=2.0\;M_{⊙}$ AGB star. The plots (originally from [30]) demonstrate how lower-mass planets feel weaker tidal forces than giant planets. Consequently, the former can reside on MS orbits within the maximum AGB radius and still survive GB evolution. Not shown on these plots is how eccentricity affects engulfment: eccentric giant planet orbits can circularize more quickly than terrestrial planet orbits due to tides. This quick circularization acts as a protection mechanism for giant planets.

The results of Villaver *et al.* [228] have an important observational link: they show that the RGB star region which is void of planets (within about 0.5 AU) is too wide to have been depleted by tidal effects alone. Therefore, if the retired A star hosts actually have smaller masses, as argued by Lloyd [165], then the origin of the void is even a larger mystery.

## 6. Stellar radiation

The importance of stellar radiation in post-MS science is highlighted by a pre-exoplanet-era prediction [249] that the Solar system’s Kuiper belt will be eventually evaporated from GB radiation. Further, WD and pulsar radiation determine how volatile-rich planetary remnants behave and may dictate the shape and properties of surrounding debris discs.

### 6.1 Giant branch radiation

During stellar evolution, a GB star’s luminosity can reach a value that is several orders of magnitude greater than the current Sun’s (figure 3). The consequences are varied. SBs within hundreds of AU will feel the result, as the snowlines in those systems might extend out to that distance [67,249]. Pebbles, asteroids and comets (§6.1.1) incur orbital changes more complex than those from mass loss alone, and asteroids and comets might self-destruct through overspinning. Within debris discs, the size distribution and collision rates might change [32]. Giant planet and terrestrial planet atmospheres (§6.1.2) will be transformed both physically and chemically.

#### 6.1.1 Effect on pebbles, asteroids and comets

The total radiative effect on a two-body system consisting of a star and a solid body SB with no atmosphere yields the following expressions for the orbital and physical equations of motion.

*Orbital evolution.* I obtain the orbital equation of motion by combining eqns 9 and 29 of [234], yielding

6.1 $$\begin{array}{rl} \frac{d^{2}\text{r}}{dt^{2}} & =-\frac{G(M_{⋆}+M_{\mathrm{SB}})}{r^{3}}\text{r}+\frac{A_{\mathrm{SB}}L_{⋆}}{4\pi M_{\mathrm{SB}}cr^{2}}\left\{\left(1-\frac{\text{v}\cdot \text{r}}{cr}\right)\frac{\text{r}}{r}-\frac{\text{v}}{c}\right\}\\ & \;\times \left[Q_{\mathrm{abs}}I+Q_{\mathrm{ref}}I+\left\{H\left(R_{\mathrm{SB}}-\sqrt{\frac{K_{\mathrm{SB}}P_{\mathrm{SB}}^{\left(\mathrm{spin}\right)}}{\pi \rho_{\mathrm{SB}}C_{\mathrm{SB}}}}\right)\right\}w(Q_{\mathrm{abs}}-Q_{\mathrm{ref}})Y\right]. \end{array}$$

In equation (6.1), *c* is the speed of light, *A*_SB_ is the SB’s cross-sectional area, *Q*_abs_ is the SB’s absorption efficiency, *Q*_ref_ is the SB’s reflecting efficiency (or albedo), I is the 3×3 identity matrix, H is the Heaviside function, *K*_SB_ is the SB’s thermal conductivity, *P*^(spin)^_SB_ is the SB’s spin period, $C_{\mathrm{SB}}$ is the specific thermal capacity of the SB, *w* is a value between 0 and $\frac{1}{4}$ that indicates the extent of the temperature difference between the heated and non-heated sections of the SB (no difference corresponds to *w*=0) and Y is the 3×3 Yarkovsky matrix (given through eqns 13–20 and 28 of [234]).

The three terms in the square brackets in equation (6.1) correspond respectively to the acceleration caused by absorbed radiation, immediately reflected radiation and re-emission from delayed radiation. The motion resulting from this last term is known as the ‘Yarkovsky effect’, which has been acknowledged as a potential driver of planetary debris [95,61] and described further for particles in compact object debris discs (appendix A of [194] and section 2.2 of [250]), but not considered in more geometric detail until Veras *et al.* [234] (their section 2.6). The hallmark of the Yarkovsky effect, which is re-emission from delayed radiation, arises from a thermal imbalance and redistribution within the SB. Hence, if the SB is too small or spins too quickly, the Yarkovsky effect is not triggered. When the Yarkovsky effect vanishes, the tangential and radial components of the sum of the other two terms are known respectively as ‘Poynting-Robertson drag’ and, confusingly, ‘radiation pressure’. The term in the first set of curly brackets is the relativistically corrected direction of incoming radiation.

Other important features of this equation include the following. (i) The presence of the Heaviside function indicates that the Yarkovsky effect does not ‘turn on’ unless *R*_SB_ is above a critical size. Veras *et al.* [234] found that across the entire range of realistic values of *K*_SB_, *P*^(spin)^_SB_ and $C_{\mathrm{SB}}$, this critical value lies between 1 cm and 10 m (their fig. 2). (ii) Even below this threshold, equation (6.1) is applicable only when the SB is at least a few orders of magnitude greater than the wavelength of the radiation. Veras *et al.* [70] argued that for WDs, this wavelength is always less than a micrometre. (iii) Because, for spherical SBs, the acceleration due to radiation is inversely proportional to *R*_SB_, the orbits of planet-sized (thousands of km) SBs will only negligibly be affected by radiation.

In order to provide a rough idea of the potential importance of the Yarkovsky effect, I compare the averaged eccentricity changes expected from the Yarkovsky effect (eqn 108 of [234])

6.2 $$\begin{array}{rl} {\left\langle \frac{de}{dt}\right\rangle }^{\left(\mathrm{Yarkovsky}\right)} & =O\left(\frac{1}{c}\frac{A_{\mathrm{SB}}L_{⋆}}{8\pi M_{\mathrm{SB}}na^{3}}\right)∼\frac{0.08}{\mathrm{Myr}}{\left(\frac{M_{⋆}}{1M_{⊙}}\right)}^{-1/2}{\left(\frac{\rho_{\mathrm{SB}}}{2\;g\;{\mathrm{cm}}^{-3}}\right)}^{-1}\\ & \;\times {\left(\frac{R_{\mathrm{SB}}}{1\;\mathrm{km}}\right)}^{-1}{\left(\frac{a}{5\;\mathrm{AU}}\right)}^{-3/2}\left(\frac{L_{⋆}}{10^{3}L_{⊙}}\right) \end{array}$$

with those from the combined averaged effect of Poynting–Robertson drag and radiation pressure (eqn 109 of [234])

6.3 $$\begin{array}{rl} {\left\langle \frac{de}{dt}\right\rangle }^{\left(\mathrm{PR}+\mathrm{rp}\right)} & =O\left(\frac{1}{c^{2}}\frac{5A_{\mathrm{SB}}L_{⋆}}{8\pi M_{\mathrm{SB}}a^{2}}\right)∼\frac{1.8\times 10^{-5}}{\mathrm{Myr}}{\left(\frac{\rho_{\mathrm{SB}}}{2\;g\;{\mathrm{cm}}^{-3}}\right)}^{-1}\\ & \;\times {\left(\frac{R_{\mathrm{SB}}}{1\;\mathrm{km}}\right)}^{-1}{\left(\frac{a}{5\;\mathrm{AU}}\right)}^{-2}\left(\frac{L_{⋆}}{10^{3}L_{⊙}}\right). \end{array}$$

The four order-of-magnitude difference in the coefficients of the expressions is largely attributable to the difference in powers of (1/*c*) in equations (6.2 and 6.3). Averaging eliminates the (1/*c*) terms in the expressions for Poynting–Robertson drag and radiation pressure, but not for the Yarkovsky effect.

*Physical evolution.* For the physical evolution of the SB, I display an averaged spin equation of motion, where *s* is spin (eqn 1 of [251] or eqn 3 of [234]):

6.4 $$\left\langle \frac{ds_{\mathrm{SB}}}{dt}\right\rangle =\frac{j}{2\pi \rho_{\mathrm{SB}}R_{\mathrm{SB}}^{2}}\left(\frac{1}{a^{2}\sqrt{1-e^{2}}}\right)\left(10^{17}\;\mathrm{kg}\;m\;s^{-2}\frac{L_{⋆}}{L_{⊙}}\right),$$

an equation for radius reduction due to ablative mass loss from sublimation (eqn 15 of [252], eqn 9 of [224] and eqn 31 of [32]):

6.5 $$\frac{dR_{\mathrm{SB}}}{dt}=-\frac{1.5\times 10^{10}\;\text{kg}\;{\text{m}}^{-2}\;{\text{s}}^{-1}}{\rho_{\mathrm{SB}}}\sqrt{\frac{T_{\mathrm{SB}}^{\left(\mathrm{sub}\right)}}{T_{\mathrm{SB}}}}\text{exp}\left(-\frac{T_{\mathrm{SB}}^{\left(\mathrm{sub}\right)}}{T_{\mathrm{SB}}}\right)$$

and an equation for the average SB surface temperature, excluding any thermal imbalance between the hemispheres of the SB (eqn 3a of [249]):

6.6 $$\langle T_{\mathrm{SB}}\rangle ={\left[\frac{1}{\epsilon_{\mathrm{SB}}\sigma }\left(\frac{(1-Q_{\mathrm{ref}})L_{⋆}}{16\pi r^{2}}+S_{\mathrm{SB}}\frac{dM_{\mathrm{SB}}}{dt}\right)\right]}^{1/4}.$$

In equations (6.4)–(6.6), *j* is a value between 0 and 1 which indicates the extent of the asymmetry in the shape of the SB (no asymmetry corresponds to *j*=0), $T_{\mathrm{SB}}^{\left(\mathrm{sub}\right)}$ is the composition-dependent sublimation temperature of the SB, *ϵ*_SB_ is the SB’s emissivity, *σ* is the Stefan–Boltzmann constant, and $S_{\mathrm{SB}}$ is the SB’s specific heat of sublimation. See table 1 of [113] for a useful reference for sublimation temperatures *T*^(sub)^_SB_ of different materials, and fig. S6 of [125] for characteristic vapour pressures and mass loss rates for some refractory materials. Changes in spin due to radiation (equation (6.4)) is known as the ‘YORP effect’.

Five important points include: (i) equations (6.4)–(6.5) do not include physics arising from avalanches, micrometeoroid impacts or differentiated internal layers, all potentially important sources of modifications; (ii) equation (6.6) does not include the Yarkovsky effect, which would be affected by mass loss and any shape changes; (iii) the spin of the SB (equation (6.4)) is strongly tied to the value of *w* (in equation (6.1)) such that a stationary object corresponds to $w=\frac{1}{4}$; (iv) Farihi *et al.* [90] pointed out that if a volatile species such as water is contained in hydrated minerals like phyllosilicates, then the water will not sublimate until much higher temperatures than *T*^(sub)^_SB_ are reached; and (v) the expressions for the change in both *R*_SB_ and *M*_SB_ in equations (6.5)–(6.6) may be related through an assumption of a spherical SB shape.

How does the temperature of an SB change as a result of post-MS evolution? Equation (6.6) is just an approximation. Jura & Xu [92] performed a more sophisticated extension of earlier work [224], solving the one-dimensional heat conduction equation for asteroids which are assumed to be in hydrostatic equilibrium with a uniform density and no surface pressure. Jura & Xu [92] self-consistently treated orbital evolution and internal water evaporation for these uniform density asteroids for two stellar tracks: a $M_{⋆}^{\left(\mathrm{MS}\right)}=1\;M_{⊙}$ and a $M_{⋆}^{\left(\mathrm{MS}\right)}=3\;M_{⊙}$ star, each with a metallicity of *Z*_★_=0.0019. The thermal evolution is complex: sometimes at the tip of the AGB, the highest temperatures were found to occur beneath the surface.

An SB may spin so fast that it will tear itself apart. This possibility is not remote. In fact, nearly every asteroid in the Solar system’s asteroid belt with *R*_SB_=100 m–10 km will break apart in this manner after the Sun turns off of the MS [251]. The result is a potential debris field which could extend to thousands of AU. The critical spin period of 2.33 h at which breakup occurs is well-grounded in Solar system observations (see fig. 1 of [253]). More generally, by assuming the SB is a strengthless rubble pile, I can write the critical spin *s*_crit_ in terms of *ρ*_SB_ as (eqn 2 of [251])

6.7 $$s_{\mathrm{crit}}=2\pi \sqrt{\frac{G\rho_{\mathrm{SB}}}{3\pi }}=7.48\times 10^{-4}\frac{\mathrm{rad}}{s}{\left(\frac{\rho_{\mathrm{SB}}}{2\;g\;{\mathrm{cm}}^{-3}}\right)}^{1/2}.$$

#### 6.1.2 Effect on planetary atmospheres

The GB radiation may cause SB atmospheres to evaporate, either partially or fully. If the SB is all atmosphere, then one can estimate the maximum SB mass that would completely evaporate, particularly when inside of the stellar envelope [25,42,58]. Further, fig. 1 of [231] explicitly illustrates that different atomic and molecular species will escape a planetary atmosphere at different temperatures. These temperatures vary by thousands of degrees, and showcases how different species may be ripped away layer-by-layer as the tip of the AGB is approached.

*Parametrizing escape.* If the atmospheric loss is great enough, then the atmosphere will change structure. Assume that the SB is all atmosphere. Villaver & Livio [52] defined a helpful dimensionless quantity

6.8 $$\Gamma \equiv \frac{GM_{\mathrm{SB}}M_{H}}{k_{B}R_{\mathrm{SB}}T_{\mathrm{ex}}},$$

where *T*_ex_ is the temperature of the exosphere (which may be different from the SB’s effective temperature), *M*_H_ is the mass of a hydrogen atom and *k*_B_ is the Boltzmann constant. The quantity *Γ* represents the ratio of the gravitational potential energy to the particle kinetic energy, or the square of the ratio of the atmospheric escape velocity to thermal velocity. Villaver & Livio [52] found that the atmospheric structure is maintained for Γ≳20.

Determining *T*_ex_ depends on how one models heat transfer within an SB atmosphere. This transfer is determined by a combination of convection and conduction. Villaver & Livio [52] neglected convection because SBs orbiting GB stars are unlikely to be tidally synchronized. Consequently, they solved a conduction-dominated energy equation (their eqns 6 and 8) in order to obtain an expression which can be implicitly solved for *T*_ex_ (their eqn 7) given a wide variety of thermal properties (conductivity, thermospheric base temperature, intensity reaching atmosphere).

*Quantifying escape—number density flux* If the atmospheric structure is maintained (Γ≳20), then a lower limit to the escape rate by thermal evaporation can be expressed through a number flux *Φ* as (eqn 9 of [52])

6.9 $$\Phi =-\sqrt{\frac{k_{B}T_{\mathrm{ex}}}{2\pi M_{H}}}\delta (1+\Gamma )\;\exp(-\Gamma ),$$

where *δ* is the number density of the escaping constituent. The restriction that Γ≳20 limits the use of equation (6.9). A more complete treatment requires considering potential feedback factors, ram pressure stripping, photodissociation, immersion into a postshocked hot bubble, and the solution of the atmospheric structure equations; estimating the result with a single explicit expression like their eqn 10 still requires many assumptions that rely on results of numerical simulations anyway. Also, as suggested by Spiegel & Madhusudhan [131], extant pebbles and boulders could be accreted onto the SB’s atmosphere during post-MS evolution due to *N*-body interactions (see §7), altering the composition and structure of the atmosphere.

*Quantifying escape—mass flux.* With the same functional form of equation (6.9), Livio & Soker [248] provided an expression (in their eqn 6) for SB atmospheric mass loss while in the midst of a red giant. More recent treatments have also been provided [43,129]. Bear & Soker [43] incorporated both evaporation and ionization of the evaporated gas, and pointed out that recombination of the ionized gas is not necessarily an important process for MS exosystems, unlike in post-MS exosystems. By manipulating and rewriting their eqns 7 and 10, as well as assigning a negative sign for mass loss, I obtain

6.10 $$\frac{dM_{\mathrm{SB}}}{dt}\approx -\frac{1}{2}\left[\frac{2\pi GM_{\mathrm{SB}}v_{\mathrm{out}}^{2}}{\eta \Pi R}+\sqrt{\frac{4\pi^{2}G^{2}M_{\mathrm{SB}}^{2}v_{\mathrm{out}}^{4}}{\eta^{2}\Pi^{2}R^{2}}+\frac{8\pi R_{\mathrm{SB}}v_{\mathrm{out}}^{2}{\dot{H}}_{\mathrm{EUV}}}{\Pi R}}\right],$$

where *v*_out_ is the outflow speed, *η* is the efficiency factor for channeling EUV radiation to evaporation, *Π* is the average energy of ionizing photons, R is a recombination rate coefficient and ${\dot{H}}_{\mathrm{EUV}}$ is the total EUV power from a given wavelength range. Some characteristic values might be *v*_out_=2×10^5^ m s^−1^, *η*=0.2, *Π*=20 eV, $R=5\times 10^{34}\;m^{3}\;{\mathrm{kg}}^{-2}\;s^{-1}$ and ${\dot{H}}_{\mathrm{EUV}}=4\times 10^{23}\;J\;s^{-1}$.

*Giant planet atmospheres—chemical changes.* Campbell *et al.* [52] and Spiegel & Madhusudhan [131] are important studies because they speak directly to the fate of the giant planets in our Solar system and, in particular, Jupiter. We know that Jupiter will survive Solar mass loss and not undergo scattering instability, but will it evaporate completely? Section 4 of [131] is devoted to the chemical changes Jupiter will undergo during the Sun’s post-MS evolution, as well as what Jupiter’s spectrum would look like (their fig. 6). This spectrum includes some abundance alterations in carbon, methane and water.

### 6.2 Compact object radiation

#### 6.2.1 White dwarf radiation

*Comet sublimation* WD radiation causes an SB to lose mass, particularly if the SB is volatile rich. This mass loss may arise in the form of sublimation or outgassing. Veras *et al.* [254] made a distinction between the two terms, referring to the former as the homogeneous mass exodus of surface particles and the latter as localized violent eruptions. In either case, from equations (4.3)–(4.9), one can see that whatever mass is lost from the system by the SB will negligibly change its orbit. Instead, the largest orbital change will come from the kick due to mass loss-induced linear momentum recoil. Veras *et al.* [254] did not treat the kick as impulsive. They assumed the SB was continuously losing mass in a manner physically motivated by Solar system comets.

By assuming that an SB has a volatile content *M*^(vola)^_SB_ which is subject to sublimation and is made of a single species with molecular or atomic mass *M*_vola_, they found that the equations of motion become (their eqn 6)

6.11 $$\frac{d^{2}\text{r}}{dt^{2}}=-\frac{G(M_{⋆}+M_{\mathrm{SB}})}{r^{3}}\text{r}-\{H(M_{\mathrm{SB}}^{\left(\mathrm{vola}\right)})\}\frac{D_{0}v_{0}M_{\mathrm{vola}}}{M_{\mathrm{SB}}-M_{\mathrm{SB}}^{\left(\mathrm{vola}\right)}}{\left(\frac{r_{0}}{r}\right)}^{9/4}\frac{\text{r}}{r},$$

where H is the Heaviside function and *D*_0_, *v*_0_ and *r*_0_ are fiducial values representing, respectively, the number of molecules or atoms of the volatile species sublimating per time, the mean ejection velocity of that species, and distance to the WD.

Using this formulation, Veras *et al.* [254] found that for nearly parabolic orbits (e≳0.998)—as expected of SBs which pollute WDs—the orbital pericentre robustly resists changes to sublimative forces even though other orbital parameters might easily change. They generalized this result to arbitrary integral power-law dependencies on distance (their appendix A). Therefore, comets whose orbital pericentre is outside of the WD disruption sphere will not self-perturb themselves into the disruption sphere unless acted on by other agents.

Stone *et al.* [67], using a different set of assumptions than Veras *et al.* [254], approximated the location, *r*_sub_ at which a volatile-rich SB completely sublimates. By generalizing eqn 12 of [67], I obtain

6.12 $$r_{\mathrm{sub}}∼\frac{9L_{⋆}^{2}}{64GM_{⋆}R_{\mathrm{SB}}^{2}\rho_{\mathrm{SB}}^{2}T_{\mathrm{SB}}^{2}},$$

where $T_{\mathrm{SB}}$ is the SB’s latent heat of transformation (given as 3×10^10^ erg s^−1^) and is claimed to be similar for ice and silicates. If instead the comet partially sublimates at each pericentre passage, and the amount of mass lost per passage decreases steeply enough, then the total mass lost can be approximated by an converging sum as in eqn C2 of [67].

Stone *et al.* [67] also, in their footnote #4, estimates the terminal speed of ice ejected to be

6.13 $$v_{\mathrm{term}}=\sqrt{\frac{14k_{B}T_{\mathrm{ice}}}{27M_{H}},}$$

where *k*_B_ is the Boltzmann constant, *T*_ice_ is the sublimation temperature of ice and *M*_H_ is the mass of a hydrogen atom. They commented that the ejection velocity for dust is highly model-dependent (and hence more unconstrained than ice). They also discussed and quantified the potential distribution of debris left over from cometary sublimation (see their sections 3.2–3.3). These distributions rely on the specific orbital energy of the sublimating debris.

*Drag on substellar bodies.* Besides mass loss, the pressure of WD radiation on SBs will cause them to drift inward according to equation (6.1). Belts or rings with non-uniform size distributions might then respond to WD radiation in complex ways. I discuss the implications of this process in the context of forming WD discs from first-generation SBs in §9.

*Sublimating white dwarf debris discs.* WD radiation plays a crucial role in the morphology and evolution of WD debris discs. This radiation sublimates dust into gas and mingles the two. I discuss the implications of this sublimation in §11.

*Effects on substellar body atmospheres.* The relatively small size and quickly dimming nature of WDs provide for interesting SB atmospheric dynamics. In contrast with the extreme MS planet Kepler-91b, which is so close to its parent star that 70% of the planet atmosphere is illuminated by the host star (fig. 10 of [10]), in WD systems much less than 50% of a giant planet atmosphere would be illuminated (the radius of a WD is an order of magnitude smaller than that of a giant planet). I summarize SB atmospheric studies from WD systems in §12.4 in the context of climate and habitability.

#### 6.2.2 Pulsar radiation

The radiation emitted from pulsars contains highly energetic and relativistic particles. The consequences for SBs are different than in MS, GB and WD planetary systems. Also, processes like ohmic dissipation, or induction heating (see Appendix B of [194]), might not be as prevalent in MS, GB or WD systems. Further, pulsar radiation evaporates and ionizes infalling bodies. When remaining charges are accelerated to relativistic energies, they can interact with the magnetic field to produce observable signatures [193].

*Implications for second-generation formation.* In the context of second-generation formation, relativistic particles can break apart complex nuclei and prevent metals from forming [73]. Therefore, shielding from particle radiation may be necessary to allow planet formation for a high-enough luminosity. Miller & Hamilton [73] considered how the luminosity of a pulsar changes as it is being spun-up, with implications for second-generation planet formation (see §8).

*Implications for formed substellar bodies.* The putative asteroid disc that orbits PSR B1937+21 might be composed of many asteroid-sized objects on non-crossing orbits that range down to a few tenths of an AU [186]. Consequently, the Yarkovsky effect, driven by the magnetosphere from beamed X-rays, might play a significant role in the evolution of this disc. They adopted the formalism of Cordes & Shannon [194] and find that only SBs with $R_{\mathrm{SB}}≳5\;\mathrm{km}$ could survive infall due to the Yarkovsky effect if in the nascent disc a≳1 AU. This estimation provides a strong constraint on the potential present-day size distribution in the disc.

## 7. Multi-body interactions

The forces I described in §§4–6 involve the star and SB only: effectively, the two-body problem. Now I consider the *N*-body problem, where *N*≥3 and one of the bodies is the parent star. Because the three-body problem is generally unsolvable, *N*-body interactions—particularly in post-MS systems with so many additional forces involved—are often modelled with numerical simulations, a prominent theme in this section. The actual computer codes used by the authors are not mentioned here, but rather in a dedicated section (§14) for easier reference.

A single *N*-body numerical integration of a chaotic system is not a deterministic prediction. Fig. 1b of [255] illustrates the maximum eccentricity that the planet Mercury will achieve over the next 5 Gyr: this value can vary by nearly unity for two simulations with equivalent initial conditions except for a variation of 0.38 mm in Mercury’s initial semi-major axis. The authors refer to the search of a precise solution over this interval as ‘hopeless’.

Although the long-term MS orbital evolution of planets and planetesimals has been studied extensively (see [256] for a review), rarely have previous investigations carried out numerical integrations for the entire duration of the MS. Consequently, the initial orbital architectures for post-MS studies are poorly understood, much less the subsequent multi-body interactions with the complexities introduced by GB evolution.

*Common simulation features.* A common assumption is that all SBs change their orbits simultaneously due to stellar mass loss. In reality, there is an ejecta-lag crossing time. The orbit of the closest SB will shift before the orbits of any other SBs. The consequences of this assumption have not yet been explored in detail, but are likely to be greatest, as pointed out by Veras *et al.* [24], (i) for widely spaced orbits, and/or (ii) for non-adiabatic mass loss dynamics. According to observations [33], ejecta may take on the order of 100 days to travel just 1 AU.

Also, simulations which feature mass loss do not conserve energy. Many, however, do conserve angular momentum. Angular momentum conservation then provides an important check on the accuracy of simulations and a check on the physics. Bear *et al.* [41] demonstrated how conservation of angular momentum arguments alone can help exclude additional bodies from playing a significant dynamical role (or existing at all) during the history of a system.

In the common case of SBs experiencing adiabatic motion due to stellar mass loss, while the system is stable, eccentricity variations can be attributed primarily to their mutual interactions rather than from mass loss. The true anomaly and eccentric anomaly evolution is also negligibly affected by mass loss. These attributes are useful for disentangling the effects of mutual interactions and mass loss.

### 7.1 Collisions within debris discs

Given the variety of forces affecting substellar bodies in post-MS systems, debris discs may geometrically represent ‘discs’ only in a loose sense. Nevertheless, for the purposes of this section, assume that the disc can be represented by an annulus with inner and outer boundaries represented by *r*^(min)^_d_ and *r*^(max)^_d_, where *r* is exactly in-between those two values.

If mutual velocities are sufficiently high for collisions to be destructive, then collisional erosion continuously replenishes smallest bodies. Therefore, the collisional lifetimes of bodies of different sizes are key parameters. In general, the collisional lifetime, *t*_coll_, for particles of a given size is provided by eqn 7 of [257] as

7.1 $$t_{\mathrm{coll}}=\frac{2\langle i\rangle r^{5/2}(r_{d}^{\left(\max\right)}-r_{d}^{\left(\min\right)})}{\sqrt{G}M_{⋆}^{1/2}\Sigma_{d}\xi b}$$

with *Σ*_d_ representing the cross-sectional area, *ξ* representing the ratio of the relative collisional velocity to the Keplerian velocity and *b* representing the fraction of the disc’s cross-sectional area in which catastrophic collisions with these large objects may occur. Generally, *t*_coll_ decreases as the disc approaches the central star. The shorter the collisional lifetime, the more quickly collisional equilibrium is reached. In this case, memory of the initial mass is erased. However, collisional equilibrium might not hold for all evolved discs [258].

Various specific cases of equation (7.1) have been used in the subsequent literature. Among the most relevant for post-MS evolution is contained within the study by Bonsor & Wyatt [32]. They find that the disc mass evolves according to (their eqns 20 and 21)

7.2 $$M_{d}^{\left(\mathrm{GB}\right)}=M_{d}^{\left(\mathrm{MS}\right)}{\left[1+CM_{d}^{\left(\mathrm{MS}\right)}\int M_{⋆}^{17/3}\;dt\right]}^{-1},$$

where

**Figure 10.**
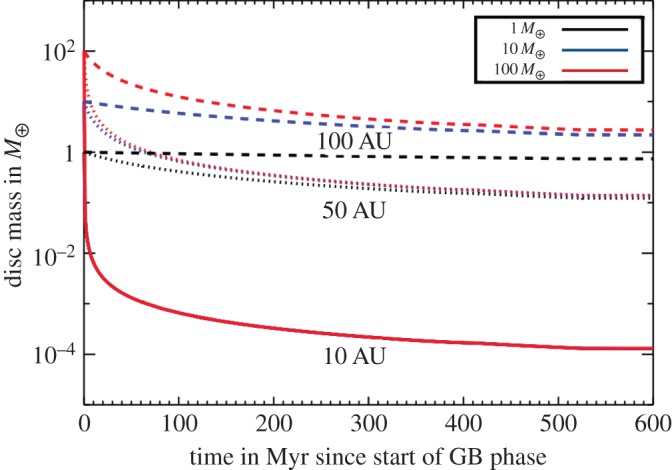
Cosmetically enhanced version of the lowest panel of fig. 4 of [32]. How GB evolution of a $M_{⋆}^{\left(\mathrm{MS}\right)}=2.9\;M_{⊙}$ star depletes a debris disc due to collisional evolution alone. The legend indicates the initial disc masses. The solid lines correspond to $\left\{r=10\;\mathrm{AU},r_{d}^{\left(\min\right)}=7.5\;\mathrm{AU},\;r_{d}^{\left(\max\right)}=12.5\;\mathrm{AU}\right\}$, the dotted lines correspond to $\left\{r=50\;\mathrm{AU},\;r_{d}^{\left(\min\right)}=37.5\;\mathrm{AU},\;r_{d}^{\left(\max\right)}=62.5\;\mathrm{AU}\right\}$ and the dashed lines correspond to $\left\{r=100\;\mathrm{AU},\;r_{d}^{\left(\min\right)}=75\;\mathrm{AU},\;r_{d}^{\left(\max\right)}=125\;\mathrm{AU}\right\}$.

7.3 $$C\equiv {\left[2.8\times 10^{-9}\left(\frac{r_{d}^{\left(\max\right)}-r_{d}^{\left(\min\right)}}{r}\right)e^{-5/3}\;\text{max}(R_{\mathrm{SB}})Q^{5/6}{r^{\left(\mathrm{MS}\right)}}^{13/3}{M_{⋆}^{\left(\mathrm{MS}\right)}}^{13/3}\right]}^{-1}$$

and Q is the dispersal threshold for collisions. Figure 10 illustrates how disc mass decreases with time according to equation (7.2).

### 7.2 One star, one planet and asteroids

At least five investigations so far [59,61,227,259,260] have modelled the post-MS interactions between one planet and a collection or sometimes belt of smaller objects with numerical simulations.

#### 7.2.1 A Kuiper belt with a Neptune

Bonsor *et al.* [259] modelled a 1 *M*_⊙_ MS star that loses $\frac{2}{3}$ of its mass at a constant rate (6.7×10^−6^ *M*_⊙_ yr^−1^) over 10^5^ yr. Because of the constant mass loss rate assumption, stellar metallicity is not taken into account. They placed a Neptune-mass planet initially at 30 AU in a circular orbit, with 500 test particles with initial semi-major axes from 30 to 47.6 AU such that their initial eccentricities and inclinations were less than 0.1 and 10 degrees, respectively. In effect, this setup represents a cold exo-Kuiper belt. Given the distances involved, stellar mass loss is adiabatic in all their systems.

In order to develop initial conditions for the post-MS simulations, they integrated these systems for 10^7^ years with a static parent star (along the MS) to scatter away dynamically fragile members and settle the belt. This timescale is typically three orders of magnitude smaller than MS lifetimes, and was adopted to effectively create a largely dynamically settled exo-Kuiper belt.

The simulations demonstrate that changing stability boundaries from mass loss allow a fraction of the belt to be scattered inwards, creating an inner reservoir of material after the star has become a WD. If additional planets on circular orbits exist inward of the Neptune, then subsequent scattering with the test particles can be constrained analytically, and allow these particles to achieve WD-grazing orbits [261].

#### 7.2.2 An asteroid belt with a Jupiter

Debes *et al.* [59] simulated the orbital evolution of one planet with the physical and orbital properties of Jupiter, along with 710 interior-lying asteroids with orbital properties equivalent to Solar system asteroids. The parent star contained 1 *M*_⊙_ on the MS, and lost 0.46 *M*_⊙_ over 1000 years according to a prescription from Schrder & Smith [230], corresponding to an average mass loss rate of 4.6×10^−4^ *M*_⊙_ yr^−1^. Ten simulations were run for 100 Myr, four for 200 Myr and three for 1 Gyr. Their simulations tracked test particles until they reached 1*R*_⊙_, upon which they were removed.

**Figure 11.**
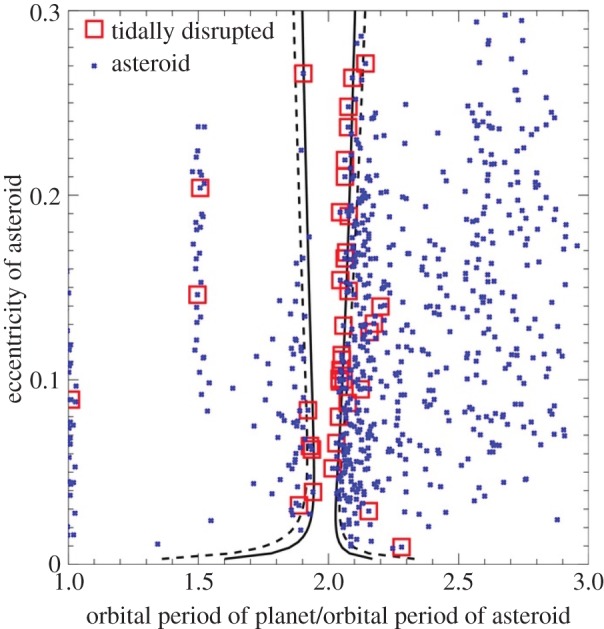
Cosmetically enhanced version of fig. 2 of [59]. How the GB-induced expansion of the libration width of the 2 : 1 mean motion resonance between a planet and asteroids induces the latter to be scattered towards and inside of the WD disruption radius. The solid and dashed lines are the libration widths for, respectively, $M_{⋆}^{\left(\mathrm{MS}\right)}=1.0\;M_{⊙}$ and $M_{⋆}^{\left(\mathrm{WD}\right)}=0.5\;M_{⊙}$. Note that a couple asteroids near the 3 : 2 mean motion commensurability are also scattered towards the WD and disrupted.

Debes *et al.* [59] also emphasized the importance of resonant interactions between a planet and test particle. They showed how the libration width of the interior 2:1 mean motion resonance grows during mass loss, capturing test particles (figure 11). The eccentricity of these particles then increase until they potentially enter the WD disruption distance. Over all their simulations, about 2% of test particles followed this path; just one asteroid per simulation disrupted after 200 Myr. They found that in order to match WD observations on aggregate, exo-asteroid belts would need to be between 4×10^0^ and 6×10^5^ as massive as the Solar system asteroid belt. The large range perhaps emphasizes that a single model has difficulty explaining the entire population of polluted WDs.

Frewen & Hansen [61] explored a wider region of phase space than Debes *et al.* [59] through a series of separate MS, GB and WD numerical simulations. The MS simulations were run for 0.1 Gyr (about two orders of magnitude less than a typical MS lifetime), the GB simulations were run for 2700 yr and the WD simulations were run for 1.0 Gyr. Among these simulations the authors adopted planet masses of 0.03,0.3,1.0 and 4.0*M*_Jup_ and eccentricities of 0.02,0.2,0.4,0.6 and 0.8 (one planet per simulation). In every case, the planet’s MS and resulting WD semi-major axes were 4 and 7.42 AU, as the 1.0 *M*_⊙_ MS parent star was modelled to lose a total of 0.461 *M*_⊙_, and at a constant (1.7×10^−4^ *M*_⊙_ yr^−1^) rate. The particle belt surrounded the planet in order to achieve coverage of many first- and second-order internal and external mean motion commensurability locations. They discussed the potential importance of the effects of wind drag and Yarkovsky forces (their section 5.1), but did not include them in simulations. These simulations importantly demonstrated that higher planetary eccentricities and smaller masses enhance the accretion rate.

#### 7.2.3 Other configurations

Dong *et al.* [227] performed a set of simulations with a single planet and a few planetesimals (see their figs 3–5). The planet mass was a Jupiter-mass, and the planet was initially placed on a circular orbit at 20 AU. Planetesimals of different sizes were placed on circular orbits at 35 AU, and the 4 *M*_⊙_ parent star was made to lose mass linearly at a rate of 3×10^−5^ *M*_⊙_ yr^−1^ for 10^5^ yr. Unlike the above four studies [59,61,259,260], wind drag (see §4.3) was also included in the simulations from Dong *et al.* [227]. They illustrated that in this setup, capture into the external 3 : 2 mean motion resonance occurs.

### 7.3 One star, multiple planets and no asteroids

#### 7.3.1 Few-planet stability boundary changes

*Two planets*. The discovery of the post-MS PSR B1257+12 planets in 1992 [1] provoked a resurgence of interest in celestial mechanics and in particular the three-body problem. The following year, Gladman [262] conveyed a concept from algebraic topology—that of Hill stability (in a Hill stable system, two planetary orbits never cross)—to the wider astronomical audience by expressing the relevant criterion in orbital elements and in the context of two coplanar small planets orbiting a star. A decade later, Debes & Sigurdsson [211] then illustrated how in post-MS systems, mass loss can convert a Hill stable system into an unstable one. A decade after that, Veras *et al.* [24] considered this transition to Hill instability with arbitrarily eccentric and inclined orbits. However, mounting progress in characterizing instability in the few-body problem (see, e.g. [263] for a review) is not limited to Hill stability. Several authors have since popularized Lidov–Kozai motions, gave attention to Lagrange instability (a proxy for the global stability boundary), and generally provided an appreciation for orbital architectures previously assumed to be unorthodox and perhaps ignorable (see [256] for a review).

*Three planets.* For systems with more than two planets, Hill stability no longer applies. Instead, authors have introduced the concept of a critical separation for instability where a single relation links the consecutive pairwise planet separations. Mustill *et al.* [264] derived the following estimate for the critical separation *Δ* (adapted from their eqns 2 and 6) assuming three equal-mass planets

7.4 $$\begin{array}{rl} \Delta & \approx 0.7a_{\mathrm{inner}}{\left(\frac{M_{\mathrm{SB}}}{3\;M_{⊙}}\right)}^{1/3}{\left(\frac{M_{\mathrm{SB}}}{M_{\mathrm{Jup}}}\right)}^{-1/12}{\left(\frac{M_{⋆}^{\left(i\right)}}{M_{⊙}}\right)}^{1/12}{\left(\frac{M_{⋆}^{\left(f\right)}}{M_{⋆}^{\left(i\right)}}\right)}^{-1/4}\\ & \;\times \left[\log\frac{t_{f}-t_{i}}{\mathrm{Myr}}-\frac{1}{2}\log\frac{M_{⋆}^{\left(i\right)}}{M_{⊙}}+\log\frac{M_{\mathrm{SB}}}{M_{\mathrm{Jup}}}-\frac{3}{2}\log\frac{a}{\mathrm{AU}}+\log\frac{M_{⋆}^{\left(f\right)}}{M_{⋆}^{\left(i\right)}}+11.0\right], \end{array}$$

where the superscripts i and f refer to initial and final states, and not necessarily MS and WD. This relation helps show that higher mass stars produce greater amounts of instability because the planet–star mass ratio becomes more unequal.

*More than three planets.* For more than three planets, Duncan & Lissauer [223] found a roughly linear relationship between instability time and amount of stellar mass lost. Veras & Gänsicke [144] quantified the initial separations for which the planetary system would significantly alter its initial configuration (or become unstable, by some definitions). They adopted the mutual separation distance formulation (see their eqn 1) from Smith & Lissauer [265], which is measured in number of ‘mutual Hill radii’, denoted by *β*. Veras & Gänsicke [144] found that closely packed giant planets with *β*≈10–12 typically unpack or become unstable during the post-MS phases. For terrestrial planets, instead *β*≈6.

#### 7.3.2 GB and white dwarf planet simulations

*Two planets.* In 2002, Debes & Sigurdsson [211] demonstrated that adiabatic mass loss changes the stability boundary in multi-planet systems, and can induce instability in previously stable systems. They illustrated that although the ratio of semi-major axes of two planets remained unchanged through the GB phases, the critical Hill stability separation does not scale in the same way. Consequently, during GB mass loss, two planets can cross this boundary. They performed numerical simulations which involved increasing the mass of the planets rather than decreasing the mass of the star. The planet mass increase corresponded to a halving of the stellar mass over 10^5^ orbits of the inner planet; the simulations were run for up to 10^7^ of these orbits. The authors adopted planet–star mass ratios of 10^−7^, 10^−5^ and 10^−3^.

The complementary work of Voyatzis *et al.* [215] a decade later explored how non-adiabatic mass loss changes stability in two-planet systems. For real systems with stars that become WDs, this situation would arise only for two SBs which are at least several hundred AU away from the GB star. They found that the resulting changes in eccentricity enhance the prospects for instability. One way of tracing the susceptibility of a two-planet system to become unstable over a Hubble time (or current age of the Universe) is to track the Lyapunov exponent [200,215]. Adams *et al.* [200] found that although the Lyapunov exponent varies by orders of magnitude depending on the mass loss timescale, both timescales are comparable.

**Figure 12.**
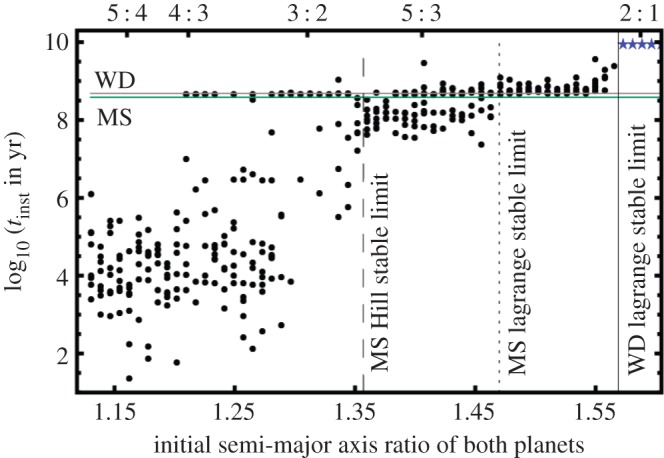
Cosmetically enhanced version of the bottom-rightmost panel of fig. 9 of [24]. I show instability timescales, *t*_inst_, as black dots for individual two-planet simulations with the given initial semi-major axis ratio and all with a parent star of $M_{⋆}^{\left(\mathrm{MS}\right)}=3.0\;M_{⊙}$. Blue stars indicate that all simulations sampled at that particular semi-major axis ratio were stable over a total of 5 Gyr of evolution. The two coloured horizontal lines represent the RGB and AGB phases, and the upper axis illustrates some mean motion commensurabilities. This plot demonstrates that (i) systems which are stable on the MS may become unstable beyond the MS and (ii) that Lagrange instability on the MS can manifest itself late, right before the RGB phase.

Motivated by instability induced by crossing the Hill stable boundary, Veras *et al.* [24] performed numerical integrations incorporating both planetary and stellar evolution for two planets across all post-formation phases of evolution. They adopted *a*_inner_=10 AU, $M_{⋆}^{\left(\mathrm{MS}\right)}=[3,4,5,6,7,8]M_{⊙}$, and ran simulations for 5 Gyr, a long enough period to cover the MS and GB phases and several Gyr on the WD phase (see their fig. 7). They primarily adopted initially coplanar planets with Jovian masses, eccentricities of 0.1 and initial separations that cover a range of possible stability outcomes (see fig. 12 of this paper).

Their results demonstrated that (i) post-MS mass loss alone can trigger instability not only during the GB phase, but well after. In fact, because in almost every case the mass loss is adiabatic, the architecture of the system inflates without a notable initial change. But that process in effect ‘resets’ the system, so that subsequent dynamical instability occurs on timescales similar to those one might expect after formation on the MS (figure 12). (ii) The ejection of one planet perturbs the other onto a moderately to highly eccentric orbit, sometimes with an orbital pericentre of just a few AU. This eccentric planet could then perturb any interior asteroids (not modelled), providing a dynamical environment for pollution. Due to conservation of angular momentum, the semi-major axis of the surviving planet is well-approximated analytically (see their eqn 8 and figs 14 and 16), unlike its eccentricity, which can be determined only if the hyperbolic eccentricity and semi-major axis of the escaping planet are known.

*Three planets.* In a follow-up paper that integrated systems with three Jovian planets instead of two, Mustill *et al.* [264] improved upon Veras *et al.* [24] in several respects, including: (i) they explored multi-generational instability. For example, with three planets, one instability can occur on the MS, with the second on the WD (see their fig. 5). In about 1% of all cases, all three planets were lost. (ii) They more accurately quantified the fraction of unstable post-MS systems which lead to direct planet–WD collisions by adopting the WD disruption radius instead of the actual WD radius, and by giving the three planets slightly non-coplanar inclinations (in line with Solar system observations). They found that, like in the two-planet case, ejection during the WD phase is the most commonly occurring type of instability (see their fig. 4). These ejected planets help ensure that WD planets rarely evolve to a hot Jupiter state, and provide a potentially non-negligible contribution [266] to the purported free-floating planet population [267].

**Figure 13.**
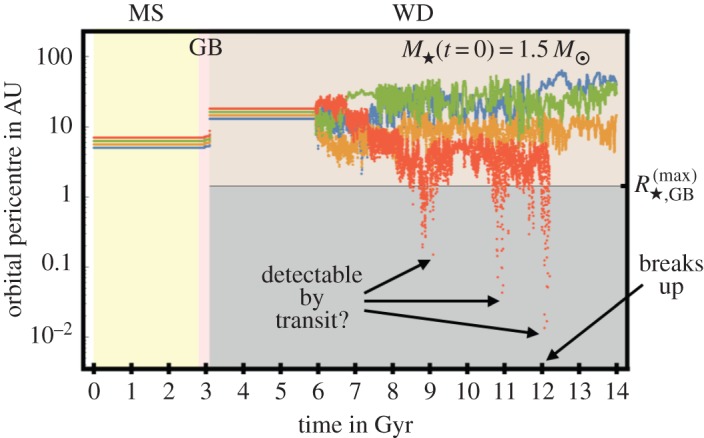
Cosmetically enhanced version of the upper-left panel of fig. 1 of [144]. Late unpacking of four tightly-packed terrestrial planets throughout the MS and GB phases. The orbit meandering which follows scattering instability perturbs the red planet into a likely transit-detectable orbit before it enters the WD disruption radius.

*More than three planets.* Veras & Gänsicke [144] then extended earlier work [24,264] by adopting $M_{⋆}^{\left(\mathrm{MS}\right)}=1.5-2.5\;M_{⊙}$, which corresponds to the primary source of progenitors of the currently observed WDs (figure 3), and by considering the evolution of packed systems with four and ten planets. They also integrated these systems for the current age of the Universe, 14 Gyr, and focused on lower-mass, terrestrial planets. As figure 13 demonstrates, these packed, stable and quiescent terrestrial planets on the MS can remain so throughout the GB phase, and for several Gyr along the WD phase, before ‘unpacking’. Subsequent to unpacking, the planets may meander and approach the WD closely enough for tidal circularization to occur, increasing prospects for transit detectability by decreasing the orbital period. Interactions between these terrestrial planets were not strong enough to cause escape in any of their simulations, unlike for the giant planet case, representing a fundamental difference between their earlier studies ([24] and [264]).

*More than three planets plus moons.* The presence of exo-moons in WD systems can provide a potentially important mass reservoir for heavy metal pollution. In our Solar system, the total mass of moons exceeds that of the asteroid belt by over two orders of magnitude. Using the data from the simulations of Veras & Gnsicke [144], Payne *et al.* [268] considered how susceptible moons are to planet–planet scattering which occurs during the WD phase. They found that after adiabatic mass loss, the Hill radius *r*_*Hill*_ of a planet increases according to (their eqn 7):

7.5 $$\frac{r_{\mathrm{Hill}}^{\left(\mathrm{WD}\right)}}{r_{\mathrm{Hill}}^{\left(\mathrm{MS}\right)}}={\left(\frac{M_{⋆}^{\left(\mathrm{MS}\right)}}{M_{⋆}^{\left(\mathrm{WD}\right)}}\right)}^{4/3}.$$

Nevertheless, even though moons become more entrenched, and hence stable, after post-MS mass loss, Payne *et al.* [268] showed that planet–planet scattering on the WD phase is highly destructive to moons. They demonstrated that moons may be regularly liberated from their parent planets. Although the ultimate fate of the moons was not explicitly tracked, the moons may subsequently be perturbed into WDs directly or act as agents to perturb smaller bodies, fragments or dust into WDs.

One earlier work [223] performed a variety of multi-planet simulations with different planet–star mass scalings which mirror those measured in the Solar system. In some cases they treated mass loss as constant, and in others used a fitted, observationally-motivated prescription. The latter case helps demonstrate that the four outer Solar system planets are likely to survive post-MS mass loss and remain stable at least for tens of Gyr (see §13).

#### 7.3.3 Pulsar planet simulations

The exquisite data available for PSR B1257+12 [269] (with e.g. eccentricity accuracy at the 10^−4^ level) represents an opportunity to study the dynamics of a multi-planet system at a standard of detail that is largely unattainable in MS planetary systems. Gozdźiewski *et al.* [270] performed an extensive analysis of the system with 1 Gyr numerical integrations, stability maps and Lyapunov exponent analyses. They found (i) the system is long-term stable, (ii) none of the planets experience (notable) secular variations in semi-major axis, eccentricity or inclination, (iii) the outermost two planets are locked in a secular apsidal resonance with a libration centre of 180° and (iv) the line of nodes of the inner planet must be within 60 degrees of the nodes of the outer planets.

### 7.4 Two stars, planets and no asteroids

Simulations for this physical setup cover a wide range of physical architectures and numerical codes. Here, I divide previous investigations according to whether or not the numerical codes adopted model stellar evolution.

#### 7.4.1 Simulations not incorporating stellar evolution

*PSR B1620-26* The fascinating PSR B1620-26 system contains a millisecond pulsar (PSR B1620-26A), a WD (PSR B1620-26B) and a circumbinary planet (PSR B1620-26AB b) [135]. The dynamical pathway leading to this scenario is thought to proceed along the following lines: (i) the planet forms around the MS progenitor of the currently-observed WD, (ii) that MS star-planet system flies by a NS and its original unknown companion within the M4 globular cluster, (iii) this flyby induces an exchange reaction, where the NS’s companion is ejected and the NS captures the star and planet, (iv) the planet begins orbiting both stars in a circumbinary fashion, (v) the MS star evolves into a GB star, (vi) mass transfer from the GB onto the NS spins the latter up and converts it into a millisecond pulsar and (vii) the GB star evolves into a WD. The result is the currently observed system.

The third part of this scenario was simulated with four-body dynamics [133], and remains an accurate qualitative description of the exchange despite the now-outdated orbital parameters used. This exchange occurs too quickly for stellar evolution to play a role.

*Post-CE binaries.* The future dynamical stability of the putative post-CE binary planetary systems (§3.4) have been explored with simulations in many studies: for HU Aquarii (HU Aqr) [271,272]; for QS Virginis (QS Vir) [273]; for NSVS 14256825 [274]; for NN Serpentis (NN Ser) [275]. The overall conclusions from these investigations is that all systems except for NN Ser undergo instability on short (approx. 10^3^−10^4^ years) timescales, placing doubt on the planetary interpretation of the signals. However, a few isolated long-term stable solutions also do exist for HU Aqr. Marsh *et al.* [175] criticized the above studies by claiming that the initial conditions for their simulations are not consistent with the data, as the strong correlations between the orbital parameters were not taken into account. They do not necessarily believe that the qualitative stability conclusions of these other papers would change as a result, but do think that a re-evaluation is needed to be sure.

#### 7.4.2 Simulations incorporating stellar evolution

The intriguing dynamics of post-MS evolution in binary systems which hosts planets has been explored with stellar evolution simulations by only a handful of investigations. The first two presented here [176,276] took alternate approaches to studying two of the post-CE binary systems from the last section: NN Ser and HU Aqu. The third [277] is an altogether different type of study that considered how a planet can ‘hop’ from one star to another, and the fourth [278] quantified where circumbinary planets are subject to escape.

*NN Ser.* For the purposes of investigating the stability of NN Ser, Mustill *et al.* [176] took an alternate approach from Horner *et al.* [275] by looking at its past rather than its future. If the putative planets could not maintain stable orbits on the MS, then the current observations are of either second-generation planets, or of other phenomena entirely. Through the GB phases, the planets’ orbits increase due to adiabatic mass loss (see §4) while the stellar binary orbit shrinks because star–star tides dominate the evolution of their mutual orbit (see their fig. 1). Therefore, the MS stability of this system is more precarious than after the CE because $a_{\mathrm{inner}}^{\left(\mathrm{MS}\right)}<a_{\mathrm{inner}}^{\left(\mathrm{GB}\right)}$, $a_{\mathrm{outer}}^{\left(\mathrm{MS}\right)}<a_{\mathrm{outer}}^{\left(\mathrm{GB}\right)}$ and $a_{b}^{\left(\mathrm{MS}\right)}>a_{b}^{\left(\mathrm{GB}\right)}$.

To perform this investigation they (i) computed what MS orbital and physical parameters of the binary correspond with current observations by using a stellar evolution code, (ii) analytically determined, by assuming adiabatic mass loss and negligible other forces, the MS orbits of the putative planets and (iii), finally, integrated them along the MS with a planetary evolution code to assess their stability. They found that in nearly all (99.74%) cases the planets become unstable before the end of the MS of the higher mass star, suggesting that the currently observed planet candidates do not have a first-generation origin.

*HU Aqr.* The putative planets around the cataclysmic variable HU Aqr are less certain to exist because of the widespread instability found amongst the simulations performed [271,272]. Nevertheless, the existence of at least one long-term stable solution prompted Portegies Zwart [276] to perform a ‘reconstruction’ of the dynamical history of that system through its CE phase.

As a first step, Portegies Zwart [276] determined what physical and orbital binary system parameters reproduce the system seen today. Then, he integrated the planets’ orbits by making assumptions about the CE, such as the CE resulting in differing mass loss rates felt by both planets. An important outcome of that study is the ability to restrict CE parameters, assuming that the planets exist now in the post-CE phase. He found that the fraction of orbital energy needed to unbind the CE during inspiral was 45±17%.

*Planet hopping.* Kratter & Perets [277] explored the exciting possibility that an SB can unbind from its parent star due to stellar mass loss and then be captured by the stellar companion, performing a ‘hop’. This mechanism works because the change in the SB’s orbit is greater than the binary companion’s if the expansion of both are adiabatic (a realistic possibility for $a_{b}≲10^{3}\;\mathrm{AU}$). Suppose the binary companion is an MS star which remains as such throughout the primary star’s post-MS evolution. Suppose also that the primary star becomes a WD. Then, assuming full adiabaticity, *M*_SB_≪*M*_★_, and *M*_SB_≪*M*_b_, I obtain (see also eqn 6 of [279])

7.6 $$a_{b}^{\left(\mathrm{WD}\right)}=a_{b}^{\left(\mathrm{MS}\right)}\left(\frac{M_{⋆}^{\left(\mathrm{MS}\right)}+M_{b}}{M_{⋆}^{\left(\mathrm{WD}\right)}+M_{b}}\right)$$

and

7.7 $$a_{\mathrm{SB}}^{\left(\mathrm{WD}\right)}=a_{\mathrm{SB}}^{\left(\mathrm{MS}\right)}\left(\frac{M_{⋆}^{\left(\mathrm{MS}\right)}}{M_{⋆}^{\left(\mathrm{WD}\right)}}\right).$$

The other orbital parameters remain the same in the adiabatic limit (see §4.1). The differential orbital expansion of the binary and planetary orbit in addition to the mass change can (i) allow a stability threshold to be crossed, or, as explored by Bonsor & Veras [260], (ii) allow the binary orbit to be wide enough to incite future instability due to Galactic tides. If the binary orbital expansion in non-adiabatic, but the planetary expansion is adiabatic, then a previously stable system might remain so, depending on the effect of Galactic tides.

As explained further by Kratter & Perets [277] and visualized in their fig. 2, in order for the SB to be captured by the binary companion, the Jacobi constant must adhere to certain values. Although defined for the circular restricted three-body problem with fixed masses (including one massless particle), the Jacobi constant still represents a reliable proxy for capture in post-MS binaries with a single planet.

Kratter & Perets [277] performed simulations with 70 different binary configurations which include different stellar masses, binary separations in the range *a*^(MS)^_b_=75−105 AU and a fixed *a*^(MS)^_SB_=15 AU, *e*^(MS)^_b_=0.0 and *e*^(MS)^_SB_=0.0. They ran simulations for the entire duration of mass loss, plus 30 Myr. Their outputs show that about 10% of all SBs are captured by the companion, with typically prograde eccentric orbits satisfying *e*^(MS)^_SB_>0.4. Unstable systems typically featured a planet–star collision.

*Circumbinary escape* Veras & Tout [278] instead determined where in phase space will a circumbinary SB’s evolution become non-adiabatic (defined as *Ψ*>1; see equation (4.10)) and consequently in danger of escape due to post-MS evolution. They illustrated this critical SB semi-major axis in a series of contour plots, which explored dependencies on stellar masses (both $M_{⋆}^{\left(\mathrm{MS}\right)}$ and *M*^(MS)^_b_ ranging from 0.2 *M*_⊙_ to 8 *M*_⊙_), initial binary semi-major axis (*a*_b_=[10,50,100,500,1000,5000]*R*_⊙_), initial binary eccentricity (*e*_b_=[0.0,0.5,0.9]), stellar metallicity (*Z*_★_=*Z*_b_=[0.0001,0.0005,0.001,0.005,0.01,0.02]) and CE-blow off timescale ([10,10^2^,10^3^,10^4^] years). The SBs themselves were not included in these stellar evolution simulations.

Veras & Tout [278] found that the complex physics of binary star evolution, which include CEs and SNs, reduces the adiabatic limit significantly (to several tens of AU) from the single-star case (at several hundreds of AU). Therefore, the post-MS contribution to the free-floating exoplanet population [266] is expected to be higher for circumbinary rather than single star systems. Their study also backward-engineered the adiabaticity condition to yield a potentially useful constraint on stellar mass loss. Suppose an SB is bound to two MS stars and harbours an initial semi-major axis *a*^(MS)^_*CM*_ with respect to the centre of mass of the stars. Now the primary star becomes a WD. During that process, the maximum value of $|{\dot{M}}_{⋆}|$ which guarantees that the SB remains bound is (corrected from their eqn 6, which should contain an extra factor of *G*^1/2^; their eqn 4 is also missing a factor of *G*^−1/2^)

7.8 $$\max(-{\dot{M}}_{⋆})\approx \sqrt{G}{\left[\frac{1.4\;M_{⊙}+M_{b}}{\sqrt{a_{\mathrm{CM}}^{\left(\mathrm{MS}\right)}(M_{⋆}^{\left(\mathrm{MS}\right)}+M_{b})}}\right]}^{3}.$$

This result could be generalized to higher multiplicity cases or simplified to the single-star case (see their fig. 1).

### 7.5 Two stars, one planet and asteroids

Bonsor & Veras [260] considered a set-up with an exo-Kuiper belt and a planet orbiting one star of a wide stellar binary (*r*_b_∼10^3^−10^4^ AU). After the parent star has become a WD the two stars experience a close encounter due to Galactic tides (see §12.3 for more details). This encounter perturbs the planet, which is turn perturbs the asteroids into the WD, polluting it. They model a Neptune-mass planet on a circular orbit at 30 AU, and a belt of test particles from 30–50 AU. These simulations did not require the parent star to evolve because it was already a WD. The dynamics of the encounter between the secondary star, planet and belt reveals that the more intrusive the encounter, the higher the rate of inwardly scattered particles.

### 7.6 Three stars only

Although focused on the dynamics of hierarchical triple stellar systems with mass loss and without SBs, both Shappee & Thompson [62] and Michaely & Perets [280] derived results which may be applicable to SBs in the appropriate limits. Shappee & Thompson [62] modelled linear and adiabatic mass loss from a component of the inner binary with *N*-body integrations and considered the resulting change or induction of Lidov–Kozai behaviour. Michaely & Perets [280] instead adopted a Hamiltonian formalism, derived doubly averaged equations including mass loss up to octupole order and derived the secular equations of motion in the case when all mass loss is adiabatic. They also consider the general case when all three bodies lose mass. Secular equations allow for relatively quick integrations over long timescales, but by definition cannot resolve characteristics on orbital timescales.

## 8. Formation from stellar fallback

Some mass ejected by post-MS stars might not escape the system, and infalling material may be decelerated due to phenomena such as reverse shock waves [281]. Both WDs and NSs could host remnant discs of matter through which ‘second-generation’ SBs form. First-generation SB formation proceeds at timescales less than 10 Myr. If second-generation formation is similar, then we should not expect it to occur later than WD or NS cooling ages of 10 Myr. Consequently, because the vast majority of observations of remnant planetary systems (§3.1) are snapshots in time where *t*_cool_≫10 Myr, we are currently not observing activity from stellar fallback. Further, the chemical imprint of pollution observed within WD atmospheres is incongruous with the chemical composition of stellar mass ejecta.

Nevertheless, the prospect of second-generation formation of SBs at very early WD and NS cooling ages remains viable, and has been explored by researchers for decades. The PSR B1257+12 system was a strong driver for this research [282,283], and recent pulsar observations [186,193] provide further motivation. Incentive also arises from putative planetary systems whose first-generation provenance is disputed based on dynamical grounds (as in [25,176]). In an exceptional system like NN Ser, where the WD is known to harbour *t*_cool_∼1 Myr [136], a second-generation planet would be the youngest known exoplanet, and help constrain the formation process.

Second-generation formation provides intriguing possibilities for planetary system evolution. Perets [279] highlighted some important concepts on this topic: (i) post-MS evolution opens up dynamically ‘previously forbidden’ regions of SB formation, (ii) pre-existing first-generation planets may interact with newly-formed second-generation planets, (iii) when each of two binary components undergoes post-MS evolution, there exists the prospects for third-generation formation, which is distinct from second-generation formation and (iv) second- and third-generation discs may be metal-enriched in order to mirror the expected composition in stellar ejecta. These systems may conspicuously stand out in very metal-poor environments like globular clusters (on their outskirts, from dynamical stability) or simply around metal-poor stars.

### 8.1 Post-common envelope formation around white dwarfs

The amount of disc mass available for planet formation is a fraction of the amount of mass that remains bound after the CE. The process for planet formation then proceeds along similar lines to the MS case, except here the disc may be influenced by photoheating, photoionization and radiation pressure (see sections 4.1–4.3 of [77]) in different manners. The metal content may also be different.

Bear & Soker [284] used angular momentum arguments to suggest that planets orbiting post-CE binaries are more likely to be first generation than second generation. Their table 1 displays the minimum angular momentum of the putative post-CE binary planetary systems, as well as angular momentum efficiency factors for each system. The crucial unknowns are the efficiency factors, which determine (i) how much initial angular momentum is deposited into planets and (ii) how much of the envelope ends up in the disc. For NN Ser in particular, Schleicher & Dreizler [77] provided in their table 1 some model parameters for the formation by gravitational instability. Völschow *et al.* [220] observed that as second-generation planets form in systems like NN Ser, the formation disc may still be expanding.

In WD–AGB binary systems, the formation of a second-generation disc may be quantified in a similar way as in an MS–AGB binary system. Through angular momentum arguments, Perets & Kenyon [285] provided a criterion for second-generation disc formation around the primary star from the wind of a binary companion (their eqn 1):

8.1 $$1≲\left(\frac{M_{⋆}+M_{b}}{2.5\;M_{⊙}}\right){\left(\frac{M_{⋆}}{M_{⊙}}\right)}^{3/2}{\left(\frac{R_{⋆}}{R_{⊙}}\right)}^{-1/2}{\left(\frac{a}{100\;\mathrm{AU}}\right)}^{-3/2}{\left(\frac{|v_{b}^{\left(\mathrm{wind}\right)}-v|}{10\;\mathrm{km}\;s^{-1}}\right)}^{-4}.$$

### 8.2 Post-supernova formation around neutron stars

*General considerations* Miller & Hamilton [73] and Hansen [286], and references therein, summarized multiple possible scenarios for the formation of a circumpulsar disc: (i) a disc forms from binary mass transfer, where the donor is disrupted or evaporated, (ii) a disc forms from donor ejecta, then the donor is evaporated, (iii) a disc forms from donor ejecta, and then the donor goes SN and gravitationally unbinds the disc-bearing pulsar, (iv) a disc forms from ejecta from an SN-induced direct collision (where the SN kick was in the direction of the other star) and (v) a disc forms from ejecta from a WD–WD direct collision, which could form a pulsar and an accompanying disc. Second-generation pulsar planets do not necessarily need a disc if the planets either formed directly from fallback matter or the companion was evaporated just the right amount to achieve a planet mass.

Miller & Hamilton [73], who do not favour first-generation scenarios for the known pulsar planets, also placed restrictions on post-SN second-generation formation through stellar evolution arguments: if a pulsar was transformed into a millisecond pulsar by accreting matter, then they argue that the luminosity from the accretion would evaporate the planet. If this accretion process occurred and left a remnant second-generation disc, then the authors argued that stellar luminosity would disperse the disc too quickly too allow for formation.

In a wide-ranging dynamical study of second-generation formation and evolution, the cleverly titled paper by Cordes and Shannon [194] covered collisional migration, the Yarkovsky effect, magnetospheric currents, electromagnetic capture, heating and evaporation of SBs, and disc lifetimes. Their main finding is that second-generation asteroids, but not planets, could easily form and create observable signatures in systems like B1931+24. This scenario relies on (i) the SN fallback disc to be metal-rich and ‘sufficiently compact and low in mass (10^−6^ *M*_⊙_) to prevent’ massive planet formation, (ii) the mass inflow into the light cylinder to be large enough to influence magnetospheric current flows on relevant timescales and (iii) the disc survival time must be long enough (greater than or equal to 10 Myr) to alter the electrodynamics of old pulsars. A couple of interesting ancillary findings include the fact that (i) rocks with high tensile strengths can form metal-rich gas inside the tidal disruption radius. These second-generation rocks are not like the strengthless rubble piles seen in the Solar system because they are metal-rich and fractionated. Further, (ii) the radiative effects on second-generation asteroids near the pulsar’s light cone are comparable to currently observed Solar system Yarkovsky drift rates (approx. 10^−3^ AU Myr^−1^).

*PSR B1257+12.* Before delving into the possibilities for second-generation formation in PSR B1257+12, one may ask if mass loss alone hypothetically (ignoring engulfment) could induce ejection of the SBs. To help answer this question, Veras *et al.* [208] considered the likely value of *Ψ* (see equation (4.10)) for the PSR B1257+12 planets during their SN mass loss. Even though the mass is lost from SN quickly, the small MS semi-major axis values (<0.1 AU) of all three planets was too small to place them in the strongly non-adiabatic regime. Actually, *Ψ*∼0.1–1.0, and therefore the planet eccentricities were not pumped sufficiently high to cause ejection.

For a second-generation origin, Currie & Hansen [287] performed a dedicated modelling suite for the PSR B1257+12 disc. They considered both fully viscous discs and layered accretion discs. They found that discs typically contain material out to 1–2 AU, and are sufficiently massive to produce the PSR B1257+12 planets. Because the gas in these discs dissipates in under 0.1 Myr, gas giants are unlikely to form. The follow-up study of Hansen *et al.* [288] considered how planetesimals from second-generation discs would assemble into a planetary architecture. They found that because the density of solid material must be sufficiently high for planet formation to commence, one can impose restrictions on the speed of dust sedimentation during disc cooling. Their study provides further support of the post-SN fallback scenario (as opposed to a binary companion disruption scenario) for the origin of the PSR B1257+12 planets.

### 8.3 Formation from tidal disruption of companions

*Pulsar stellar binaries.* The PSR B1257+12 planets may have been formed through one of a few different second-generation channels. Currie & Hansen [287] provided a comparison. Instead of forming out of stellar fallback, these planets might have formed out of shorn-off binary star matter. As opposed to fallback discs, with angular momentum values as low as 10^42^ *J* s^−1^, disruption discs could harbour angular momentum values three orders of magnitude higher. Because fallback discs tend to be more compact than disruption discs, a fallback disc is more likely to reproduce the planets in the PSR B1257+12 system. Similar to the SN fallback disc, in an SN disc formed from tidal disruption gas dissipates too quickly to form giant planets.

In a similar vein, Shannon *et al.* [186] suggested that if the putative asteroid disc around PSR B1937+21 is a second-generation disc, then it might have formed from the ablated material of a disrupted stellar companion. They proposed that the resulting disc was too tenuous to form planets, which is why none are detected. Instead, the planet formation process would stop after a brief period of runaway growth, when asteroids were formed. Formation could occur throughout a radial distance of several AU, because the disc would not be truncated by the presence of planets. This second-generation disc itself might feature many SBs on non-crossing orbits and whose evolution is described in their sections 4.1–4.2. They suggested that inwardly-migrating SBs will never reach the star’s magnetosphere before being evaporated.

*White dwarf stellar binaries.* Livio *et al.* [57] predicted the existence of second-generation debris discs, and possibly planets, around massive WDs as a result of a previous WD–WD merger. They envisaged the merger of two 0.5 *M*_⊙_ WDs forming a single approximately 1.0 *M*_⊙_ WD with a residual disc. Based on angular momentum arguments and a power-law surface density disc profile, they found

8.2 $$M_{d}=0.81\;M_{⋆}{\left(\frac{R_{⋆}}{r_{d}^{(\max)}}\right)}^{1/2},$$

where the inner (minimum) disc radius is presumed to coincide with the WD surface. They do not model planetesimal formation, but assume that it commences when $r_{d}^{(\max)}$ exceeds the dust grain condensation line, which they estimated to be

8.3 $$r_{d}^{\left(\mathrm{dgline}\right)}=0.02\;\mathrm{AU}\left(\frac{R_{⋆}}{6\times 10^{6}\;m}\right){\left(\frac{T_{⋆}}{50000\;K}\right)}^{2}{\left(\frac{T_{d}^{\left(\mathrm{dgline}\right)}}{1600\;K}\right)}^{-2},$$

where $T_{d}^{\left(\mathrm{dgline}\right)}$ is the disc temperature at which dust grains condense.

*Large substellar bodies.* Bear & Soker [289] indicated that while the envelope of a massive planet that is being destroyed may form a gaseous disc, the rocky fragments could represent small planets that then migrate within the disc; Bear & Soker [290] made a similar suggestion. Alternatively, Guillochon *et al.* [291] and Liu *et al.* [292] intimated that a first-generation planet may be transformed into a second-generation planet by being tidally disrupted. In other words, after each close disruptive pericentre passage of a gas giant planet and an MS (or, nearly equivalently, WD) star, the planet fundamentally changes character. For coreless planets [291], when free-falling ejecta accretes onto the post-encounter planetary remnant, then its outer layers spin faster and contain more mass, changing the atmospheric structure. Although subsequent passages may destroy the planet, other bodies further away could perturb the disrupting planet into a stable orbit. Cored giant planets can transform into super-Earths or Neptune-like planets through this process [292].

## 9. White dwarf disc formation from first-generation substellar bodies

The compact debris discs which have been observed to orbit isolated (single) WDs at distances of ($r_{d}≲1.2R_{⊙}$) cannot have formed during the MS or GB phases. These discs also cannot have formed from fallback stellar material because of their age and composition. The canonical explanation is that they formed from the disruption of first-generation SBs [114,224,293], and in particular, asteroids. See Farihi [18] for an observationally based review of these discs. The orbits of the progenitors are assumed to be highly eccentric (e≳0.998) because their semi-major axes must be at the very least a few AU. Otherwise, these SBs would have been engulfed, vapourized or spun-up to fission during the GB phases.

*Tidal disruption radius.* Consequently, and emphasized by the WD 1145+017 system (see §3.2.1), the process of tidal disruption plays a crucial role in the interpretation of observations of post-MS systems. A primary consideration is then identifying the tidal disruption distance, *r*_*t*_, which defines a disruption sphere, or Roche sphere, around the WD. This value depends on many factors, including the SB’s shape, spin state, composition and orbital state, as well as if disruption is defined as cracking, deforming or dissociating entirely.

A formulation which includes the internal strength of the SB is (rearranged from eqn 8 of [290]):

9.1 $$r_{t}={\left[\frac{2GM_{⋆}\rho_{\mathrm{SB}}R_{\mathrm{SB}}^{2}}{\gamma_{\mathrm{SB}}+\frac{4}{3}\pi G\rho_{\mathrm{SB}}^{2}R_{\mathrm{SB}}^{2}}\right]}^{1/3},$$

where *γ*_SB_ is the critical tensile strength of the SB that is disrupting. For a strengthless SB, which is representative of many ‘rubble pile’ Solar system asteroids, this equation reduces to *r*_*t*_≈1.26*R*_★_(*ρ*_★_/*ρ*_SB_)^1/3^. However, because of the multiple dependencies mentioned above, Bear & Soker [294] re-expressed this relation in their eqn 1 with the following range of coefficients: *r*_*t*_≈[1.3−2.9]*R*_★_(*ρ*_★_/*ρ*_SB_)^1/3^. Alternatively, for bodies with high tensile strengths, equation (9.1) reduces to eqn 3 of [194], modulo a factor of 1.26. Another alternative is eqn 5 of [67], where the internal strength dominates and the SB is characterized by solid-state forces and a corresponding internal sound speed. Then, $r_{t}\propto G^{1/3}M_{⋆}^{1/3}R_{\mathrm{SB}}^{2/3}v_{\mathrm{sound}}^{-2/3}$. Typical SB speeds within the disruption radius can reach 10^3^ km/s (eqn 7 of [295]).

*Tidal disruption simulations.* The above relations allow one to consider modelling the process of tidal disruption with analytics and simulations. Debes *et al.* [59] performed simulations of the breakup of strengthless 5000-particle rubble-pile asteroids by a 0.5 *M*_⊙_ WD with initial orbital semi-major axes of 4.77 AU and pericentres of 60, 65, 70, 75 and 80 *R*_★_ (so *e*>0.999). They modelled a single pericentre passage and analysed the cumulative size distribution of the resulting fragments in the stream (see their fig. 6). They found that for smaller pericentres, more large fragments are generated, and that the stream largely followed the initial orbit.

Veras *et al.* [295] later also modelled the tidal disruption of highly eccentric strengthless 5000-particle rubble-pile asteroids, but did so for over 100 orbits. They established that the time an SB spends within the disruption sphere is largely independent of *a* (their fig. 1), enabling them to perform multi-orbit simulations by scaling down the orbit. They found that the SB disruption eventually creates a highly collisionless eccentric ring in the same shape as the original orbit, in the absence of other forces. Other forces might cause the ring to become collisional, as suggested by Bear & Soker [294].

*Ring formation analytics.* Veras *et al.* [295] also derived a closed-form expression for the filling time, *t*_fill_, of this ring (their eqn 25) supposing that the breakup occurs instantaneously at some *r*_u_<*r*_t_:

9.2 $$\begin{array}{rl} \frac{t_{\mathrm{fill}}}{P_{\mathrm{SB}}} & =r_{u}^{3/2}{\left[{\left\{\frac{r_{u}^{2}+2aR_{\mathrm{SB}}-r_{u}R_{\mathrm{SB}}}{r_{u}-R_{\mathrm{SB}}}\right\}}^{3/2}-{\left\{\frac{r_{u}^{2}-2a\min(r_{\mathrm{crit}}-r_{u},R_{\mathrm{SB}})+r_{u}\min(r_{\mathrm{crit}}-r_{u},R_{\mathrm{SB}})}{r_{u}+\min(r_{\mathrm{crit}}-r_{u},R_{\mathrm{SB}})}\right\}}^{3/2}\right]}^{-1}, \end{array}$$

where *P*_SB_ is the orbital period of the SB and where *r*_crit_ is the location at which the SB’s orbit would become parabolic

9.3 $$r_{\mathrm{crit}}=\frac{2ar_{u}}{\left(1+\frac{M_{\mathrm{SB}}}{M_{⋆}}\right)(2a-r_{u})}\approx \frac{2ar_{u}}{2a-r_{u}}.$$

Equation (9.2) does not make any assumptions about orbital eccentricity.

However, as indicated by figure 2, considering gravity alone in a regime where $r≲10^{-3}\;\mathrm{AU}$ is inadequate. Veras *et al.* [70] modelled the long-term effect of WD radiation on these collisionless rings. They found that WD radiation alone compresses and circularizes orbits of SBs with *R*_SB_≈10^−5^–10^−1^ m to within the WD disruption radius. See their eqns 20–22 for closed-form approximations of the evolution of *a* and *e*. Outside of this SB size range, radiative effects such as the Yarkovsky effect and radiation scattering may dictate the motion, and have not yet been explored. For this size range, they derive in their eqn 23 a closed-form expression for the shrinking time *t*_shr_, which is better expressed as

**Figure 14.**
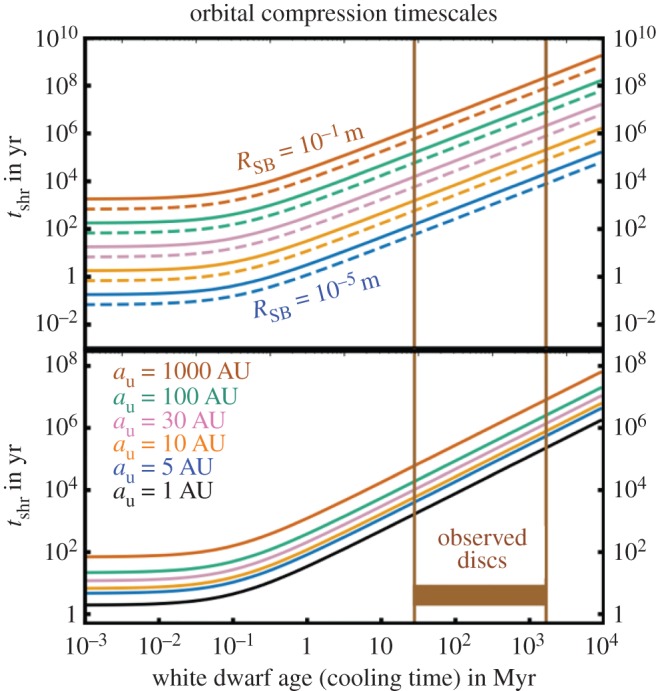
Cosmetically enhanced version of fig. 2 of [70]. How particles of given radii and semi-major axis shrink due to the effects of radiation alone, as a function of WD cooling time.

9.4 $$\begin{array}{rl} {\left(1+\frac{10(t_{\mathrm{shr}}+t_{u})}{\mathrm{Myr}}\right)}^{-1/5} & =\left(1+\frac{10t_{u}}{\mathrm{Myr}}\right)\\ & \;-2.39\left(\frac{\rho_{\mathrm{SB}}R_{\mathrm{SB}}c^{2}q_{u}^{3/2}}{L_{⋆}(t_{\mathrm{cool}}=0)[Q_{\mathrm{abs}}+Q_{\mathrm{ref}}]\mathrm{Myr}}\right)\left(\sqrt{a_{u}}-\sqrt{2q_{u}+r_{t}}\right), \end{array}$$

where *c* is the speed of light, where *Q*_abs_ and *Q*_ref_ are the SB’s absorption efficiency and reflecting efficiency (albedo), *t*_u_ is the cooling age at which disruption occurred, and *a*_u_ and *q*_u_ are the orbital semi-major axis and pericentre at disruption. The value of *t*_shr_ may change when additional effects, such as sublimation, are taken into account. Figure 14 illustrates the shrinking time as a function of cooling age. Because the shrinking timescale is dependent on *R*_SB_, differently-sized pebbles will shrink at different rates, fanning out the original ring. Further, as the SBs approach the WD, the effect of general relativity becomes more important (see §12.1). Throughout the contraction, collisions may occur, which could affect subsequent shrinking times.

Stone *et al.* [67] instead adopted a constant WD luminosity and in the high-eccentricity limit obtained a characteristic shrinking timescale (their eqn D6) that is proportional to $a_{u}^{1/2}q_{u}^{3/2}/L_{⋆}$. The quality of this approximation increases as the WD gets older, when *L*_★_ experiences less-drastic changes.

*Other disruption processes.* How the tidal disruption of an asteroid encountering a WD compares with much better-studied tidal disruption situations in the literature is not yet clear. As observed by Bear & Soker [294], stars that pass close to black holes typically result in about half of their mass being ejected, with the remaining mass residing on highly eccentric orbits. A perhaps more relevant case are planets disrupting around MS stars [291,292]. In a rough qualitative sense, this situation should be similar for planet destruction around WDs.

## 10. White dwarf disc evolution

Our understanding of how WD discs evolve crucially impacts observational targeting strategies and touches upon both how these discs formed (§9) and how they accrete onto the WD (§11). More directly and perhaps just as importantly, we cannot yet explain the variability in the dusty components of discs such as that around WD J0959-0200 [115] and the axisymmetric structures in the gaseous components of discs such as that around SDSS J1228+1040 [122]. Xu & Jura [115] speculated that the drop in the flux of the WD J0959-0200 disc could be attributed to a recent SB impact or instability within the disc. More detailed models may help distinguish these possibilities.

*Solid body evolution during impact.* Jura [114] emphasized the potential connection of WD discs with planetary rings in the Solar system. In line with this analogy, Jura [224] discussed disc evolution following additional impacts (which can generate additional dust). He posed that the disc evolution would be determined by size: small entrants would be destroyed by sputtering, whereas large bodies more massive than the disc would imprint their trajectories on the disc.

Assume that an asteroid tidally breaks up and produces a stream of identical grains with density *ρ*_SB_ and orbital inclination *i* measured with respect to the existing WD debris disc plane. Assume also that the orbit of the grains passes through the disc twice per orbit, the orbit does not change, and that the grain material is equivalent to the disc material. After many orbits, the minimum surviving grain size is (from eqns 15–17 of [224])

10.1 $$R_{\mathrm{SB}}^{\left(\min\right)}=yt\frac{\Sigma_{d}}{4\pi \rho_{\mathrm{SB}}\sin i}\sqrt{\frac{GM_{⋆}}{a_{\mathrm{SB}}^{3}}},$$

where *y* is the (dimensionless) sputtering yield, and *Σ*_d_ is the debris disc density. The detectable infrared excess which results from the dust produced in the encounters diminishes as the grain radius decreases. Jura [224] found that the excess from the encounter dissipates on a much shorter timescale than the characteristic disc lifetime.

*Gas generation.* Now consider the incidence of gas in these discs. When and where gas is produced represents a crucial aspect of the disc evolution. In principle, all dusty discs should produce gas through sublimation as the inner rim falls towards the WD. However, this gas has been detected in only 7 of the nearly 40 dusty discs so far (see §3.1), and in those 7 discs, the gas has a wide radial extent, overlapping with the dust. This overlap highlights a curious puzzle.

An alternative gas generation mechanism is collisions of solid particles. Jura [224] suggested that mutual velocities between the grains may be as high as 10^3^ km s^−1^ (perhaps in line with eqn 7 of [295]), which could generate copious amounts of gas. However, Metzger *et al.* [296] suggested (in their section 6.4) that this process produces a negligible amount of gas because the initially high relative velocities are quickly damped through inelastic collisions. They pose that more gas would be generated and persist for tens or hundreds of years immediately after a disruption event, as with an incoming SB interacting with an already evolving disc.

Returning to sublimation, the distance at which the sublimation takes place, *r*_sub_ (often refereed to as the sublimation radius), is usually expressed as (eqn 1 of [250])

10.2 $$r_{\mathrm{sub}}=\frac{R_{⋆}}{2}{\left(\frac{T_{⋆}}{T_{\mathrm{SB}}^{\left(\mathrm{sub}\right)}}\right)}^{2}\approx 22R_{⋆}{\left(\frac{T_{⋆}}{10^{4}\;K}\right)}^{2}{\left(\frac{T_{\mathrm{SB}}^{\left(\mathrm{sub}\right)}}{1500\;K}\right)}^{-2},$$

demonstrating that particles typically sublimate at a distance of a few tens of WD radii. However, Rafikov & Garmilla [113] argued that one fundamental difference between WD debris discs and protoplanetary discs is that because the former is hydrogen poor, values of *T*^(sub)^_SB_ should be several hundred K higher. Also, equation (10.2) does not include physics such as conduction or reflectance (eqn 1 of [297] includes a factor incorporating particle and stellar emissivity). Note tangentially that a direct comparison with the other sublimation distance expression in this paper (equation (6.12)) shows significant differences: In equation (10.2), the only explicit dependence on SB properties is through its sublimation temperature.

*Disc temperature.* That equation does not actually require the presence of a disc. If a disc does exist, then the temperature is not necessarily uniform throughout: an isothermal disc would in fact not produce any detectable flux, according to equation (3.1).

The temperature at a given location in the disc is dependent on (i) the optical thickness at that location, (ii) the amount of shielding, (iii) heating by the WD, (iv) heating by gas, (v) cooling by sublimating atoms and (vi) cooling by thermal radiation from particle surfaces. Rafikov & Garmilla [113] began to address these issues in their appendix A, and related disc temperatures in optically thin and thick parts of the disc through (their eqns 1–2)

10.3 $$T_{d}^{\left(\mathrm{thin}\right)}(r_{d})\approx T_{⋆}{\left(\frac{1}{2}\right)}^{1/2}{\left(\frac{R_{⋆}}{r_{d}}\right)}^{1/2}$$

and

10.4 $$T_{d}^{\left(\mathrm{thick}\right)}(r_{d})\approx T_{⋆}{\left(\frac{2}{3\pi }\right)}^{1/4}{\left(\frac{R_{⋆}}{r_{d}}\right)}^{3/4}.$$

*Disc viscosity.* One key aspect of WD discs is the viscous timescale of their gaseous components

10.5 $$t_{d,\;\mathrm{gas}\;\mathrm{only}}^{\left(\mathrm{visc}\right)}∼\frac{r_{d}^{2}}{\upsilon_{d}},$$

where *υ*_d_ is the viscosity of the gaseous component, and is assumed to be constant, and *r*_d_ represents a location in the disc. Unfortunately, the viscous timescale parameter is unconstrained. In fact, the *t*^(visc)^_d_ values reported by Bear & Stoker [294] and Metzger *et al.* [296] differ by six orders of magnitude (0.75 day to 2000 years). Bear & Soker [294] attributes this discrepancy to different assumptions about when the disc was formed, and the resulting different values of sound speed, viscosity parameter and scale height.

Gas will exist inside of any dusty disc where $r_{d}^{\left(\mathrm{out}\right)}>r_{\mathrm{sub}}>r_{d}^{\left(\mathrm{in}\right)}$, with *r*^(in)^_d_ and *r*^(out)^_d_ representing the boundaries of the disc. Gas will also exist outside of the disc, at *r*<*r*^(in)^_d_, as the gas accretes onto the WD. Gas is thought to accrete onto the WD in order to maintain angular momentum conservation with the gas’s outward viscous spreading. For the seven discs with both dusty and gaseous components, the latter is no more massive than the former. This statement arises from estimates of the dust mass [114,117,119,224] and of the gas mass [298]. For a disc like SDSS J1228+1040, the dust mass may be about 10^19^–10^24^g whereas the gas mass is about 10^19^g [122,298]. The presence of dust and gas, even if not in the same proportion, showcase the necessity of considering coupled evolution models. As observed by Metzger *et al.* [296], just how gas and dust could exist in the same location in different phases is an outstanding question.

Nevertheless, considering gas only can provide initial helpful insight. By assuming an isothermal gas-only disc is turbulent with a viscosity equal to $\alpha v_{\mathrm{sound}}^{2}r_{d}^{3/2}/\sqrt{GM_{⋆}}$ (according to the typical *α* parametrization with the sound speed *v*_sound_), this disc’s viscous timescale can be expressed as (eqn 2 of [296])

10.6 $$t_{d,\;\mathrm{gas}\;\mathrm{only}}^{\left(\mathrm{visc}\right)}\approx 2\;\mathrm{yr}\times \alpha^{-1}{\left(\frac{T_{d}^{\left(\mathrm{gas}\right)}}{5000\;K}\right)}^{-1}\left(\frac{\mu }{28M_{H}}\right){\left(\frac{M_{⋆}}{0.6\;M_{⊙}}\right)}^{1/2}{\left(\frac{r_{\mathrm{sub}}}{0.2R_{⊙}}\right)}^{1/2},$$

where *M*_H_ is the mass of hydrogen and *μ* is mean molecular weight (equal to 28*M*_H_ for silicon). The largest source of uncertainty in equation (10.6) is the unknown *α*. For an alternative formulation of the viscosity, as expressed by a power law and with sources and sinks of mass, see appendix B of [296]. Note also that if equation (10.6) is applied to a disc containing dust (as all WD discs do), then $T_{d}^{\left(\mathrm{gas}\right)}$ would not be equal to *T*^(dust)^_d_ due to their different heating and cooling sources and sinks.

As a first approximation, equations like equation (10.6) help provide scalings for observed discs like SDSS J1228+1040 [122], and can help assess the steady-state accretion assumption (equation (11.2)) for individual WDs. However, one should keep in mind that angular momentum transfer in a gaseous disc is more efficient than in a dusty counterpart, leading to a shorter lifetime.

*Coupled dust-gas evolution.* The coupling between dust and gas in WD debris discs has been studied [113,250,296,297,299]. Rafikov [297] suggested that the coupling leads to ‘runaway’ accretion due to positive feedback from newly formed gas. Forces between concomitant gas and dust rotating at different rates enhances solid body angular momentum loss, causing this runaway. Rafikov [250] supported these results, in part, by evaluating and discounting the prospect for the Yarkovsky force (see §6.1.1 of this paper) opposing the inward Poynting-Robertson drag, even despite the unknown size distribution in the WD debris disc. Bochkarev & Rafikov [299] performed a global disc analysis. They found a ‘universal appearance’ of a sharp outer edge in optically thick discs. But this edge moves inward, and so can easily turn into a narrow ring. Rafikov & Garmilla [113] explored the effect of shielding and vapour pressure.

Metzger *et al.* [296] performed the most recent global time-dependent simulations of WD discs with dust and gas, and included (i) aerodynamic coupling in two limiting cases of optical depth (their sections i and ii) and (ii) mass exchange (their section 3). In the optically thin case, where solid particles individually interact with the gas, they provide expressions for the particle Reynolds number (their eqn 13)

10.7 Re=2RSBvϕ(rel)υm

and frictional drag force (their eqn 14)

10.8 $$F_{\mathrm{drag}}^{\left(\mathrm{fric}\right)}=\left\{ \begin{array}{ll} \frac{4\pi }{3}R_{\mathrm{SB}}^{2}\rho_{\mathrm{gas}}v_{\mathrm{sound}}v_{\phi }^{\left(\mathrm{rel}\right)}, & R_{\mathrm{SB}}≲\zeta \\ 6\pi R_{\mathrm{SB}}\rho_{\mathrm{gas}}\upsilon_{m}v_{\phi }^{\left(\mathrm{rel}\right)}, & R_{\mathrm{SB}}≳\zeta . \end{array}\right.$$

In equations (10.7) and (10.8), *v*_sound_ is the sound speed, the value *υ*_m_ is the molecular shear viscosity (not turbulent viscosity), *ρ*_gas_ is the midplane gas density and $v_{\phi }^{\left(\mathrm{rel}\right)}$ is the azimuthal speed difference between the particles and gas. Comparing these equations with equations (4.21) and (4.23) illustrates that, besides numerical factors of order unity, the difference comes here from the inclusion of molecular shear viscosity. Also, as in equation (4.21), the upper and lower pieces of equation (10.8) correspond to the Epstein and Stokes regimes, respectively.

**Figure 15.**
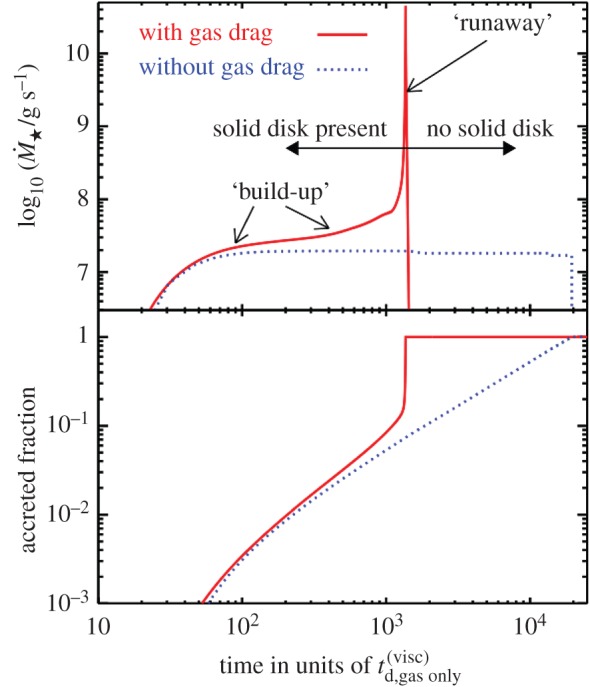
Cosmetically enhanced version of fig. 7 of [296]. How a coupled gas and dust WD debris disc becomes a ‘runaway’ disc, which features a burst of accretion at the inner rim. The difference in the dotted blue and solid red lines illustrates the importance of including coupling in WD debris disc models.

Metzger *et al.* [296] ultimately provide support for the runaway theory, characterizing the process as a ‘buildup’ and then ‘runaway’ phase as an optically thick disturbance migrates inwards and then provides positive feedback at the inner rim (figure 15). Their finding that eccentricity in gas motions as small as 10^−4^ are highly efficient at driving runaway accretion enables them to make an important prediction: the asymmetric line profiles in observed gaseous disc components indicate non-axisymmetric surface brightness rather than eccentric gas motions.

*Secular evolution.* Observations by Manser *et al.* indicate that at least one disc (SDSS J1228+1040; see figure 7 of this paper) has secular modes which act on timescales (tens of years) that are five orders of magnitude greater than orbital timescales (hours). The authors discuss several possible origins for the disc evolution: (i) an external perturber, (ii) the youth of the disc and (iii) self-generated perturbations. An almost certainly important consideration in all three scenarios is precession due to general relativity: the disc will precess over one complete orbit due to general relativity (see equation (12.1)) in about 27 years, which matches with the secular period (the precession period ranges between 1.5 and 134 years for small eccentricities and *a*=0.2*R*_⊙_−1.2*R*_⊙_). The authors excluded the first scenario based on *N*-body simulations of an external perturbing body, which changes the disc too quickly (only during pericentre passages). Either or both of the other scenarios might work, but require further modelling with radiative and collisional effects. In the second scenario, the disc just formed from a tidal disruption and is in the process of settling and circularizing. Modelling the third scenario is difficult because the mass ratio of gas to dust in the disc is poorly constrained, ranging from 10^−5^ to unity.

## 11. Accretion onto white dwarfs

WDs with photospheric signatures of heavy metals are considered to be polluted because they accrete these metals from an external origin. Understanding the process of pollution is then vital to achieve links with observables.

*Settling phases.* Assume that a particular chemical element is accreting onto the WD at a constant rate ${\dot{M}}_{\mathrm{ele}}$ with a constant characteristic diffusion, or settling timescale *t*^(elm)^_set_ through the bottom of the convection zone. The mass of the convection zone is *M*_conv_, which is calculated from a stellar model. These assumptions allow one to obtain the following explicit formula (eqn 5 of [300]) for the time evolution of the mass fraction of that chemical element, *X*_elm_(*t*), assuming that the element starts accreting at *t*=*t*_start_:

11.1 $$X_{\mathrm{elm}}(t)=X_{\mathrm{elm}}(t_{\mathrm{start}})\cdot \exp\left(-\frac{t-t_{\mathrm{start}}}{t_{\mathrm{set}}^{(\mathrm{elm})}}\right)+t_{\mathrm{set}}^{(\mathrm{elm})}\frac{{\dot{M}}_{\mathrm{ele}}}{M_{\mathrm{conv}}}\left[1-\exp\left(-\frac{t-t_{\mathrm{start}}}{t_{\mathrm{set}}^{(\mathrm{elm})}}\right)\right].$$

Equation (11.1) describes three important regimes, as explained by Koester [48,300]: (i) onset: $t-t_{\mathrm{start}}\ll t_{\mathrm{set}}^{(\mathrm{elm})}$, (ii) steady state: $t-t_{\mathrm{start}}\gg t_{\mathrm{set}}^{(\mathrm{elm})}$ and (iii) trailing: *t*−*t*_start_≫*t*^(elm)^_set_ plus ${\dot{M}}_{\mathrm{ele}}\to 0$. Distinguishing between these modes for observations of accretion in helium-rich WDs (‘DB WDs’) is unfortunately not yet possible, because of the long sinking times (up to Myr). This degeneracy does not arise for hydrogen-rich WDs (‘DA WDs’) which are older than about 300 Myr (see fig. 1 of [98]) because their convection zones are too small and the element diffusion times are too fast (typically, days to weeks). The typically-used steady state assumption importantly allows one to obtain abundance ratios between chemical element pairs (labelled #1 and #2 below) because in this regime, both exponential terms in equation (11.1) vanish while accretion is ongoing. Consequently,

11.2 $$\frac{X_{\mathrm{elm}\#1}}{X_{\mathrm{elm}\#2}}=\frac{t_{\mathrm{set}}^{(\mathrm{elm}\#1)}{\dot{M}}_{\mathrm{elm}\#1}}{t_{\mathrm{set}}^{(\mathrm{elm}\#2)}{\dot{M}}_{\mathrm{elm}\#2}},$$

where the quantities on the right-hand-side are known. Therefore, abundance ratios of different species are quantities which are readily reported in WD pollution literature.

Note importantly that equation (11.1) does not necessarily require the existence of a debris disc, rather just a constant stream of pollution from an external source (perhaps from a stream of tidally-disrupted matter). Jura *et al.* [301] (and also [92]) considered the case when a debris disc exists, was formed by a single progenitor, and decays in an exponential manner, with a characteristic time of *t*_d_. In this case, they derived an expression for the total mass of a particular element that existed in the progenitor, denoted by *M*^(elm)^_SB_. By also assuming $t_{d}>t_{\mathrm{set}}^{(\mathrm{elm})}$ and that the currently-measured/modelled convection zone mass of that element only is *M*^(elm)^_conv_, they derive (from eqn 8 of [301] or from eqns 7–8 of [92])

11.3 $$M_{\mathrm{SB}}^{\left(\mathrm{elm}\right)}=M_{\mathrm{conv}}^{\left(\mathrm{elm}\right)}\left(\frac{t_{d}}{t_{\mathrm{set}}^{(\mathrm{elm})}}-1\right){\left\{\exp\left(-\frac{t-t_{\mathrm{start}}}{t_{d}}\right)-\exp\left(-\frac{t-t_{\mathrm{start}}}{t_{\mathrm{set}}^{(\mathrm{elm})}}\right)\right\}}^{-1}.$$

*Thermohaline convection.* The above analysis is for gravitational settling alone. Deal *et al.* [302] warned that neglecting thermohaline (or fingering) convection could induce steady states before they actually occur in DAZ (hydrogen and metal-enriched) WDs. Thermohaline convection occurs when upper layers of a WD atmosphere are heavier than lower layers, which could induce localized mixing. Wyatt *et al.* [98] helpfully used the analogy of salt fingers in Earth’s oceans. However, Xu *et al.* [88] argued against the conclusions of Deal *et al.* [302], claiming that (i) their thermohaline prescription asymptotically approaches infinity at the bottom of the convection zone, (ii) one of their models does not contain any atmospheric convection and therefore cannot be representative of canonical polluted WDs like G 29-38 and (iii) they do not consider the fuzziness of the convection zone boundary, and the consequences for mixing. Koester [303] presented more detailed arguments.

*Convective overshooting.* Because convection zones in WD atmospheres are usually defined as the boundaries where instabilities to convection occur (the Schwarzschild criterion), these zones do not take into account real convection cells, or bubbles, that traverse or are said to ‘overshoot’ the boundaries. Freytag *et al.* [304] pioneered the modelling of this process in WDs. Recent progress (e.g. section 5.2 of [305]) suggests that overshooting may represent an important consideration for polluted WDs, although further modelling is necessary.

*Accretor size distributions.* Observationally derived accretion rates can help us distinguish the size distribution of the accreting SBs. The metal content of the SB combined with the type of WD (DA or non-DA) determines the sinking time through the convection zone. If SBs (from a disc or not) of a certain size rain onto a particular WD frequently enough, then their accretion signature is always present in the atmosphere. This accretion is said to be ‘continuous’ accretion. Alternatively, if larger SBs infrequently penetrate the atmosphere, then they ‘stochastically’ accrete onto the WD.

Wyatt *et al.* [98] helped distinguish size distributions that would produce continuous versus stochastic accretion. They found that the critical value of the SB radius which divides these two regimes is between about 1.7 km and 170 km. The critical radius is larger for non-DA WDs because of their larger convective zones. They also considered, section-by-section, the quality of observational matches from accretion distributions based on (i) mono-mass, mono-rate accretion (their sections 4.1 and i) and (ii) mono-mass, multi-rate accretion (their sections 4.2 and ii). In all these cases, they effectively debunk the idea that pollution arises from any type of mono-mass distribution.

A more complex multi-mass distribution is required, and might have to incorporate the potential presence of a WD disc. The accretion rate might depend on the disc lifetime. Regardless, in the course of their study, Wyatt *et al.* [98] used an analytical model that might be helpful for future investigations. Their stochastic mono-mass accretion model is equivalent to that of shot noise in electrical systems (see their appendix A), providing for an analytical foundation (particularly their eqns 9 and 13) which is an excellent match to Monte Carlo simulations (see their fig. 4).

*Impactor physics.* Stochastic accretion will lead to direct impacts onto the WD by SBs larger than pebbles. Boulders, asteroids, comets and planets may directly hit the WD, bypassing any disc phase. The physics of impact in the context of NSs and WDs has been studied in the 1980s [156,306], and more recently for the Sun [307,308], but requires more current WD-specific attention. Brown *et al.* [307] distinguished three possible outcomes, which are not mutually exclusive: explosion, ablation and sublimation. Alcock *et al.* [156] instead characterized the impact process through explosion, spreading and sedimentation phases.

*Disc accretion.* The alternative to stochastic accretion is continuous accretion—from a disc (§§9–10). The observed discs, however, do not extend to the WD surface (or photosphere). The inner rims of dusty disc components reside tens of WD radii outside of the WD surface. The gap between the WD and the inner rim is then assumed to be filled with sublimated metallic gas originating from the inner rim. This gas is accreted onto the WD though viscous torques [250]. Hence, in effect, the mass flux through the inner rim acts as a proxy for the actual accretion rate onto the WD surface. Estimating this mass flux is particularly important because the accretion rate onto WDs is a quantity derived from observations (by combining sinking times with convection zone metal content).

Computations of this mass flux necessarily rely on modelling the coupling between dust and gas, which is not trivial, as already explained in §10 and illustrated explicitly in eqn 4 of [297]. One of the dominant forces widely thought to influence this mass flux is Poynting–Robertson drag, for which, when acting in isolation, several estimates have been given (eqn A8 of [309], eqn 9 of [250], eqn 5 of [297], eqn 43 of [296]). Regarding other potential contributions to this mass flux, Rafikov [250] argues against the Yarkovsky effect being significant (in his section 2.2).

*SB accretion frequency.* The frequency and size distribution of accreting SBs depend on the architecture of the planetary system and what instabilities it undergoes throughout its lifetime. Despite the vast number of free parameters in this problem, several investigations have related WD accretion frequencies to particular setups. I have already discussed studies that have modelled architectures involving planets and asteroids in §7. Here I present three studies [67,106,156] dedicated to determining accretion rates onto WDs from exo-Oort cloud comets. These studies do not actually involve *N*-body interactions, as the comets are assumed to be point masses and instability is caused by other means (primarily stellar mass loss, Galactic tides and stellar flybys).

Both Veras *et al.* [106] and Alcock *et al.* [156], written almost three decades apart and through different means, came to the same conclusion: the cometary accretion rate onto WDs is about one per 10^4^ years. Both apply isotropic GB mass loss with no natal kick, but the former uses a ramp function prescription (their eqn 13) and the latter adopts prescriptions from a stellar evolution code. The former does not incorporate Galactic tides and stellar flybys, but the latter does. These variations are perhaps offset by the different assumptions that the studies adopted for stellar masses and MS Oort cloud architectures. One other perhaps important difference is that Veras *et al.* [106] adopt an escape ellipsoid surrounding the primary such that a comet is assumed to escape even if it enters the ellipsoid without an osculating parabolic or hyperbolic orbit.

In contrast with these two studies, Stone *et al.* [67] incorporated a natal kick that accompanies GB mass loss, and assumed that the mass loss is instantaneous. They modelled the stellar mass to change from 1.2 *M*_⊙_ to 0.6 *M*_⊙_, and the star to be kicked with velocities ranging from 0.05 km s^−1^ to 4.00 km s^−1^. They considered the effect on exo-Oort clouds with *a*^(MS)^=[1×10^3^,5×10^4^] AU. This range is particularly interesting because it lies at the limit between adiabaticity and non-adiabaticity in the non-instantaneous mass ejecta case [208,210]. One can observe the straddling of this boundary in fig. 3 of [67]. They ultimately find that this combination of instantaneous mass loss and kick succeeds in producing comets on near-parabolic orbits so that at least some will enter the ice line and sublimate.

None of these three studies modelled interstellar comets. Veras *et al.* [106] justify this neglect (see their section 3) by (i) estimating that a typical exo-Oort cloud should be a few orders of magnitude more dense than the interstellar medium, (ii) estimating that Oort cloud comet velocities are likely to be less than an order of magnitude smaller than those of interstellar comets and (iii) pointing out that no Sun-grazing comet has ever been observed to harbour a hyperbolic orbit.

*Spectral simulations.* An exciting alternative, complementary method to measure the chemical composition of planetary remnants through accreted material is to derive the chemical composition of the observed dusty [118,119] and gaseous [310,311] components of WD discs (as opposed to the atmospheric pollution) by simulating their spectra. However, successful models for the gaseous discs require knowing the origin of the heating, particularly for the Ca II emission lines. The accretion rate derived by Hartmann *et al.* [310] (10^17^−10^18^ *g* s^−1^) is too high by several orders of magnitude to match observations. For dusty discs, fig. 14 of [88] compared abundances derived from infrared spectra and atmospheric analyses; the James Webb Space Telescope (JWST) should provide much better constraints.

## 12. Other dynamics

### 12.1 General relativity

The sub-AU scales of WD debris discs, and the multi-AU scales of the orbits of disc progenitors, suggest that general relativity (GR) may play a significant role in the secular evolution of remnant planetary systems. GR does alter *a*, *e* and *ω* on orbital timescales [312], which can be important for volatile-rich SBs with orbital pericentres close to the disruption radius [254]. On secular timescales, *a* and *e* do not change, but *ω* famously does. GR will cause precession of the argument of pericentre over one full orbit on the timescale

12.1 $$\begin{array}{rl} t_{\omega }^{\left(\mathrm{GR}\right)} & \approx 107\;\mathrm{years}{\left(\frac{M_{⋆}}{0.60\;M_{⊙}}\right)}^{-3/2}{\left(\frac{a}{1.00R_{⊙}}\right)}^{5/2}(1-e^{2}) \end{array}$$

12.2 $$\begin{array}{rl} & \approx 0.15\;\mathrm{Myr}{\left(\frac{M_{⋆}}{0.60\;M_{⊙}}\right)}^{-3/2}{\left(\frac{a}{1.00\;\mathrm{AU}}\right)}^{5/2}\left[\frac{1-e^{2}}{1-0.999^{2}}\right]. \end{array}$$

Equations (12.1) and (12.2) may be useful, respectively, for WD debris discs and their potentially highly-eccentric progenitors.

### 12.2 Magnetism

*Unipolar inductors.* Magnetic WDs may provide newfound opportunities to detect post-MS planets. Willes & Wu [46,313] demonstrated how terrestrial planets orbiting WDs with a period of less than about 30 h may be detectable as electron-cyclotron maser sources. Both papers invoke the unipolar inductor model for WD–planet pairs [137], where a circuit is formed between the conducting planet (or planet core)—which would be inside of the WD magnetosphere—and the poles of the WD. Consequently, the WD atmosphere is heated. This scenario is visualized in figs 1–2 of [137], fig. 1 of [313] and fig. 1 of [46].

Within this framework, Willes & Wu [46,313] made an analogy with the detectable radio emissions from Jupiter due to its magnetic interaction with Galilean moons. For WD planetary systems, Jupiter would be replaced with the magnetic WD. The discovery space of WDs by this method is presented in fig. 2 of [46], and is a function of WD magnetic moment and SB orbital period.

One consequence of the unipolar inductor model is magnetically induced drag on the planet (or large enough SB to be a conductor). This interaction can cause an inward migration, with an inward drift speed of (eqn 7 of [137])

12.3 $$v^{\left(\mathrm{drift}\right)}=\frac{4Wr^{2}}{GM_{⋆}M_{\mathrm{SB}}},$$

where *W* is the heating rate associated with the current. Li *et al.* [137] claimed that the drift is negligible for $r≳200R_{⋆}$. Within this distance, the SB might already be undergoing tidal breakup (see §9). Whether there exist a planet-mass SB which represents this type of unipolar conductor orbiting the magnetic WD named GD 356 is an open question. If so, Wickramasinghe *et al.* [25] claimed that the planet is then likely to have a second-generation origin, in particular from the remnants of a WD–WD merger.

*Accretion onto compact objects.* Some metal-rich WDs are magnetic [105]. None of these stars have observed discs. Therefore, the pollution could arise from stochastic accretion of neutral material that is not affected by magnetism. Hollands *et al.* [105] asserted that rocky debris would be largely unaffected by even a large magnetic field, although Metzger *et al.* [296] claimed that a sufficiently strong field could affect solid body accretion by inductive coupling.

Alternatively, impactors might sublimate and produce charged gas. In order for this gas to be accreted, it must orbit faster than the magnetosphere at the Alfvén radius [194]. Further, this gas would be accreted along magnetic field lines. The Alfvén radius, *r*_Alf_, for a typical accreting WD is (eqn 63 of [296])

12.4 $$\begin{array}{rl} r_{\mathrm{Alf}} & \approx {\left(\frac{3B_{⋆}^{2}R_{⋆}^{6}}{2{\dot{M}}_{⋆}\sqrt{GM_{⋆}}}\right)}^{2/7} \end{array}$$

12.5 $$\begin{array}{rl} & \approx 1.2R_{⊙}{\left(\frac{B_{⋆}}{\mathrm{kG}}\right)}^{4/7}{\left(\frac{{\dot{M}}_{⋆}}{10^{8}\;{g\;s}^{-1}}\right)}^{-2/7}{\left(\frac{R_{⋆}}{0.01R_{⊙}}\right)}^{12/7}{\left(\frac{M_{⋆}}{0.6\;M_{⊙}}\right)}^{-1/7}, \end{array}$$

where *B*_★_ is the star’s magnetic field.

*Planetary atmospheres.* A planet’s or large moon’s magnetic field could act as a form of protection from erosion, which might otherwise proceed rapidly. Bear & Soker [289] posed that magnetic fields can suppress evaporation from an SB’s atmosphere due to post-MS stellar radiation. They claimed that this suppression will occur when the ram pressure of the outflowing gas is less than the magnetic pressure. This relation leads to the following condition for the evaporation to be suppressed (their eqn 4)

12.6 $$\begin{array}{rl} B_{\mathrm{SB}}^{\left(\mathrm{crit}\right)} & ≳\frac{2\sqrt{{\dot{M}}_{\mathrm{SB}}v_{\mathrm{wind}}}}{R_{\mathrm{SB}}} \end{array}$$

12.7 $$\begin{array}{rl} & \approx 5.5\times 10^{-3}\;\mathrm{kG}{\left(\frac{{\dot{M}}_{\mathrm{SB}}}{10^{10}\;{\mathrm{kg}\;s}^{-1}}\right)}^{1/2}{\left(\frac{R_{\mathrm{SB}}}{0.8R_{⊕}}\right)}^{-1}{\left(\frac{v_{\mathrm{wind}}}{2\times 10^{3}\;{m\;s}^{-1}}\right)}^{-1}, \end{array}$$

where *B*_SB_ is the SB’s magnetic field.

*Other effects.* Three other important points about magnetism are: (i) currents in the atmospheres of SB may be driven by electric fields which are induced by stellar winds crossing the magnetic fields [131], (ii) for CE evolution, as an SB companion spirals in, it could spin up the envelope, consequently more differential rotation could enhance magnetic fields, which could represent a driver for the mass loss [51], (iii) Nordhaus & Spiegel [242] speculated that if the stellar wind is magnetically coupled to the source, then this coupling might affect both the mass loss rate and the star’s moment of inertia. The latter could affect tidal interactions with SBs which are brown dwarfs or massive planets (see §5).

### 12.3 External influences

Planetary systems are not isolated entities. They are embedded within a galaxy and are subject to flybys and global tidal forces. These ‘external’ forces may play a crucial role in exosystem evolution. The larger the value of *r*, the greater chance the chance that an SB’s evolution will be affected by these external forces. Post-MS mass loss will increase the value of *r* by at least a few, and possibly by orders of magnitude. Therefore, an MS system not influenced by tides or flybys might become so after the parent star has become a WD or NS.

*Galactic tides.* Veras *et al.* [80] quantified external influences in the midst of post-MS mass loss. They found that AGB mass loss occurs on a short-enough timescale to be decoupled from the influence of Galactic tides (their fig. 1) but not necessarily stellar flybys. This result holds everywhere in the Milky Way galaxy. During the WD phase, however, Galactic tides can play a significant role [260]. Further, Galactic tides has its own ‘adiabatic’ regime, where adiabatic refers to conservation of semi-major axis, and the adiabatic/non-adiabatic regime is much further away (at approx. 10^5^ AU). Consequently, any SB near this boundary is already in the mass loss-based non-adiabatic regime. Their fig. 5 presents a flowchart of evolutionary possibilities, and their table 1 provides equations for the adiabatic and non-adiabatic SB evolution due to Galactic tides.

*Hill ellipsoid.* Galactic tides carve a region around the primary within which SBs orbit the primary and not the centre of the Galaxy. This region, which is not a sphere, is known as the Hill ellipsoid, with dimensions provided by eqns 21–22 of [80]. The Hill ellipsoid lies close to the adiabatic/non-adiabatic Galactic tidal boundary, and can help determine, for example, which Oort cloud comets escape before, during and after GB evolution [106]. For $M_{⋆}^{\left(\mathrm{MS}\right)}=2\;M_{⊙}$, about three-quarters of the volume of the entire MS Hill ellipsoid includes the semi-major axis range for which post-MS mass loss will cause an SB to escape.

*Stellar flybys.* Although stellar flybys are unpredictable, the stellar space density in the Milky Way is high enough to expect a close encounter at a few hundred AU over a typical Solar-like MS lifetime. The closer to the centre of Galaxy the star-SB system resides, the more susceptible it is to flybys. Fig. 3 of [80] quantifies the expected close encounter distance, and many of the above notions in this section; note that most known exoplanets reside at approximately 8 kpc from the Galactic centre.

*Wide stellar binaries.* Galactic tides trigger close encounters between wide binary systems for the first time many Gyr into the WD phase of the initially more massive star. This encounter in turn triggers instabilities in extant, and perhaps formerly quiescent, planetary systems around one or both stars [260]. This mechanism provides an avenue to pollute WDs which are many Gyr old. Importantly, the mass loss experienced in the post-MS evolution of the more massive star is adiabatic with respect to its daughter planetary system, but non-adiabatic with respect to the stellar binary companion. Consequently, the final super-adiabatic binary separation will be affected by Galactic tides and create eccentricity changes.

### 12.4 Climate and habitability

Can habitable climates exist on SBs orbiting compact objects? At least a few studies [143,145,242,314] have explored this possibility in some detail. In just about every case, the SB is assumed to have tidally circularized around the compact object. Agol [145] claimed that planets in the WD continuously habitable zones with orbital periods of ≈4−32 *h* will be circularized and tidally locked in ≈10−1000 years.

Fossati *et al.* [143] used a modified radiative transfer model to demonstrate that photosynthetic processes can be self-sustaining on planets in a continuously habitable zone around a non-magnetic WD. WDs provide stable luminosity sources with ultraviolet radiation doses which may be less damaging than those of MS stars. Although the WD luminosity continuously decreases, Fossati *et al.* [143] claimed that while a WD cools from 6000 K to 4000 K, a planet which is about 0.01 AU distant can remain in the habitable zone for about 8 Gyr, sufficiently long to allow complex life to develop.

Alternatively, Agol [145] performed computations of one-dimensional radiative–convective atmosphere models with water loss at the inner edge and a maximum carbon dioxide greenhouse at the outer edge. Barnes & Heller [314] adopted multiple tidal models, and found that a WD’s strong extreme ultraviolet emission is a significant barrier to sustain life. Nordhaus & Spiegel [242] estimated that if a planet is perturbed close enough to a WD to be tidally circularized (as in Veras & Gänsicke [144]), then the amount of orbital energy dissipated as heat, *I*, would be (their eqn 6)

12.8 $$I∼-4\times 10^{35}\;J\left(\frac{M_{⋆}}{0.6\;M_{⊙}}\right)\left(\frac{M_{\mathrm{SB}}}{M_{⊕}}\right){\left(\frac{a}{R_{⊙}}\right)}^{-1},$$

enough to scupper the prospects for habitability. Agol [145] also considered the prospects of other SBs delivering volatiles to a planet after it entered the WD habitable zone (a realistic possibility: see e.g. [67,106,156]), and Nordhaus & Spiegel [242] estimated that the impacts would impart orders of magnitude more of specific energy to those planets than the impacts which wiped out the dinosaurs.

Finally, Perets [279] mentioned that second-generation formation can provide new developmental pathways for habitable planets unavailable to first-generation planets, and Spiegel & Madhusudhan [131] speculated that a giant planet’s atmosphere could be carbon-enriched during the GB phases by accreting a stellar wind from a star that has undergone third dredge-up. Similarly, the chemical imprint of accretion in a terrestrial planet’s atmosphere during the GB phase might affect habitability during the WD phase.

## 13. The fate of the Solar system

The post-MS fate of the Solar system is of intrinsic human interest; Sackmann *et al.* [315] ‘could not resist exploring the Sun’s future’. However, the conclusions of the studies that investigated this future do not agree, highlighting the need for additional work in this area. The Solar system consists of four terrestrial planets, four giant planets, many asteroids, comets and moons, and many more smaller SBs. The gravitational influence of the Sun extends to 1×10^5^−3×10^5^ AU (eqns 21–22 of [80]), with objects likely existing out to that edge through the Oort cloud. In short, the fate of the Solar system is complex.

Tackling this problem requires all relevant forces to be brought to bear (figure 2). A planet will simultaneously expand its orbit, accrete stellar ejecta, evaporate from stellar radiation and be tidally influenced from Solar tides. Each planet will experience these effects to different extents, but are close enough to the Sun (a≲30 AU) such that external influences are negligible, and are large enough (*R*_SB_>10^3^ km) to not be bothered by stellar wind drag, the Yarkovsky or YORP effects.

*Remainder of main sequence.* Barring a close stellar flyby, the current gross architecture of the Solar system should be maintained throughout the remainder of the MS. Although the outer four planets will remain stable until the end of the MS, there is a one to few percent chance that the inner four planets will become collisional and/or unhinged [255,316]. The asteroid and Kuiper belt will likely continue to collisionally deplete.

*Mercury and Venus.* All studies agree that Mercury will be engulfed. The Sun’s surface will expand to such a great extent (to approx. 1 AU) that even Mercury’s expanded orbit (at approx. 0.8 AU) would lie within the Sun’s GB surface. Venus will almost certainly suffer a similar fate, despite the findings of Sackmann *et al.* [315], because of Sun–planet tides [229,230]. Because Sackmann *et al.* [315] did not incorporate tidal effects in their analysis, Venus’ expanded orbit (at approx. 1.5 AU) does escape the Sun’s reach in that (physically unrealistic) scenario.

*Earth.* The Earth’s fate is sensitive to both the Solar model and tidal model adopted. Schröder & Smith [230], using the updated Reimers mass loss prescription from equation (2.1) and the tidal formalism of Zahn [239,245], found that the Earth will be engulfed unless all tweakable parameters within the model were at one edge of their uncertainty ranges. They predict that Mercury, Venus and Earth will all be engulfed about 4.3, 1.5 and 0.5 Myr before the Sun’s RGB phase ends, which will occur about 7.59 Gyr from now. If Earth does survive, it won’t be habitable. The post-MS state of its atmosphere and surface has not though been modelled in detail. Schröder & Smith [230] do speculate that the Solar system’s habitable zone during the RGB tip would extend from about 49 to 71 AU, beyond the current location of Neptune.

*Outer 5 planets and asteroid belt.* Mars and the giant planets will survive, but the asteroid belt will likely not. Dynamically, the giant planets will not undergo instability [223] and experience a quiescent existence around the Solar WD. Mars should also survive dynamically because the semi-major axis ratio of Jupiter and Mars is greater than 3, and that ratio will be maintained during post-MS evolution due to adiabatic mass loss. Physically, how these five planets will fare has only started to be explored. Villaver & Livio [52] warned that planets in Jupiter’s location might undergo significant evaporation. Spiegel & Madhusudhan [131] specifically considered the fate of Jupiter’s atmosphere: how it is chemically and thermally altered by Solar wind accretion and the Sun’s GB radiation. SBs in the asteroid belt will either be evaporated or spun up to fission [251] given their close proximity to the Sun.

*Moons.* The moons of the Solar system have diverse physical characteristics and orbit their parent planets out to about half of one Hill radius. These orbits will become more stable after the Sun has become a WD because of the effect of mass loss on Hill radius (see equation (7.5)) and because the remaining planets are not expected to undergo potentially disruptive scattering [268]. The consequences of GB radiation on the ice crusts and oceans of moons like Europa and Enceladus remain unexplored.

*Kuiper belt and scattered disc.* How the Kuiper belt and scattered disc will be altered by post-MS Solar evolution remains unclear. The re-distribution of Kuiper belt objects due to the Yarkovsky effect, even if slight, may affect the edges of the belt and the resonant characteristics of many of these objects with Neptune. Consequently, the extent of the depletion [59,61,259] might change from current levels. An expanded scattered disc would be more susceptible to dynamical reshuffling from stellar flybys, particularly over several Gyr during the Solar WD phase (fig. 3 of [80]). The critical MS distance at which scattered disc objects might escape during post-MS phases is between 10^3^ and 10^4^ AU [210].

*Oort cloud.* The Oort cloud will be dynamically excited by the combination of stellar mass loss, orbital expansion, Galactic tides and stellar flybys [80]. The latter two will change from their present day prescriptions because the Milky Way and the Andromeda galaxies will collide before the Sun turns off of the MS [317]. Veras *et al.* [106] and Alcock *et al.* [156] disagree on the extent to which Oort clouds would be depleted during the GB phase, but interestingly are in rough agreement about the number of these comets reaching the inner Solar system (approx. 1 in every 10^4^ years). The distribution of ejected comets would not necessarily scale with semi-major axis [210] and the subsequent interactions with surviving planets has not been considered in detail.

## 14. Numerical codes

Post-MS planetary science has benefited with improvement in numerical tools since the discovery of the PSR 1257+12 planets. The two most widely used classes of tools for this science are stellar evolution codes and *N*-body codes. In table 2, I list codes that have been used in references from this paper. Another class of codes not listed are model atmosphere codes, like the IRAP and La Plata codes used by Deal *et al.* [302].

Full-lifetime integrations over the entire MS, GB and much of the WD or NS phases remain computationally challenging for several reasons: (i) the simulations often require a method to combine an *N*-body code with a stellar evolution code, (ii) this combination should ensure that errors converge as timesteps decrease (consider fig. 1 of [24]), (iii) the additional relevant forces from figure 2 need to be included and (iv) the bodies (stellar and SBs) must be on sufficiently wide orbits, otherwise the simulations will take months or years of real time to finish. Fortunately, the transition from MS to WD expands the orbits of surviving SBs and decreases the parent mass, allowing for quicker integrations by an order of magnitude (see fig. 8 of [24]). Consequently, the shorter the MS lifetime, and the larger the value of $M_{⋆}^{\left(\mathrm{MS}\right)}$, the more quickly the simulations will finish. Although energy is not a conserved quantity, angular momentum typically is, and represents an important benchmark for accuracy. Strong close encounters between SBs increase angular momentum errors (footnote #7 of [144]).

Two recent *N*-body codes which may prove useful for future studies are the genga code [328] and ias15 code [329]. genga uses graphical processing units (GPUs) to speed up computations. They claim their code runs up to 30 times faster than Mercury and 8 times faster than pkdgrav2, and can handle up to 2048 massive bodies or 10^6^ test particles. IAS15 is a 15th-order *N*-body integrator that can handle close encounters, high-eccentricity orbits and non-conservative forces, with systematic errors below machine precision. Section 1 of Rein & Spiegel [329] usefully explains the concept of symplecticity, and how symplectic integrators encounter difficulties when modelling binary systems or incorporating non-conservative forces.

## 15. Future directions

The fast-growing field of post-MS planetary science demands that theorists and observers work in concert to achieve shared goals, but should not obscure the importance of purely theoretical pursuits for such a dynamically rich and unexplored topic. In this section, I present ideas for future directions for both groups.

### 15.1 Pressing observations

#### 15.1.1 Continued monitoring of known objects

*WD 1145+017.* The one to several disintegrating SBs orbiting the polluted and disc-bearing WD 1145+017 [125] requires vigilant monitoring (i) first to constrain the number of SBs and their masses, (ii) to detect the onset of future breakup events from the large SBs, as they reside within the disruption radius, (iii) to determine how the surrounding disc varies due to direct injection of material and (iv) to detect variability in the accretion rates onto the WD. A chemical analogy may also be made with the planets breaking up around MS stars. Bochinski *et al.* [330] provided recent and strong evidence for a low-mass rocky planet disrupting around the MS star KIC 12557548 B, following up on the suggestion from Rappaport *et al.* [331]. Two other potentially disintegrating planets (KOI-2700b [332] and K2-22b [333]) raise the possibility even more that analysis of additional light curves from these systems could help measure grain composition of the broken-up debris.

*WD J0959-0200.* Continued monitoring of the variable disc around WD J0959-0200 [115] is important. Given that the flux dropped almost 35% within a 300-day period, there is a possibility that (i) instability in the disc will create an observable flare, as predicted by Bear & Soker [294] and (ii) photospheric abundances can be measured during the flux change, which will allow us to observe variability in accretion rates. Detecting variability in accretion onto DA WDs (like WD J0959-0200) may crucially constrain sinking timescales and disc lifetimes. Further, the WD J0959-0200 contains a gaseous component, and monitoring the gas and dust simultaneously might help us understand the interplay between dust, gas and variability.

*SDSS J1617+1620.* The variability of the gaseous component of the WD disc orbiting SDSS J1617+1620 since the year 2006 [334] generates the exciting possibilities that (i) potential future flare-ups due to repeated impacts of leftover debris from a tidal disruption event could be observed and (ii) we can see the inner rim of the disc moving outward through changes in the width of Ca II lines.

*SDSS J1228+1040.* Following the evolution of the axisymmetric and eccentric WD gaseous and dusty disc shown in figure 7 is particularly important to constrain WD disc evolution theory. Manser *et al.* [122] provided a clear prediction: In December 2016, the WD debris disc in SDSS J1228+1040 should reach halfway through its precession cycle.

*NN Ser.* Additional observations of NN Ser, particularly over the next couple years, will significantly help pin down the constraints for the number and properties of planets in this post-CE binary. The eclipse timing trajectory features a crest in the years 2019–2020 (fig. 9 of [175]) which will help confirm or refute the nature of the putative planets.

#### 15.1.2 Monitoring new objects

*Giant branch planets.* One of the most pressing theoretical concerns is the uncertainty about tidal dissipation mechanisms and their relation to planetary rheology. Planet-bearing GB systems can help us constrain some of the relevant physics. The tidally-based RGB study of Villaver *et al.* [228] illustrated that the region void of planets is too wide to have been depleted by tidal effects alone, providing us with a mystery. We need observations of greater numbers of GB planets in order to obtain a more detailed parameter distribution of these objects, and in particular to identify a cluster at the tidal disruption edge [242]. Separately, a greater sample of GB planets will help us resolve the difference in the planet–metallicity correlation in MS systems versus GB systems [168,169].

*Massive polluted white dwarfs.* How massive can host stars of exoplanets be? This fundamental question informs planetary formation and evolution, and current technology dictates that the answer is best obtained through observing high-mass WDs for signatures of pollution. For example, the polluted WD SDSS J1228+1040, which also happens to be the first WD discovered with a gaseous disc component, had a likely progenitor MS mass of $M_{⋆}^{\left(\mathrm{MS}\right)}∼4\;M_{⊙}$. No MS planet has yet been discovered around such a massive star.

*Magnetic polluted white dwarfs.* Planetary remnants, through split metal lines in the spectrum, help us understand magnetism in WDs, particularly in the oldest and coolest WDs. Therefore, we need to identify additional DZH stars [105], and further build up enough of a sample to distinguish the incidence of magnetic hydrogen-rich and magnetic helium-rich WDs. Further, a planetary core heating up a DH WD might have optically detectable H*α* emission [137].

*Binary systems with white dwarfs.* Kratter & Perets [277] explicitly suggested from their theoretical results that future surveys might find planets orbiting WDs in WD–MS binaries within the progenitor AGB radius. Further, Perets [279] claimed that WD–MS and WD–WD systems with separations of tens or hundreds of AU should be prime targets. He also provided specific examples of candidate second-generation exoplanetary systems. The likelihood of a second-generation disc formation scenario from binary stellar winds may be assessed from the frequency of wide WD companions to WD debris discs [285].

*James Webb Space Telescope.* Stone *et al.* [67] specifically studied the capacity of JWST to discover new WD debris discs, and found the prospects excellent (see their eqn 35). JWST observations of young WD debris discs could help distinguish an origin from exo-Oort cloud disruption versus from a remnant exo-Kuiper belt. The former would produce a different and detectable brightness profile (see section 4.4 and figs 7–9 of [67]). JWST, along with SPICA, should also find older, fainter discs [112]. Further, the European Mid-InfraRed Instrument on-board JWST will identify specific minerals in the debris orbiting WDs (currently G 29-38 is the only WD with a high-quality infrared spectrum; see fig. 4 of [119]), and this data can be combined with the known atomic abundances measured with HST. For pulsar planetary systems, Shannon *et al.* [186] suggested that tens to hundreds of hours of JWST time could detect the putative asteroid disc around B1937+21. Identifying the disc is particularly important partly because they suggest that second-generation circumpulsar planetesimal formation may be common; JWST observations will provide the timing precision ‘noise floor’, which in turn informs us about the stability of these stars. Finally, Wang [185] claimed that the ability of a pulsar to heat up circumstellar material is still unclear, and can be resolved with JWST along with WISE and the TMT.

*Other telescopes.* ALMA, due to its high spatial resolution, can directly resolve exo-Kuiper belts orbiting WDs [67]. Di Stefano [335] touted the potential of gravitational lensing to discover WD planetary systems, particularly with Pan-STARRS and LSST. The putative subdwarf planets inferred from pulsations may be confirmed or refuted with the PEPSI at LBT [179]. Gaia, LSST and PLATO will repeatedly (tens to thousands of times) survey 10^5^–10^6^ WDs, and by the year 2020 the combined efforts of the ESA missions Gaia and Euclid will increase the number of known WD debris discs by at least an order of magnitude. PLATO will find planets orbiting WDs. Gaia, LSST and WFIRST will help detect free-floaters, which may help constrain the fraction of ejected planets due to mass loss [80], particularly if high mass stars are targeted. Gaia will also provide better constraints on the wide binary population, potentially allowing for a test of the pollution mechanism suggested by Bonsor & Veras [260]. The SKA will also aid post-MS planetary science through the radio portion of the electromagnetic spectrum: the array will improve the chances of detection of radio emissions from WD–terrestrial planet systems [46] and help confirm the existence of circumpulsar discs [194].

*Other considerations.* The drastic increase then decline in luminosity from a tidal disruption event could create detectable outburst events. Bear & Soker [294] made a comparison with dwarf novae (see their table 1), which also create outbursts. Bear & Soker [290] proposed that young, hot WDs should ionize nebulae formed from an episode of AGB reincarnation due to engulfment of planetary hydrogen. Fossati *et al.* [143] suggested that polarimetry can be used to detect non-transiting WD habitable zone planets because the signal would be 2–5 orders of magnitude larger than for habitable planets around typical M-dwarf to Sun-like stars. Finally, exomoons in MS systems are on the verge of discovery; Pasqua & Khudhair [336] calculated the time-of-arrival signal that a hypothetical exomoon on an inclined orbit around a circumpulsar planet would induce.

### 15.2 Theoretical endeavours

Theoretical progress is lagging behind both the mounting observations of post-MS systems and the multitude of theory papers about MS planetary systems. Post-MS systems includes rich dynamics that is not found in MS systems (figure 2), and which needs to be explored in greater detail in order to understand physics and improve models.

*Effects of radiation.* Firstly, no study has yet self-consistently integrated the set of equations (6.1), (6.4), (6.5) and (6.6), or a similar set. Also, the applicability of the Yarkovsky geometry in equation (6.1) for highly eccentric objects is limited. In general, *w* may be a function of time, even for spherical SBs. We also know that asteroid-sized SBs are generally not spherical, and for those objects equation (6.1) should be modified. Further, equation (6.4) provides an averaged quantity. The high luminosity of GB stars and the duration of the SB orbit may require one to model spin changes on orbital rather than just secular timescales. We also need to characterize the debris fields produced from YORP spin-up of SBs so that we understand from where WD pollution arises, and can make a chemical link with first-generation formation. Equation (6.5) applies only for a single-species homogeneous sphere. We know that real asteroids are multi-layered, aspherical and contain many chemical species. In order to quantify the amount of water remaining in these SBs, more sophisticated models are required. Finally, the equations of motion for a moon (which orbits some SB) will be different than what is presented here, partly because of shadowing.

How do atmospheres change from post-MS stellar radiation? An important follow-up to previous studies [43,52,131] would be to solve the hydrodynamical equations for atmospheric escape. Further, these studies do not necessarily agree about the extent of Jupiter’s atmosphere that will escape. How much will Saturn, Uranus and Neptune be ablated? We know that the four giant planets in the Solar system will remain on stable orbits during the Solar GB phase, but how will they physically evolve?

*Effects of tides.* As is also true for MS tidal studies, tidal investigations for GB, WD and NSs can be improved by sampling tidal dissipation prescriptions for different planetary rheologies and stellar structures. Doing so will better constrain the timescales for SB destruction and the critical engulfment radius. Treating a population of known exoplanets with similar prescriptions (as in fig. 4 of [242]) might yield a very different result than a more individualized treatment of each planet (fig. 15 and table 2 of [337]). For GB systems, equations (5.1) and (5.2) need to be extended to higher SB eccentricities, and potentially coupled with stellar spin dynamics [242] at those higher eccentricities. For stars with similar radial extensions during both the RGB and AGB phases, different tidal prescriptions may apply [30,228] and might need to be treated together self-consistently within a single simulation.

*Effects of mass loss.* Compared with radiation and tides, our understanding of how SBs evolve due to stellar mass loss is better. Nevertheless, the consequences of accretion onto SBs has largely been ignored, as has the potential for GB stars to experience kicks during non-instantaneous mass loss. The effects of ram pressure and entrainment on smaller SBs such as boulders and pebbles need better quantification. How these effects, along with frictional drag, affect the transition between adiabatic and non-adiabatic motion is not yet clear. The transition itself, even in the non-accreting case, needs further exploration in order to explain e.g. the non-monotonic behaviour in fig. 10 of [210]. How resonances are formed, broken and maintained [215] may crucially affect WD pollution rates [59] and need further analysis on a commensurability-by-commensurability basis. Finally, simulations of the long-term MS future of the Solar system typically do not include effects from MS Solar mass loss. Although small, mass loss during the MS will cause a shift much greater than the instability-inducing 0.38 mm shift suggested by Laskar & Gastineau [255].

*Different planetary architectures.* So far, self-consistent full-lifetime numerical simulations of planetary systems have been limited in scope in terms of number of bodies, amount of time simulated and number of simulations. Room for improvement is significant, but must overcome computational hurdles. We need post-MS multi-planet simulations with an asteroid or Kuiper belt, a situation that will mirror the fate of our own Solar system. The planetary mass which maximizes the accretion rate onto the eventual WD is still unknown [61]. Where is the sweet spot for one-planet and other configurations? We need to determine the orbital and physical evolution of post-MS exomoons, Trojan asteroids and exorings, and how they will be subject to close encounters from other objects. We need to determine how fragments from SB–SB collisions evolve and are subject to scattering events. Mustill *et al.* [264] estimated through simulations of three planets only that no more than 1% of WDs should eventually host close-in giants planets. We must improve that estimate with (i) terrestrial-mass planets, (ii) unequal-mass planets and (iii) SBs smaller than planets. Also, further exploration of binary and triple stellar planet-hosting systems [62,280] will provide us with a more representative, population-based perspective on the evolution of planetary systems in the Milky Way.

Investigations detailing the fates of specific known exoplanetary systems are lacking. The famous HR 8799 system has a parent star with a progenitor mass that is typical of the currently observed WD population (figure 3) and represents a potential exemplar for polluted WD systems. The fates of other wide-orbit multi-planet systems on the current edge of MS stability (see e.g. section 6.3 of [24]) are not yet clear.

*Second-generation formation.* Currie & Hansen [287] called for more sophisticated extensions of their models of second-generation circumpulsar disc formation. In their section 7, they list several simplifications and assumptions adopted, which have not yet been lifted nearly a decade after that paper has been published. Many of the recent advancements in first-generation planet formation theory would also be applicable to second-generation SB formation.

*White dwarf debris disc formation and evolution.* The details of tidal disruption of an SB entering the WD Roche radius (and forming a disc) should be improved, particularly given the fine detail of some observations like those of Manser *et al.* [122] and in figure 7. As mentioned by Veras *et al.* [295], improved *N*-body rubble pile simulations may include ‘soft spheres’, with rolling and twisting friction between particles at multiple points of contact [338]. Alternatively, the spheres could have internal strength, or not be in the shapes of spheres at all. Movshovitz *et al.* [339] instead used polyhedral regular or or irregular grains to represent the building blocks of SBs. The SBs can be different shapes and spins, and when appropriate, harbour different equations of state [291]. Numerical modelling of the disruption of an incoming SB at an already existing disc would help characterize this likely scenario.

The evolution of the disc requires coupled modelling of the dust and gas, as in Metzger *et al.* [296]. They provided many useful relations which may be used for further modelling. Considering the simpler case of gas only, with potential self-generation of eccentricity and precession, is another approach which may help us disentangle different effects in these complex systems. Further work on spectral modelling of these discs [310,311] can link their composition with orbital evolution.

*WD accretion.* Improvements in atmospheric modelling will allow us to better distinguish accreted material from radiatively levitated material in hot WDs and from dredged-up carbon in cool WDs. In concert with observations, modelling the onset and tail-off of accretion events with different functional forms than equation (11.1) and as a function of chemical elements may help us better constrain progenitor masses and evaluate the steady-state assumption in DBZ WDs. Accretion from direct collisions between an SB and WD needs to be explored in more detail, to distinguish fragmentation, sublimation and explosion regimes.

*Finally.* Will the Sun become a polluted WD? The chances are good, with multiple surviving planets potentially perturbing Kuiper belt and asteroid belt fragments onto the Solar WD. However, other polluted WDs will have harboured different planetary architectures on the MS. Extrasolar planets, first unknowingly seen inside of a WD [3] and then by their own right orbiting a pulsar [1], have taught us that ‘their diversity cannot be easily foreseen from extrapolations of our knowledge of the Solar System’ [180].

## Footnotes

1 Sabine Reffert maintains this database at www.lsw.uni-heidelberg.de/users/sreffert/giantplanets.html.

2 Appropriately, after a distinguished career pioneering the dynamical field of periodic orbits, Hadjidemetriou returned to the topic of mass loss, and in his final paper [203] helped derived the anisotropic mass loss equations of motion and applied them to pre- and post-MS planetary systems. Confirmation of some of these equations can be found in Omarov’s work [197] in his eqn 13. For English-language context, see also eqns 19–24 of [204].
